# Tracking historical changes in perceived trustworthiness in Western Europe using machine learning analyses of facial cues in paintings

**DOI:** 10.1038/s41467-020-18566-7

**Published:** 2020-09-22

**Authors:** Lou Safra, Coralie Chevallier, Julie Grèzes, Nicolas Baumard

**Affiliations:** 1 Laboratoire de Neurosciences Cognitives, Département d’études cognitives, ENS, PSL, Research University, INSERM Paris, France; 2 Institut Jean Nicod, Département d’études cognitives, ENS, EHESS, PSL Research University, CNRS, Paris, France; 3 Sciences Po, CEVIPOF, CNRS, Paris, France

**Keywords:** History, Human behaviour

## Abstract

Social trust is linked to a host of positive societal outcomes, including improved economic performance, lower crime rates and more inclusive institutions. Yet, the origins of trust remain elusive, partly because social trust is difficult to document in time. Building on recent advances in social cognition, we design an algorithm to automatically estimate ratings of perceived trustworthiness evaluations from specific facial cues (such as muscle contractions associated with smiling) detected in European portraits in large historical databases. We used this measure as a proxy of social trust in history. Our results show that estimated levels of perceived trustworthiness in portraits increased over the period 1500–2000. Further analyses suggest that this rise of perceived trustworthiness is associated with increased living standards.

## Abstract (web summary)

Quantifying how social trust evolved throughout history can help us understand the long-run dynamics of our societies. Here, the authors show an increase in displays of trustworthiness, using a face processing algorithm on early to modern European portraits.

## Introduction

A number of historical observations suggest that social trust rose steadily in Europe from the early modern period onwards: religious tolerance increased, witch hunts abated, honor killings and revenge lost their appeal and intellectual freedom became a central value of modern countries^1,2^. Historians have used a range of cues to document this process: etiquette manuals, registries of friendly societies, or legal changes^1,3,4^. However, quantitative evidence is scarce and progress in the history of mentalities has been limited by the paucity of tools to capture people’s extinct mental life. Quite obviously, we cannot go back in time and ask people to fill out questionnaires or play economic games^5–7^ but we still have access to what their minds produced: books, songs, paintings, sculptures, etc. These cultural artefacts are the remnants of people’s past psychologies and can function as cognitive fossils of extinct mentalities and social preferences. Recent work has indeed shown that people’s preferences in various areas of social cognition are reflected in cultural artefacts: Costa and Corazza^8^ demonstrated that the people’s preference for friendly-looking faces leads painters to exaggerate “neotenic” features in their portraits (big eyes or round faces). Similarly, Morin^9^ has shown that direct-gaze Renaissance portraits are more popular than averted-gaze portraits. Fictions, such as romance novels^10^, TV shows^11^, epic poems^12^ or tragedies^13^, are all consistently aligned with humans’ universal interest for information related to mating, commitment and status competition for reviews and discussions, see refs. ^14,15^. These shifts in cultural artefacts reveal global changes in mentalities, reflecting the preference of the sitter, the artist and the audience altogether.

Portraits are particularly promising to document and quantify the level of perceived trustworthiness over time. Experimental work has revealed that specific facial features, such as a smiling mouth or wider eyes, are consistently used as cues for assessing perceived trustworthiness across individuals and cultures^16–21^. In this paper, we capitalize on this large empirical literature to build an algorithm that estimates the level of perceived trustworthiness based on a pre-identified set of facial characteristics^22^. More precisely, we apply recent machine-learning methods to extract quantitative information about the evolution of social cues contained in Western European portraits. The algorithm is built on models of human perception of faces to generate automatic human-like ratings of perceived trustworthiness ratings on portraits based on the muscle contractions (facial action units) detected in facial displays in portraits using the open software OpenFace^23^. Crucially, this algorithm does not provide information on a person’s face but rather on the way this face is likely to be perceived by others on a specific image. Indeed, first impressions from faces are highly sensitive to factors such as variations in lighting and pose. This algorithm was trained on avatars generated to display varying levels of perceived trustworthiness and optimized using a random forest procedure (see Supplementary Methods for more details). To assess the generalizability of our model, we then tested its validity on four databases of natural faces rated by real participants. We first demonstrated that the algorithm produced perceived trustworthiness ratings that were aligned with those produced by human participants in all four controlled databases. Another validation method would have been to also measure the correlation between the estimated perceived trustworthiness of the historical portraits calculated by our algorithm and the evaluations of the actual participants on these paintings. This other method has the major advantage of providing a direct test of the reliability of our algorithm. However, since participant evaluations of perceived trustworthiness may be influenced by historical cues not relevant to assess perceived trustworthiness (such as the sitter’s outfit or the painting style) that may bias these evaluations so that older portraits are perceived as less trustworthy, this method of validation is limited. Therefore, we chose to assess the validity and generalizability of our model independently of idiosyncratic biases of participants by relying on well-known effects in the literature, i.e., the effect of emotion, age, gender, and head orientation on facial evaluations^21,22,24–26^.

We thus checked that the algorithm was susceptible to the same biases as humans, i.e., rating younger, feminine, and happy faces as more trustworthy. Third, we checked that the output of the algorithm was robust to variations in head orientation^21,24^ (see Supplementary Methods for the results). We then replicated all these findings outside well-controlled databases by analyzing all the images (photographs and paintings) obtained from a Google image search for ‘women portraits’ vs ‘male portraits’ (*N* = 633; perceived trustworthiness: *t*(632) = 7.89, *p* < 0.001; perceived dominance: *t*(632) = −11.79, *p* < 0.001). This validation method provides evidence of the ability of our algorithm to produce human-like face evaluations on a large range of images (i.e., controlled photographs, natural photographs and paintings).

In this article, all occurrences of the words ‘trustworthiness’ and ‘dominance’ refer to subjective perceptions of trustworthiness and dominance from faces and not to individuals’ actual level of trustworthiness or dominance.

## Results

### Ratings of perceived trustworthiness in portraits increased throughout history

**Fig. 1:**
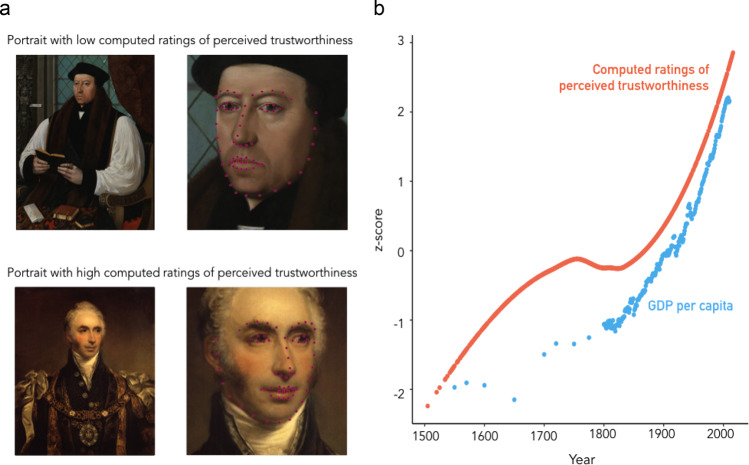
Evolution of ratings of perceived trustworthiness in England across time. **a** Example of faces detected in portraits from the National Portrait Gallery and estimated as being perceived as lowly trustworthy (top; Thomas Cranmer by Gerlach Flicke, 1545-1546, NPG 535 All rights reserved © National Portrait Gallery, London) and estimated as being perceived as highly trustworthy (bottom; Sir Matthew Wood by Arthur William Devis, 1815-1816, NPG 1481 All rights reserved © National Portrait Gallery). **b** Evolution of ratings of perceived trustworthiness in the National Portrait Gallery (for representation purposes, in this Figure, evaluations of perceived trustworthiness were fitted by a local polynomial regression with a span of 0.75 and adjusted for perceived dominance) and GDP per capita in England. (log-transformed for representation purposes). Source data are provided as raw data and scripts on the online depository.

**Fig. 2:**
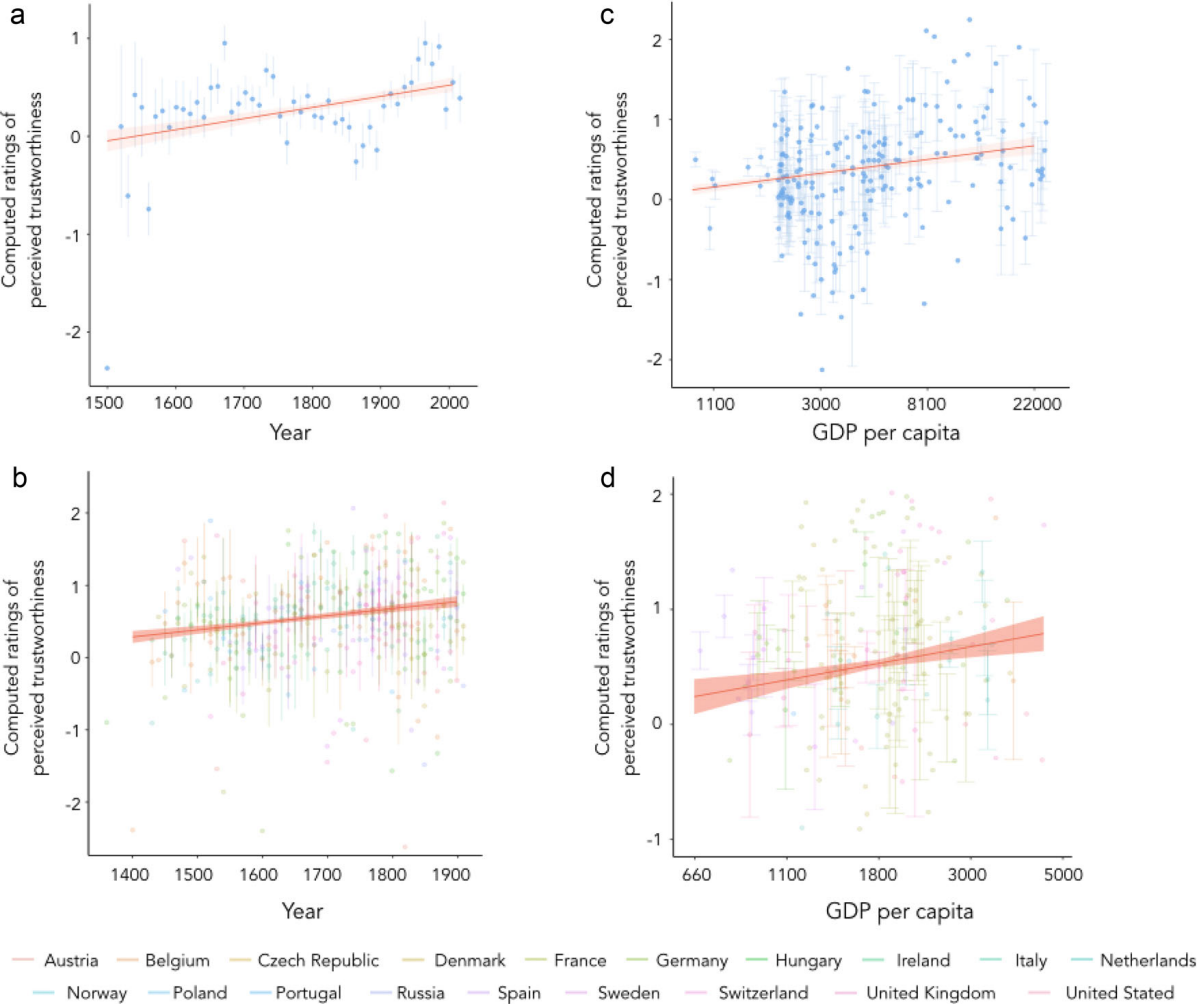
Effect of time and affluence on ratings of perceived trustworthiness across time. Time was associated with an increase of ratings of perceived trustworthiness displays in both the National Portrait Gallery (**a** data are aggregated by decade; regression line corresponds to the analysis on individual portraits) and the Web Gallery of Art (**b**)—data are aggregated country and by decades; regression line corresponds to the analysis on individual portraits). Increased GDP per capita predicted increased ratings of perceived trustworthiness displays better than time only-models both in the National Portrait Gallery (**c** data are aggregated by GDP; regression line corresponds to the analysis on individual portraits) and the Web Gallery of Art (**d** data are aggregated by country and GDP; regression line correspond to the analysis on individual portraits). Data are represented as mean values, error bars represent standard error to the means, the red line corresponds to the estimated effect in the regression adjusting for gender, age (for the National Portrait Gallery only) and perceived dominance, the shaded area represents the standard error to the mean of these effects. Source data are provided as raw data and scripts on the online depository.

To assess the evolution of perceived trustworthiness displays in history, we first analyzed the paintings of the National Portrait Gallery (Fig. 1a), the largest online database of historical portraits (analyzed *N* = 1962 English portraits from 1505 to 2016). Because perceived trustworthiness is correlated with perceived dominance^24^, all the analyses were controlled for perceived dominance. In line with historical work, we found a significant increase of perceived trustworthiness with time (*b* = 0.14 ± 0.02, *z* = 7.49, *p* < 0.001; Table 1; time coded such as one unit corresponds to 100 years, ±corresponds to standard errors to the mean; Figs. 1b and 2a), suggesting that the value of interpersonal trust increased from the 16^th^ to the 20^th^ century. We then replicated our findings on the Web Gallery of Art, an important fine art repository (*N* = 4106 portraits) spanning 19 Western European countries seven centuries (1360–1918) and found a significant increase in perceived trustworthiness displays with time (*b* = 0.07 ± 0.01, *z* = 5.33, *p* < 0.001; Table 1; Fig. 2b). Although the increase of perceived trustworthiness is small, these results are consistent with more qualitative works documenting a so-called ‘Smile Revolution’^27^ and a rise of prosocial displays in paintings and in novels^28^. It is worth noting, however, that the historical increase in perceived trustworthiness observed in our datasets parallels the rise of liberal values such as religious tolerance, political freedom and democracy^2,29,30^.

**Table 1:** Effect of time, GDP per capita and democratization on ratings of perceived trustworthiness in the portraits of National Portrait Gallery and the Web Gallery of Art.

Time only			Affluence only		Time + Affluence		Democratization only		Time + Democratization	
	National Portraits Gallery	Web Gallery of Art	National Portraits Gallery	Web Gallery of Art	National Portraits Gallery	Web Gallery of Art	National Portraits Gallery	Web Gallery of Art	National Portraits Gallery	Web Gallery of Art
Year	**0.14** ± **0.02** ***z*** = **7.49 p** < **0.001**	**0.07** ± **0.01** ***z*** = **5.33** ***p*** < **0.001**			**0.08** ± **0.03** ***z*** = **3.17** ***p*** = **0.002**	**0.06** ± **0.02** ***z*** = **2.87** ***p*** = **0.007**			**0.32** ± **0.11** ***z*** = **2.86** ***p*** = **0.004**	−0.13 ± 0.14 *z* = −0.98 *p* > 0.250
GDP per capita			**0.03** ± **0.00** ***z*** = **7.13** ***p*** < **0.001**	**0.09** ± **0.03** ***z*** = **3.16** ***p*** = **0.002**	**0.02** ± **0.01** ***z*** = **3.16** ***p*** = **0.002**	**0.07** ± **0.04** ***z*** = **1.98** ***p*** = **0.048**				
Democracy index							**0.03** ± **0.01** ***z*** = **5.24** ***p*** < **0.001**	−0.01 ± 0.01 *z* = −1.96 *p* = 0.051	−0.01 ± 0.01 *z* = −0.50 *p* > 0.250	−0.01 ± 0.01 *z* = −0.96 *p* > 0.250
Perceived dominance	−0.79 ± 0.02 *z* = −40.74 *p* < 0.001	−0.74 ± 0.01 *z* = −56.58 *p* < 0.001	−0.78 ± 0.02 *z* = −40.10 *p* < 0.001	−0.75 ± 0.02 *z* = −46.29 *p* < 0.001	−0.78 ± 0.02 *z* = −40.30 *p* < 0.001	−0.74 ± 0.02 *z* = −46.05 *p* < 0.001	−0.77 ± 0.03 *z* = −30.76 *p* < 0.001	−0.71 ± 0.04 *z* = 20.17 *p* < 0.001	−0.77 ± 0.03 *z* = −30.83 *p* < 0.001	−0.71 ± 0.04 *z* = −20.17 *p* < 0.001
Gender	0.32 ± 0.06 *z* = 5.64 *p* < 0.001	0.31 ± 0.03 *z* = 10.76 *p* < 0.001	0.29 ± 0.06 *z* = 5.01 *p* < 0.001	0.30 ± 0.04 *z* = 8.31 *p* < 0.001	0.30 ± 0.06 *z* = 5.10 *p* < 0.001	0.29 ± 0.04 *z* = 7.98 *p* < 0.001	0.28 ± 0.08 *z* = 3.61 *p* < 0.001	0.25 ± 0.07 *z* = 3.30 *p* = 0.001	0.25 ± 0.08 *z* = 3.16 *p* = 0.002	0.25 ± 0.07 *z* = 3.37 *p* < 0.001
Age	−0.00 ± 0.00 *z* = −2.03 *p* = 0.043		−0.00 ± 0.00 *z* = −1.88 *p* = 0.060		−0.00 ± 0.00 *z* = −2.26 *p* = 0.024		0.00 ± 0.00 *z* = 0.48 *p* > 0.250		−0.00 ± 0.00 *z* = −0.17 *p* > 0.250	
Sample (*N*)	1962	4106	1943	2706	1943	2706	1115	565	1115	565

The first line corresponds to the regression coefficient with their associated standard error to the mean (mean ± s.e.m.). Results in bold corresponds to statistically significant effects of the variables of interest. The upper part of the table presents the effects of the variables of interest (time, affluence and democratization), while the lower part presents the effects of the control variables (Perceived dominance, gender and age). All the tests are two-sided. Following APA’s recommendations, exact *p*-values are provided for p-values between 0.001 and 0.250. Source data are provided as raw data and scripts on the online depository.

Whether such increased perceived trustworthiness in portraits parallels an actual shift in social trust remains an open question. To assess the validity of this assumption, we applied our algorithm to selfies posted on Instagram in six cities around the world in 2013 (Bangkok, Berlin, London, Moscow, New York and Sao Paulo; SelfieCity database, pictured analyzed *N* = 2277^31^), we found that people located in places where interpersonal trust and cooperation are higher (as assessed in the European and World Value Surveys^32,33^) displayed higher levels of perceived trustworthiness in their selfies (cooperation level: *b* = 0.13 ± 0.03, *z* = 3.67, *p* < 0.001; trust level: *b* = 0.81 ± 0.23, *z* = 3.50, *p* < 0.001; ±corresponds to standard errors to the mean; Supplementary Figure 6). Together, this suggests that the display of trustworthiness in portraits can indeed be used as a reliable proxy of the level of social trust in individuals’ environment^34,35^.

### Ratings of perceived trustworthiness in portraits increased with affluence

Another open question is that of the potential predictors of perceived trustworthiness fluctuations in social displays. We first examined the role of resources. Trust can indeed be construed as an investment in social interactions with potential benefits (in the event of cooperation) and also potential losses (in the event of defection). Because losses have more dramatic effects for poorer individuals, individuals with lower resources are arguably more exposed by exploitation risks and should therefore have lower levels of social trust^36^. In line with this reasoning, international surveys show a strong association between resources and social trust^37–40^. Moving beyond correlations, economists have recently demonstrated that childhood resources had a causal impact on adult trust levels using exogenous variations in caloric rationing in post WW2 Germany^41^.

This is particularly relevant in light of the fact that the Middle Ages and the early Modern Period were periods of prolonged economic growth for Europe in general and England in particular^42,43^. We thus tested whether higher GDP per capita was associated with the rise of perceived trustworthiness in portraits. Our analysis of the National Portraits Gallery database revealed an association between higher levels of affluence and higher levels of perceived trustworthiness between the 16^th^ and the 21^st^ centuries (*b* = 0.03 ± 0.01, *z* = 7.13, *p* < 0.001; Table 1; Fig. 2c), even after adjusting for a monotonous effect of time (*b* = 0.02 ± 0.01, *z* = 3.16, *p* = 0.002; Table 1). Crucially, GDP per capita accounted for the evolution of perceived trustworthiness better than a monotonous effect of time (Bayes Factor: 3.38), which suggests that the observed evolution of perceived trustworthiness cannot be reduced to a simple cultural accumulation that would have led to the development of painting techniques making sitters look more trustworthy. We then sought to replicate this result in the Web Gallery of Art database and also found a significant positive association between GDP per capita and perceived trustworthiness (*b* = 0.09 ± 0.03, *z* = 3.16, *p* = 0.002; Table 1; Fig. 2d). This association was robust to adjusting for a monotonous increase of perceived trustworthiness over time (*b* = 0.07 ± 0.04, *z* = 1.98, *p* = 0.048; Table 1). Again, the model including GDP per capita provided a better account of the variations of perceived trustworthiness than time alone (Bayes Factor: 130.16).

Institutional change is another possible predictor of increased trust. The establishment of more democratic, more inclusive and more egalitarian institutions might indeed have created a climate of trust and tolerance^44,45^. We tested this idea by measuring the association between perceived trustworthiness in paintings and political democratization using the Polity2 index (a composite measure of institutionalized democracy and autocracy available from 1800, see Supplementary Methods). Although a significant association was found between these two variables in the National Portraits Gallery (*b* = 0.03 ± 0.01 *z* = 5.24, *p* < 0.001), this effect was not robust to the inclusion of time as covariate (*b* = −0.01 ± 0.01, *z* = −0.50, *p* > 0.250) and the evolution of perceived trustworthiness was better explained by GDP per capita than by changes in the institutions (Bayes Factor: 2.75). Moreover, the positive association between more democratic institutions and higher perceived trustworthiness was not replicated in the Web Gallery of Art sample (*b* = −0.01 ± 0.01 *z* = −1.96, *p* = 0.051; with time as a covariate: *b* = −0.01 ± 0.01 *z* = −0.96, *p* > 0.250; Bayes Factor of the GDP per capita model compared to the democratic institutions model: 6.16).

These results provide evidence in favor of the association between economic wealth and social trust at the society level. However, due to the small effect sizes and the limitations of the historical economic indicators^46,47^, as well as to the fact that GDP per capita is only a partial measure of wealth (which does not account, for example, for inequalities in wealth distribution^48^), we replicated our analyses with an alternative variable known to be associated with countries’ wealth: the number of book titles per capita. Indeed, although the number of book titles per capita is thought to be linked to human development variables, it has also been shown to be associated with national income^48–51^. Supporting the analyses conducted with GDP per capita, we found a significant positive association between the number of book titles per capita and the level of perceived trustworthiness in the portraits of the National Portrait Gallery (affluence only model: *b* = 0.35 ± 0.06, *z* = 6.15, *p* < 0.001; model controlling for time: *b* = 0.21 ± 0.06, *z* = 3.45, *p* = 0.001) and of the Web Gallery of Art, although not robust to the inclusion of time in this latter case (affluence only model: *b* = 0.29 ± 0.10, *z* = 2.77, *p* = 0.006; model controlling for time: *b* = 0.14 ± 0.11, *z* = 1.26, *p* = 0.208).

### Changes in affluence precede changes in ratings of perceived trustworthiness in portraits

Demonstrating that the association between GDP and the rise of perceived trustworthiness is causal would of course require additional data. Based on our dataset however, we were able to investigate the dynamics of these historical changes by running time-lag analyses on perceived trustworthiness and GDP per capita. We found that changes in GDP per capita predicted future changes in perceived trustworthiness in the National Portraits Gallery two decades later (*F*(40,1) = 12.38, *p* = 0.001) while changes in political institutions did not (*F*(15,1) = 0.11, *p* > 0.250). The effect of GDP per capita on perceived trustworthiness was generalizable to the other European countries (Web Gallery of Art sample, effect of GDP 20 years before on perceived trustworthiness *X*(1) = 6.42, *p* = 0.011; Institutions 20 years before: *X*(1) = 0.81, *p* > 0.250). Importantly, changes in perceived trustworthiness did not predict future changes in GDP per capita either in the National Portraits Gallery sample (*F*(41,1) = 0.76, *p* > 0.250) or in the Web Gallery of Art dataset (*X*(1) = 2.02, *p* = 0.155), which suggests that changes in GDP per capita may have preceded changes in perceived trustworthiness in this dataset. This conclusion is consistent with other works emphasizing the importance of economic growth and psychological changes in history^52–54^.

## Discussion

The algorithm was built to estimate how human raters would rate the perceived trustworthiness of faces. It can be used in scientific research for this purpose. The algorithm does not quantify the actual trustworthiness of an individual, and was not intended for this purpose.

To conclude, our analyses—replicated across two independent fine arts databases—reveals that perceived trustworthiness increased in early modern period portraits and are suggestive of an actual shift in social trust over the period (although differences across countries might have persisted over the period, see refs. ^5–7^). This cultural shift is more strongly associated with GDP per capita than institutional change.

At this point, it is important to note the small correlation between the perceived trustworthiness ratings provided by human raters and those retrieved by our algorithm. However, this small effect size is to be expected. First, the avatars on which the algorithm was trained did not represent the texture of the faces, even though this information may influence human raters’ evaluations. Similarly, the avatars are bold and our algorithm is thus blind to haircut, even though these cues are known to influence first impressions from faces (see e.g.,^21^). Finally, our algorithm was trained to generate ratings of perceived trustworthiness based on the facial features that represent the shared component of first impressions from faces. Indeed, individuals rely on both shared and idiosyncratic features when forming a first impression on a new face, and our algorithm was designed to produce scores only based on the former. Finally, several limitations are to be noted. First, one cannot assume that the evolution of perceived trustworthiness depicted in this study extends to the larger population of the period. The phenomenon described in this article might, for instance, be limited to the relatively elite, wealthy population represented in the portraits. In line with this possibility, there is evidence that social attitudes can vary with socioeconomic status^55–58^. Second, our study is based on the assumption that facial cues that are used as cues to assess perceived trustworthiness are shared across time. Although recent evidence^59–61^ points towards such a stability, further work is needed to fully test this assumption. Third, times series of GDP per capita and living standards are only estimates, and their precision may fluctuate throughout the studied time period and fail to fully capture the evolution of living standards and inequalities^46–48^.

These findings complement existing qualitative historical accounts and demonstrate how insights from cognitive sciences can enrich our understanding of cultural evolution.

## Methods

### Construction of an algorithm for modeling ratings of perceived trustworthiness and ratings of perceived dominance evaluations

We built a model that automatically extracts ratings of perceived trustworthiness and perceived dominance evaluations from the all the facial action units detected by the OpenFace algorithm (i.e., both dichotomous and continuous estimations; OpenFace version 1.01 using OpenCV 3.3.0^62^). To do so, we extracted the facial action units of five sets of avatars previously generated with Facegen and controlled for perceived dominance, for perceived trustworthiness or for both (Supplementary Fig. 1)^63^. Each avatar is generated from an initial face and manipulated to either express a specific level of perceived dominance, perceived trustworthiness or both based on the model developed by Oosterhof and Todorov^24^. These avatar faces have been shown to successfully elicit ratings of perceived dominance and perceived trustworthiness in participants^63–65^. Thus, compared to participants’ ratings on photographs that may be sensitive to the participants characteristics and to experimental protocol factors (such as the type of scale used to give the ratings), using avatars allow us to have well-validated sets of faces to train our model. These sets of avatars correspond to all the existing and available validated avatars controlled for perceived trustworthiness or perceived dominance and generated by Facegen.

3% of the faces were excluded from the modeling process for not having been accurately detected by OpenFace. The total sample of avatar faces were then split in a training sample (80% of the faces) and a test sample (20% of the faces). Importantly, the percentage of avatars coming from each avatar set was equal in the training and test samples for both perceived trustworthiness and perceived dominance (perceived Trustworthiness: *X*^2^(2) = 0.02, *p* > 0.250; perceived Dominance: *X*^2^(2) = 0.01, *p* > 0.250).

To determine which type of algorithm (linear model, random forest model from the RandomForest R package^66^—Breiman’s random forest algorithm^67^—or support vector model either linear or radial from the kernlab R package^68^) would provide the most accurate evaluations, we ran a repeated 20-folds cross-validation (five repetitions) on the training test of each of these models separately for perceived dominance and perceived trustworthiness using caret R package^69^. Each model’s hyperparameters were optimized using a random search. The hyperparameters optimized for each model are presented in Supplementary Table 1. This analysis revealed significantly better performance for the random forest model than for the linear model and the linear SVM model in terms of mean absolute error, root square mean error and r-squared and was and better than, for the perceived trustworthiness model, and similar to, for the perceived dominance model, the radial SVM model (Supplementary Table 1). For both perceived trustworthiness and perceived dominance, the optimal *m*_try_ hyperparameter of the random forest models was found to be equal to 9, corresponding to setting the number of variables to consider at each tree to 9. We then tested the predictions of the random forest model with this optimal hyperparameter obtained by cross-validation on our perceived trustworthiness and perceived dominance test sets. This test revealed a high performance of the model (perceived trustworthiness: *r* = 0.85 ± 0.5, *t*(75) = 14.17, *p* < 0.001; perceived dominance: *r* = 0.86 ± 0.05, *t*(75) = 14.72, *p* < 0.001; Supplementary Fig. 2; all the reported statistical tests are two-sided).

### Validation of the algorithm for modeling ratings of perceived trustworthiness and ratings of perceived dominance evaluations

To assess the accuracy our perceived trustworthiness and our perceived dominance generator algorithm, we tested their predictions on four different face databases: the Karolinska database (*N* = 70 distinct faces)^70^, the Oslo Face database (*N* = 185 distinct faces)^71^, the Chicago database (*N* = 520 distinct faces)^72^ and the FEI Face database (*N* = 520 distinct faces)^73^. Given that our model was optimized on avatar faces, comparing our model’s prediction to real participants ratings in a second step allows us to assess whether our model would give overall coherent ratings with those of real human beings. Our first analysis confirmed the significant correlation of the modeled perceived trustworthiness and perceived dominance estimates with the actual participants’ ratings of perceived trustworthiness and perceived dominance ratings on the faces from these databases (except the FEI Face database which did not provide subjective ratings; Supplementary Figure 3). We found significant correlations for both perceived trustworthiness and perceived dominance estimates (perceived trustworthiness: *r* = 0.22, *p* < 0.001, perceived dominance: *r* = 0.16, *p* < 0.001—*N* = 768 for each correlation, to not artificially increase the statistical power of this analysis only the neutral and facing version of the faces were used for these correlations), confirming that our model gave perceived trustworthiness and perceived dominance estimates that are coherent with real participants’ evaluations on these traits.

Going one step further, we assessed whether our modeled perceived trustworthiness and perceived dominance were able to reproduce classical findings in social cognition on perceived trustworthiness and perceived dominance, namely: gender effect (females appear as less dominant and more trustworthy than males; perceived trustworthiness: real effect: *t*(768) = 7.94, *p* < 0.00; recovered effect: *t*(972) = 2.67, *p* = 0.008; perceived dominance: real effect: *t*(769) = −7.80, *p* < 0.001; recovered effect: *t*(972) = −3.63, *p* < 0.001; Supplementary Fig. 4A, B), emotion effects (angry faces appear as more dominant than neutral faces: *t*(167) = 9.42, *p* < 0.001; happy faces appear as more trustworthy than neutral and angry faces: *t*(167) = 10.64, *p* < 0.001; Supplementary Fig. 4C, D), head orientation effects (perceived trustworthiness and perceived dominance evaluations for a unique identity are correlated across head orientations: perceived trustworthiness: *r* = 0.29, *t*(1500) = 11.51, *p* < 0.001; perceived dominance: *r* = 0.34, *t*(1500) = 13.79, *p* < 0.001; Supplementary Fig. 4E, F) and age effect (older adults appear as more dominant and less trustworthy than younger adults: perceived trustworthiness: real effect: *r* = −0.12, *t*(518) = −2.75, *p* = 0.006; recovered effect: *r* = −0.12, *t*(518) = −2.68, *p* = 0.008; perceived dominance: real effect: *r* = 0.32, *t*(518) = 7.72, *p* < 0.001; recovered effect: *r* = 0.16, *t*(518) = 3.70, *p* < 0.001; Supplementary Fig. 4G, H)^21,24–26^.

All these effects were replicated with the modeled evaluations of perceived trustworthiness and perceived dominance evaluations. In addition, although perceived dominance and perceived trustworthiness were modeled independently, we also replicated the classical correlation between these two traits, further suggesting the importance of investigating perceived trustworthiness conjointly with perceived dominance (effect on participants’ ratings: *r* = −0.21, *t*(768) = −5.81, *p* < 0.001; retrieved effect by our algorithm *r* = −0.46, *t*(768) = −14.30, *p* < 0.001).

Importantly, we further validated our model by replicating the gender effect on all the portraits extracted from a Google image search for ‘women portraits’ vs ‘male portraits’ containing both pictures and paintings (*N* = 633; perceived trustworthiness: *t*(632) = 7.89, *p* < 0.001; perceived dominance: *t*(632) = −11.79, *p* < 0.001; Supplementary Fig. 5A, B). We also replicated the gender effect on the official portrait pictures of US representatives (*N* = 419; gender: perceived trustworthiness: *t*(417) = 2.20, *p* = 0.028, perceived dominance: *t*(417) = −4.74, *p* < 0.001; Supplementary Fig. 5C, D). Importantly, we also replicated the effect found in the literature that conservative representative appear more dominant than democrat representatives (*t*(417) = −2.59, *p* = 0.009; Supplementary Fig. 5E).

### Testing the relationship between interpersonal trust and portrait Selfies’ ratings of perceived trustworthiness

We tested whether perceived trustworthiness could be used as a proxy for interpersonal trust. To do so, we analyzed the Selfiecity database^31^ which includes 3230 selfies posted on Instagram in 2013 from six cities across the world (Bangkok, Berlin, London, Moscow, New York and Sao Paulo; analyzable images: *N* = 2277^31^).

The identified faces were then individually analyzed by two independent raters who were asked to evaluate, for each picture, the alignment of the OpenFace’s face identification points compared to the real face’s contours (coded as 0 or 1). The sum of these goodness of fit was then used as weights for the analyses. Therefore, only faces for which the two raters agreed that they were not well detected were removed from the analyses. Faces for which the two raters agreed on their good detection had a weight of 2 in the analyses, and those on which they disagreed had a weight of 1.

Importantly, a preliminary analysis confirmed that the perceived trustworthiness computed with our algorithm recovered the gender effect documented in the literature in this image sample too (perceived trustworthiness: *t*(2275) = 13.80, *p* < 0.001; perceived dominance: *t*(2275) = −10.18, *p* < 0.001; Supplementary Fig. 6A, B). Extracted perceived trustworthiness was analyzed using a linear model taking the sitter’s gender, the city longitude and latitude and the sitter’s perceived dominance as control variables. The effect of two measures of interpersonal trust were used to assess the link between perceived trustworthiness and interpersonal trust, extracted from the European and World Value Surveys^32,33^ general social trust question (‘most people can be trusted or you cannot be too careful’; Supplementary Fig. 6C) and the sum of five questions bearing on cooperation (‘how acceptable is claiming government benefits’, ‘avoiding a fare on public transport’, ‘cheating on taxes, keeping money that you have found’, ‘failing to report damage you’ve done accidentally to a parked vehicule’; Supplementary Fig. 6D). As the Selfiecity database is constituted of pictures posted online in 2013, for each country, the most recent vague of the European or World Value Survey was taken (i.e., 2008 for Russia, 2009 for Great Britain, 2011 for the United States, 2013 for Thailand and Germany, and 2014 for Brazil). In line with our hypotheses, people located in places where interpersonal trust and cooperation are higher, had higher ratings of perceived trustworthiness in their selfies (cooperation level: *b* = 0.13 ± 0.03, *z* = 3.67, *p* < 0.001; trust level: *b* = 0.81 ± 0.23, *z* = 3.50, *p* < 0.001; Supplementary Fig. 6C, D).

### Analysis of the National portrait gallery

All the paintings of the National Portrait Gallery were downloaded in high resolution from the NPG.uk website. We limited our analysis to paintings, excluding other medium types at the National Portrait Gallery, such as drawings, sculptures and photographs. In addition, only portraits for which the image was available on the website of the National Portrait Gallery were analyzed (3152 over 3161 paintings). Information about the sitter’s age at the date of the portrait were also automatically collected. Portraits’ date were automatically coded following the method detailed in the table below (Supplementary Table 2). These values were divided by 100 for the regression analyses such that 1 time unit corresponds 100 years. All the portraits were processed using the OpenFace algorithm. The identified faces were then individually analyzed by three independent raters who were asked to evaluate the model’s goodness of fit based on the points’ position compared to the real face’s contours (coded as 0 or 1). In addition, raters had to note the gender of the sitter. The classification based on the goodness of fit was then used as weights for the analyses. Importantly, in order to ensure that the portraits accurately reflected the level of trust at the time the portrait was painted and to avoid re-interpretation of past historical figures, only portraits painted during the sitter’s lifetime were analyzed (number of analyzed portraits: *N* = 1962), however we did not control for the provider of the portraits (e.g., purchased, transferred from another museum or given by a private donator). Portraits’ dates were automatically coded following the nomenclature reported in Supplementary Table 2.

Level of affluence (countries’ GDP per capita) was provided by the Maddison Project^74^ and political democratization (Polity 2 index) was provided by the Polity IV project^75^. For the UK, these data exist from 1500 to 2000 for GDP per capita and yearly data from 1800 to 2013 for the democratization index.

In order to keep a maximal temporal resolution, missing values in the GDP per capita and Polity2 indices were completed using the closest previous value, except for the time-lag analyses in which no imputation was made. A total of 1943 data points were included in the analyses looking at the effect of GDP per capita. A total of 1115 data points were included in the analyses looking at the effect of Polity2. Paintings were analyzed using individual linear models (each painting corresponding to one data point), taking the sitter’s gender, age and level of perceived dominance as control variables. Bayes factor analyses were conducted using the BIC approximation, which approximates Bayes factors computed under the unit information prior^76^.

Finally, time-lag analyses were conducted to analyze the temporal dynamics between perceived trustworthiness, GDP per capita and democratization. To do so, data were averaged by decades and analyzed at the aggregated level. The model on perceived trustworthiness at decade *d* included the simultaneous level of perceived dominance at decade *d*, the linear effect of the time, the delayed levels of perceived trustworthiness and perceived dominance at *d-2*, and the level of GDP per capita or democratization at *d-2*. On the other hand, models of GDP per capita or democratization included the linear effect of time, the delayed level of GDP at *d-2* and the delayed levels of perceived trustworthiness and of perceived dominance at *d-2*. For each variable, the model with the delayed variable of interest (GDP per capita or democratization for the perceived trustworthiness models, and perceived trustworthiness for the models on GDP per capita and democratization) were compared with the models in which this variable was removed. Finally, in order to assess the robustness of our effects, we also tested the same models with a delay of one decade instead of two decades (Supplementary Table 3).

### Web gallery of art

Data from the Web Gallery of Art (WGA) were analyzed in a similar way as the paintings from the NPG. To better account that the portraits actually reflected the sitter’s willingness to display trustworthiness traits, paintings were geocoded using the painter’s place of activity at the time of the painting. This geo-coding resulted in 19 countries with paintings ranging from 1360 to 1918. As previously, two independent raters categorized the quality of detection of the faces and these evaluations were used as weights in the linear regression (number of analyzed portraits: *N* = 4106). As for the National Portrait Gallery, the missing levels of affluence and democratization were completed using the previous complete value. The same models as previously were used except that a random effect was included to take the localization of the paintings into account. This resulted, for the analysis of the effect of GDP per capita and democratization in two-level mixed models, taking each painting as an individual data point clustered by the country of production. Correspondingly, for time-lag analyses, we use two-level mixed models but with data aggregated by decades.

### Reporting summary

Further information on research design is available in the Nature Research Reporting Summary linked to this article.

## Supplementary information

Supplementary Information

Reporting Summary

Original reviewer TPR

## Data Availability

All data analyzed in the main text and in the supplementary materials are accessible online [https://osf.io/j68xu/?view_only=61995a283e9f4c55b43c9f31d6bd1e97] except the World Value Survey [http://www.worldvaluessurvey.org/WVSDocumentationWVL.jsp] and the European Value Survey [https://dbk.gesis.org/dbksearch/SDesc2.asp?no=4804&db=E] which are analyzed in the Selfiecity study and are freely downloadable. The source data underlying all the Figures, Tables, Supplementary Figures and Supplementary Tables are provided in the online scripts and data. A reporting summary for this Article is available as a Supplementary Information file. The images analyzed in this article are available at: Prof. Todorov avatars: http://tlab.princeton.edu; Chicago Face database [https://chicagofaces.org/default/]; Oslo Face database [https://sirileknes.com/oslo-face-database/]; Karolinska Face database [https://www.kdef.se/index.html]; FEI Face database [https://fei.edu.br/~cet/facedatabase.html]; House of Representative official portraits [https://www.house.gov/representatives]; Selfiecity [http://selfiecity.net]; National Portrait Gallery [https://www.npg.org.uk]; Web Gallery of Art [https://www.wga.hu].

## Code availability

All analyses scripts presented in the main text and in the supplementary materials are accessible online [https://osf.io/j68xu/?view_only=61995a283e9f4c55b43c9f31d6bd1e97].
