# Author Correction: Tracking historical changes in perceived trustworthiness in Western Europe using machine learning analyses of facial cues in paintings

**DOI:** 10.1038/s41467-022-31843-x

**Published:** 2022-09-14

**Authors:** Lou Safra, Coralie Chevallier, Julie Grèzes, Nicolas Baumard

**Affiliations:** 1 Laboratoire de Neurosciences Cognitives, Département d’études cognitives, ENS, PSL, Research University, INSERM Paris, France; 2 Institut Jean Nicod, Département d’études cognitives, ENS, EHESS, PSL Research University, CNRS, Paris, France; 3 Sciences Po, CEVIPOF, CNRS, Paris, France

**Keywords:** History, Human behaviour

Correction to: *Nature Communications* 10.1038/s41467-020-18566-7, published online 22 September 2020.

**Correction to Safra et al**.

The original version of the Article contained multiple inaccuracies in terminology used to describe the output of the algorithm, which were brought to our attention by readers after the publication of the study.

Specifically, the algorithm in our study was built to estimate how human raters would rate the perceived trustworthiness or perceived dominance of faces. The algorithm does not quantify the actual trustworthiness or dominance of an individual, and was not intended for this purpose, and as such, terms such as ‘trust’, ‘trustworthiness’, and ‘dominance’ were used inaccurately, instead of ‘perceived trustworthiness’, ‘perceived trust’ and ‘perceived dominance’ which more accurately reflects what was measured in the study. Similarly the terms ‘trustworthiness displays’, ‘displays of trustworthiness’ and ‘trust displays’ were used inaccurately instead of ‘ratings of perceived trustworthiness’ and ‘perceived trustworthiness’ as appropriate within the structure of the sentence.

The correct version of the Article states ‘perceived trustworthiness’ in place of ‘trustworthiness’, ‘perceived trust’ in place of ‘trust’, and ‘perceived dominance’ instead of ‘dominance’ and makes all other necessary changes to ensure clarity for the reader that the algorithm measures how human raters would perceive the trustworthiness or dominance of the sitter, and not the sitter’s actual “trustworthiness” or “dominance”.

The specific locations and wording resulting from each such change, if not already listed below, are outlined in the marked up Manuscript and Supplementary file attached to this correction published as Supplementary file 1 and 2 respectively.

All changes have been incorporated to both the PDF and HTML versions of the published article.

The original peer-review file of the article, including all reports from the reviewers and responses from the authors has been added to the supplementary information of the manuscript. The peer-review process that led to the correction is published as Supplementary 3 to this correction.

**Title**

The original version of the title omitted information on the region of the sample of paintings being studied. The full correct version of the title states ‘Tracking historical changes in perceived trustworthiness in Western Europe using machine learning analyses of facial cues in paintings’ in place of ‘Tracking historical changes in trustworthiness using machine learning analyses of facial cues in paintings’

**Abstract**

The original version of this Article contained errors in the third sentence of the abstract, which did not accurately reflect the use of the algorithm in the study and incorrectly referred to ‘trustworthiness’ rather than ‘perceived trustworthiness’. The correct sentence states ‘Building on recent advances in social cognition, we design an algorithm to automatically estimate ratings of perceived trustworthiness from specific facial cues (such as muscle contractions associated with smiling) detected in European portraits in large historical databases’ instead of ‘Building on recent advances in social cognition, we design an algorithm to automatically generate trustworthiness evaluations for the facial action units (smile, eye brows, etc.) of European portraits in large historical databases.’

The original version of this Abstract omitted a clarifying sentence. The correct version includes the following sentence ‘We used this measure as a proxy of social trust in history.’

The original version of this Article contained an error in the final sentence of the second paragraph of the abstract, which omitted references to existing references 21, 22, 24–26. These have been added at the following sentence ‘Therefore, we chose to assess the validity and generalizability of our model independently of idiosyncratic biases of participants by relying on well-known effects in the literature, i.e., the effect of emotion, age, gender, and head orientation on facial evaluations.^21, 22, 24–26^’.

The original version of this Article contained errors in the second last sentence of the abstract, which referred to measures not tested in this study. The correct sentence states ‘Our results show that estimated levels of perceived trustworthiness in portraits increased over the period 1500–2000.’ instead of ‘Our results show that trustworthiness in portraits increased over the period 1500–2000 paralleling the decline of interpersonal violence and the rise of democratic values observed in Western Europe.’

**Introduction**

The original version of this Article contained an error in the fourth sentence of the second paragraph of the Introduction in which the region of the sample of portraits was omitted. The correct sentence states ‘More precisely, we apply recent machine-learning methods to extract quantitative information about the evolution of social cues contained in Western European portraits.’ instead of ‘More precisely, we apply recent machine-learning methods to extract quantitative information about the evolution of social cues contained in portraits.’

We also added two clarifying sentences from the second paragraph of the introduction which read ‘Crucially, this algorithm does not provide information on a person’s face but rather on the way this face is likely to be perceived by others, based on a specific image. Indeed, first impressions from faces are highly sensitive to factors such as variations in lighting and pose.’

We added a clarifying sentence at the end of the Introduction which reads ‘In this article, all occurrences of the words ‘trustworthiness’ and ‘dominance’ refer to subjective perceptions of trustworthiness and dominance from faces and not to individuals’ actual level of trustworthiness or dominance.’

The original version of this Article contained an error in the fifth sentence of the second paragraph of the introduction which omitted to clarify how the model was built, and incorrectly referred to ‘trustworthiness ratings on portraits’ rather than ‘ratings of perceived trustworthiness’. The correct sentence states ‘The algorithm is built on models of human perception of faces to generate automatic human-like ratings of perceived trustworthiness based on the muscle contractions (facial action units) detected in facial displays in portraits using the open software OpenFace.’ instead of ‘The algorithm generates automatic human-like trustworthiness ratings on portraits based on the muscle contractions (facial action units) detected in facial displays using the open software OpenFace’.

The original version of this Article contained an error in the seventh sentence of the second paragraph of the abstract which incorrectly described the avatars used for training the algorithm as being ‘controlled for trustworthiness’ rather than ‘generated to display varying levels of perceived trustworthiness’. The correct sentence states ‘This algorithm was trained on avatars generated to display varying levels of perceived trustworthiness and optimized using a random forest procedure (see Supplementary Methods for more details).’ Instead of ‘This algorithm was trained on avatars controlled for trustworthiness and optimized using a random forest procedure (see Supplementary Methods for more details).’

**Results**

The fifth sentence of the first paragraph of the results section omitted to note the small size in the increase of perceived trustworthiness. The correct sentence states ‘Although the increase of perceived trustworthiness is small, these results are consistent with more qualitative works documenting a so-called ‘Smile Revolution’^27^ and a rise of prosocial displays in paintings and in novels^28^.’ instead of ‘Overall, these results are consistent with more qualitative works documenting a so-called ‘Smile Revolution’^25^ and a rise of prosocial displays in paintings and in novels^26^.’

We now include in the second section of the Results a clarifying paragraph to discuss effect sizes and cite related work. The following text has been added along with references 46–51 to the Reference list.

‘These results provide evidence in favor of the association between economic wealth and social trust at the society level. However, due to the small effect sizes and the limitations of the historical economic indicators^46,47^, as well as to the fact that GDP per capita is only a partial measure of wealth (which does not account, for example, for inequalities in wealth distribution^48^), we replicated our analyses with an alternative variable known to be associated with countries’ wealth: the number of book titles per capita. Indeed, although the number of book titles per capita is thought to be linked to human development variables, it has also been shown to be associated with national income^48–51^. Supporting the analyses conducted with GDP per capita, we found a significant positive association between the number of book titles per capita and the level of perceived trustworthiness in the portraits of the National Portrait Gallery (affluence only model: *b* = 0.35 ± 0.06, *z* = 6.15, *p* < 0.001; model controlling for time: *b* = 0.21 ± .06, *z* = 3.45, *p* = 0.001) and of the Web Gallery of Art, although not robust to the inclusion of time in this latter case (affluence only model: *b* = 0.29 ± 0.10, *z* = 2.77, *p* = 0.006; model controlling for time: *b* = 0.14 ± 0.11, *z* = 1.26, *p* = 0.208).’

The full details of the added references are as follows.

46. Bolt, J., Inklaar, R., De Jong, H., & Van Zanden, J. L. (2018). Rebasing ‘Maddison’: new income comparisons and the shape of long-run economic development. *GGDC Res. Memorandum*, **174**, 1–67.

47. Broadberry, S. Campbell, B. M., Klein, A., Overton, M., & van Leeuwen, B. *British Economic Growth*, 1270–1870 (Cambridge University Press, 2015).

48. Alfani, G., & Ammannati, F. (2017). Long‐term trends in economic inequality: the case of the Florentine state, *c*. 1300–1800. *Econ. Hist. Rev.*, **70**, 1072–1102.

49. van Leeuwen, B., Plopeanu, A.-P. & Foldvari, P. Publishing ideas: the factors determining the number of book titles. *Acta Oecon.*, **68**, 443–466 (2018).

50. Baten, J. & van Zanden, J. L. Book Production and the Onset of Modern Economic Growth. 24 *J. Econ. Growth*, **13**, 217–235 (2008).

51. Buringh, E. & Van Zanden, J. L. Charting the ‘Rise of the West’: manuscripts and printed books in Europe, a long-term perspective from the sixth through eighteenth centuries. *J. Econ. Hist.* **69**, 409–445 (2009).

**Discussion**

The original version of the Article omitted clarifying information from the discussion. The added section reads as follows: ‘The algorithm was built to estimate how human raters would rate the perceived trustworthiness of faces. It can be used in scientific research for this purpose. The algorithm does not quantify the actual trustworthiness of an individual, and was not intended for this purpose.’

The original version of the Article omitted a section to discuss limitations of the study in the Discussion. The added section reads as follows:

‘At this point, it is important to note the small correlation between the perceived trustworthiness ratings provided by human raters and those retrieved by our algorithm. However, this small effect size is to be expected. First, the avatars on which the algorithm was trained did not represent the texture of the faces, even though this information may influence human raters’ evaluations. Similarly, the avatars are bold and our algorithm is thus blind to haircut, even though these cues are known to influence first impressions from faces (see e.g.,^21^). Finally, our algorithm was trained to generate ratings of perceived trustworthiness based on the facial features that represent the shared component of first impressions from faces. Indeed, individuals rely on both shared and idiosyncratic features when forming a first impression on a new face, and our algorithm was designed to produce scores only based on the former.

Finally, several limitations are to be noted. First, one cannot assume that the evolution of perceived trustworthiness depicted in this study extends to the larger population of the period. The phenomenon described in this article might, for instance, be limited to the relatively elite, wealthy population represented in the portraits. In line with this possibility, there is evidence that social attitudes can vary with socioeconomic status^55–58^. Second, our study is based on the assumption that facial cues that are used as cues to assess perceived trustworthiness are shared across time. Although recent evidence^59–61^ points towards such a stability, further work is needed to fully test this assumption. Third, times series of GDP per capita and living standards are only estimates, and their precision may fluctuate throughout the studied time period and fail to fully capture the evolution of living standards and inequalities^46–48^.’

**Methods**

The fourth section of the Methods section omitted a clarifying sentence. The added sentence reads ‘We limited our analysis to paintings, excluding other medium types at the National Portrait Gallery, such as drawings, sculptures and photographs. In addition, only portraits for which the image was available on the website of the National Portrait Gallery were analyzed (3152 over 3161 paintings).’

The ninth sentence of the first paragraph of the fourth section of the Methods section contained an error in which a clarifying phrase was omitted ‘however we did not control for the provider of the portraits (e.g., purchased, transferred from another museum or given by a private donator).’ The correct sentence reads ‘Importantly, in order to ensure that the portraits accurately reflected the level of trust at the time the portrait was painted and to avoid re-interpretation of past historical figures, only portraits painted during the sitter’s lifetime were analyzed (number of analyzed portraits: *N* = 1962), however we did not control for the provider of the portraits (e.g., purchased, transferred from another museum or given by a private donator).’ instead of ‘Importantly, in order to ensure that the portraits accurately reflected the level of trust at the time the portrait was painted and to avoid re-interpretation of past historical figures, only portraits painted during the sitter’s lifetime were analyzed (number of analyzed portraits: *N* = 1962).’

**Figure legends**

The Figure 1 legend contained an error in part B in which ‘displays of ‘trustworthiness’ was used instead of ‘perceived trustworthiness’, dominance was used instead of ‘perceived dominance’ and omitted a clarifying phrase ‘for representation purposes, in this Figure, evaluation of perceived trustworthiness value was fitted by a local polynomial regression with a span of 0.75 and’.

The correct sentence reads ‘B. Evolution of ratings of perceived trustworthiness in the National Portrait Gallery (modeled for representation purposes, in this Figure, evaluation of perceived trustworthiness value was fitted by a local polynomial regression with a span of 0.75 and adjusted for perceived dominance) and GDP per capita in England. (log-transformed for representation purposes).’ instead of ‘Evolution of displays of trustworthiness in the National Portrait Gallery (modeled trustworthiness value adjusted for dominance) and GDP per capita in England.’

**Acknowledgements**

The original version of the manuscript inadvertently named reviewers in the Acknowledgement section. These have been removed.

**Supplementary Information**

The original version of the Article omitted Supplementary Table 5. The HTML has been updated to include a corrected version of the Supplementary Information which includes the following table and legend.

**Table:** 

	Affluence only		Time + Affluence		Armed conflict only		Time + Armed conflict	
	National portraits gallery	Web gallery of art	National portraits gallery	Web gallery of art	National portraits gallery	Web gallery of art	National portraits gallery	Web gallery of art
year			0.11 ± 0.02 *z* = 5.46 *p* < 0.001	0.05 ± 0.01 *z* = 3.54 *p* < 0.001			0.14 ± 0.02 *z* = 7.55 *p*<0.001	0.05 ± 0.01 *z* = 4.13 *p* < 0.001
Number of book titles per capita	0.35 ± 0.06 *z* = 6.15 *p* <0.001	0.29 ± 0.10 *z* = 2.77 *p* = 0.006	0.21 ± 0.06 *z* = 3.45 *p* = 0.001	0.14 ± 0.11 *z* = 1.26 *p* = 0.208				
Presence of an armed conflict					0.01 ± 0.05 *z* = 0.30 *p* > 0.250	0.00 ± 0.03 *z* =−0.01 *p* > 0.250	0.05 ± 0.05 *z* = 1.05 *p* > 0.250	−0.01 ± 0.03 *z* = −0.39 *p* > 0.250
Control variables								
Perceived dominance	−0.78 ± 0.02 *z* = −40.10 *p* < 0.001	−0.75 ± 0.02 *z* = −54.29 *p* < 0.001	−0.79 ± 0.02 *z* = −40.85 *p* < 0.001	−0.74 ± 0.01 *z* = −54.13 *p* < 0.001	−0.78 ± 0.02 *z* = −39.79 *p* < 0.001	−0.74 ± 0.01 *z* = −54.85 *p* < 0.001	−0.79 ± 0.02 *z*=−40.74 *p* < 0.001	−0.74 ± 0.02 *z*=−54.86 *p* < 0.001
Gender	−0.31 ± 0.06 *z* = −5.27 *p* <0.001	−0.33 ± 0.03 *z* = −11.13 *p* < 0.001	−0.29 ± 0.06 *z* = −5.09 *p* < 0.001	−0.32 ± 0.03 *z* = −10.52 *p* < 0.001	−0.37 ± 0.06 *z* = −6.41 *p* < 0.001	−0.33 ± 0.03 *z* = −11.51 *p* < 0.001	−0.33 ± 0.06 *z* = −5.68 *p*<0.001	−0.31 ± 0.03 *z* = − 10.49 *p* < 0.001
Age	−0.00 ± 0.00 *z* = −1.35 *p* = 0.178		−0.00 ± 0.00 *z* = −2.49 *p* = 0.013		0.00 ± 0.00 *z* = 0.21 *p* > 0.250		−0.00 ± 0.00 *z* = −2.01 *p* = 0.044	
Sample								
N	1962	3801	1962	3801	1962	3927	1962	3927

**Supplementary Table 5** Replication analyses on perceived trustworthiness in the National Portrait Gallery and the Web Gallery of Art using the Number of book titles per capital as a proxy of affluence as well as the presence of armed conflict as indicator of periods of war and social unrest.

The first line corresponds to the regression coefficient with their associated standard error to the mean (mean ± s.e.m.). Results in bold corresponds to statistically significant effects of the variables of interest. The upper part of the table presents the effects of the variables of interest (time, affluence and democratization), while the lower part presents the effects of the control variables (perceived dominance, gender and age). All the tests are two-sided. Following APA’s recommendations, exact *p*-values are provided for p-values between 0.001 and 0.250. Source data are provided as raw data and scripts on the online depository.

## Supplementary information

Supplementary Information 1

Supplementary Information 2

Supplementary Information 3

Peer review file

Updated supplementary information

