# Supplementary material for: Tracking historical changes in perceived trustworthiness in Western Europe using machine learning analyses of facial cues in paintings
Source: Nat Commun. 2020 Sep 22;11:4728. doi: 10.1038/s41467-020-18566-7 (PMC7508927; doi:10.1038/s41467-020-18566-7)
Supplement: Supplementary file 1 — Supplementary Information [file 41467_2020_18566_MOESM1_ESM.pdf]

Supplementary Information

Tracking historical changes in perceived trustworthiness in Western Europe using machine learning analyses of facial cues in paintings.

Correspondence to:
Nicolas Baumard (nicolas.baumard@ens.fr) & Lou Safra (lou.safra@sciencespo.fr)

This PDF file includes:

Supplementary Methods
Supplementary Figures. 1 to 8
Supplementary Tables 1 to 4

Supplementary Methods

In order to quantify perceived trustworthiness displays in historical paintings, we developed an algorithm automatically estimating perceived trustworthiness from faces. Our algorithm also extracted perceived dominance since perceived dominance has been shown to be, together with perceived trustworthiness, one of the main dimensions of social perception [1]. Crucially, although dominance displays carry signals of power that are distinct from the cooperation-related signals associated with trustworthiness displays, perceived dominance and perceived trustworthiness are correlated [1]. This correlation entails that it is of paramount importance to control for perceived dominance when analyzing perceived trustworthiness. This type of analysis, studying together distinct but related social signals, has already been shown to be particularly promising in the emotion domain by revealing the importance of taking into account the existence of compound emotions [2].

Construction and validation of an algorithm for modeling perceived trustworthiness and perceived dominance evaluations

We built a model that automatically extracts evaluations of perceived trustworthiness and perceived dominance from the all the facial action units detected by the OpenFace algorithm (i.e., both dichotomous and continuous estimations; OpenFace version 1.01 using OpenCV 3.3.0 [3]). To do so, we extracted the facial action units of five sets of avatars previously generated with Facegen and controlled for perceived dominance, for perceived trustworthiness or for both (Supplementary Figure 1) [4]. Each avatar is generated from an initial face and manipulated to either express a specific level of perceived dominance, perceived trustworthiness or both based on the model developed by Oosterhof & Todorov [1]. These avatar faces have been shown to successfully elicit ratings of perceived dominance and perceived trustworthiness in participants [4–6]. Thus, compared to participants' ratings on photographs that may be sensitive to the participants characteristics and to experimental protocol factors (such as the type of scale used to give the ratings), using avatars allow us to have well-validated sets of faces to train our model. These sets of avatars correspond to all the existing and available validated avatars controlled for perceived trustworthiness or perceived dominance and generated by Facegen.

More precisely, one set of avatars was generated from one single face and manipulated for both perceived dominance and perceived trustworthiness ($N = 49$; 7 levels of perceived dominance and 7 levels of perceived trustworthiness, each of the 7 levels corresponds to a standard deviation in Oosterhof and Todorov's [1] model ranging between -3 to +3 SD; set 1). Two other sets of faces correspond to 25 maximally distinct faces manipulated either on perceived trustworthiness only ($N = 175$; 7 different levels of perceived trustworthiness; set 2) or perceived dominance only ($N = 175$; 7 different levels of perceived dominance; set 3). Finally, the two last sets are composed of 25 Caucasian faces manipulated to present the same 7 levels of perceived trustworthiness ($N = 175$; set 4) or of perceived dominance ($N = 175$; set 5). Thus, three sets of avatars were used to build the model automatically extracting perceived trustworthiness levels (sets 1, 2 and 4) and three were used to build the model automatically extracting perceived dominance levels (sets 1, 3 and 5).

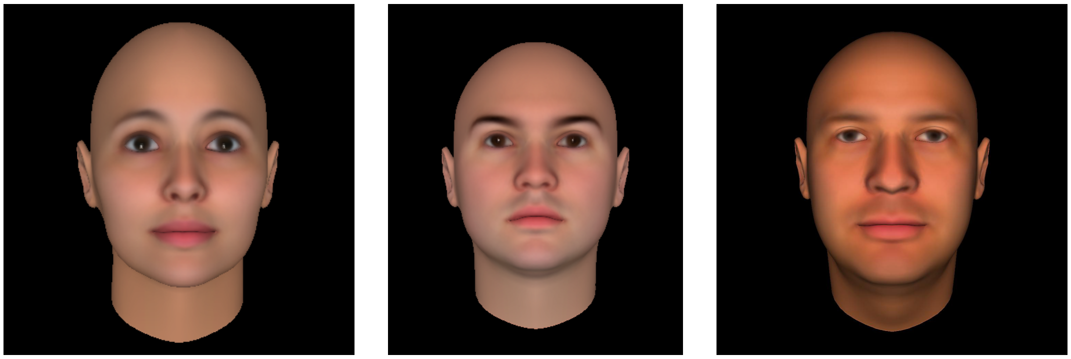

Supplementary Figure 1 Sample of the avatar faces used for the algorithm optimization. **Left**. Face for the set of avatars controlled for perceived dominance and perceived trustworthiness; **Middle**. Example of a face for one of the sets of avatars controlled for perceived dominance only and one of the sets controlled for perceived trustworthiness only; **Right**. Example of a face of the 'Maximally distinct faces' for the other set of avatar controlled for perceived dominance only and for the other set of avatars controlled for perceived trustworthiness only. These three images were created by Prof. Alexander Todorov's team and is shared under license CC BY.

Because all our avatars were generated using the same models for perceived trustworthiness and perceived dominance, actions units with a variance inferior to 0.01 were discarded as not informative enough regarding cues of perceived trustworthiness and perceived dominance. The reason was that they were either too low in frequency or too low in intensity (ten action units discarded over thirty-three in both the perceived trustworthiness and perceived dominance avatar sets).

	SVM linear	SVM radial	Random forest	Linear model
Hyperparameters	*Cost (C)*	Cost (C) & sigma	*mtry*	ø
	Perceived trustworthiness			
Mean absolute error	0.88 ± 0.02	**0.87 ± 0.02**	**0.82 ± 0.01**	0.87 ± 0.01
Root mean squared deviation	1.10 ± 0.02	**1.05 ± 0.02**	**0.99 ± 0.01**	1.06 ± 0.02
R squared	0.71 ± 0.01	**0.74 ± 0.01**	**0.78 ± 0.01**	0.72 ± 0.01
	Perceived dominance			
Mean absolute error	0.92 ± 0.02	**0.79 ± 0.02**	**0.80 ± 0.01**	0.90 ± 0.02
Root mean squared deviation	1.14 ± 0.02	**0.99 ± 0.02**	**0.98 ± 0.02**	1.11 ± 0.02
R squared	0.68 ± 0.01	**0.76 ± 0.01**	**0.77 ± 0.01**	0.70 ± 0.01

Supplementary Table 1. Model selection for extracting evaluations of perceived trustworthiness and perceived dominance. Three indices of fit were computed, two which minimization indicates a better fit (mean absolute error and root mean squared deviation) and one which maximization indicates a better fit (R squared). The random forest was outperforming the linear model and the linear support vector model in the three indices of fit tested: mean absolute error, root mean squared deviation and r-squared. The random forest model was better than the radial support vector model for the perceived trustworthiness model and similar to the radials support vector model for the perceived dominance model. Values are presented as mean ± standard error to the mean. Source data are provided as raw data and scripts on the online depository.

Based on our validation results on the avatar faces, we then trained the perceived trustworthiness and perceived dominance models with the same hyperparameters on the entire avatar dataset in order to increase the accuracy of our estimates and tested this model on an independent set of photographs. This method differs from the classical train-test split used in machine learning which was not applicable given that each avatar of our dataset presented unique features in terms of luminance, texture and face shape which was important to increase the accuracy of our algorithms. However, our procedure is a highly conservative test of the validity of our models as the test set is completely different and independent of the training set. This conservative method for assessing the validity of the algorithms is particularly critical in the present study as our goal is to generalize the estimated perceived trustworthiness and perceived dominance evaluations to historical portraits, a completely different set of images than those classically used in social cognition research.

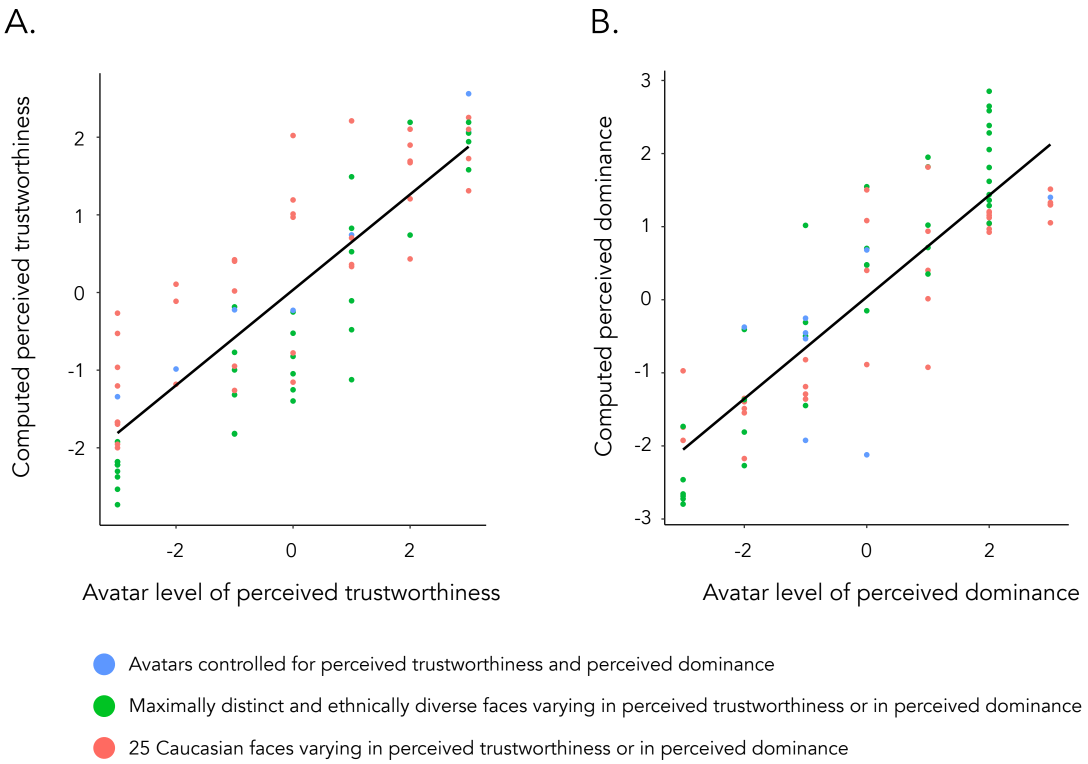

Supplementary Figure 2 Correlation between the avatars' actual level of perceived trustworthiness and perceived dominance in the test set and the computed perceived trustworthiness (A; Pearson correlation: $r = .85$, $t(75) = 14.17$, $p < .001$) and perceived dominance (B; Pearson correlation: $r = .86$, $t(75) = 14.72$, $p < .001$) based on the model optimized on the training set only. Source data are provided as raw data and scripts on the online depository.

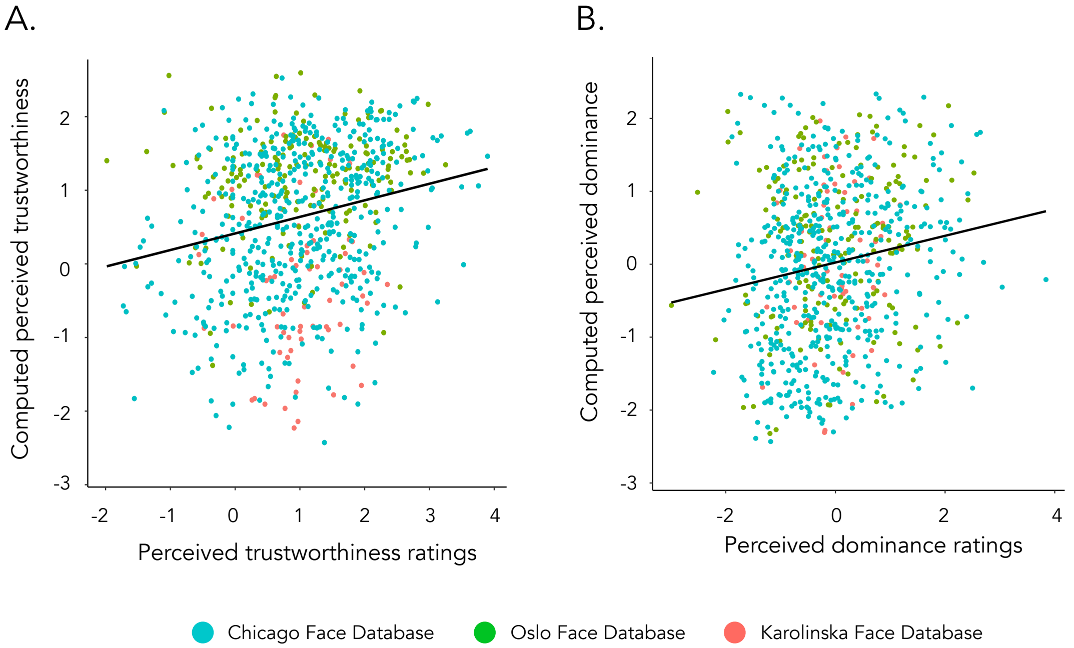

Supplementary Figure 3 Correlation between participants' ratings of perceived trustworthiness and dominance displays in the three databases providing subjective ratings of perceived trustworthiness and perceived dominance (the Chicago Face Database, the Oslo Face Database and the Karolinska Face Database) and the retrieved perceived trustworthiness (**A**, Pearson correlation: r = .22, $t(768)$ = 6.19, p < .001) and retrieved perceived dominance (**B**, Pearson correlation: r = .16, $t(769)$ = 4.54, p < .001) levels estimated using the Facial Action Units detected by Open Face and our random-forest model. Source data are provided as raw data and scripts on the online depository.

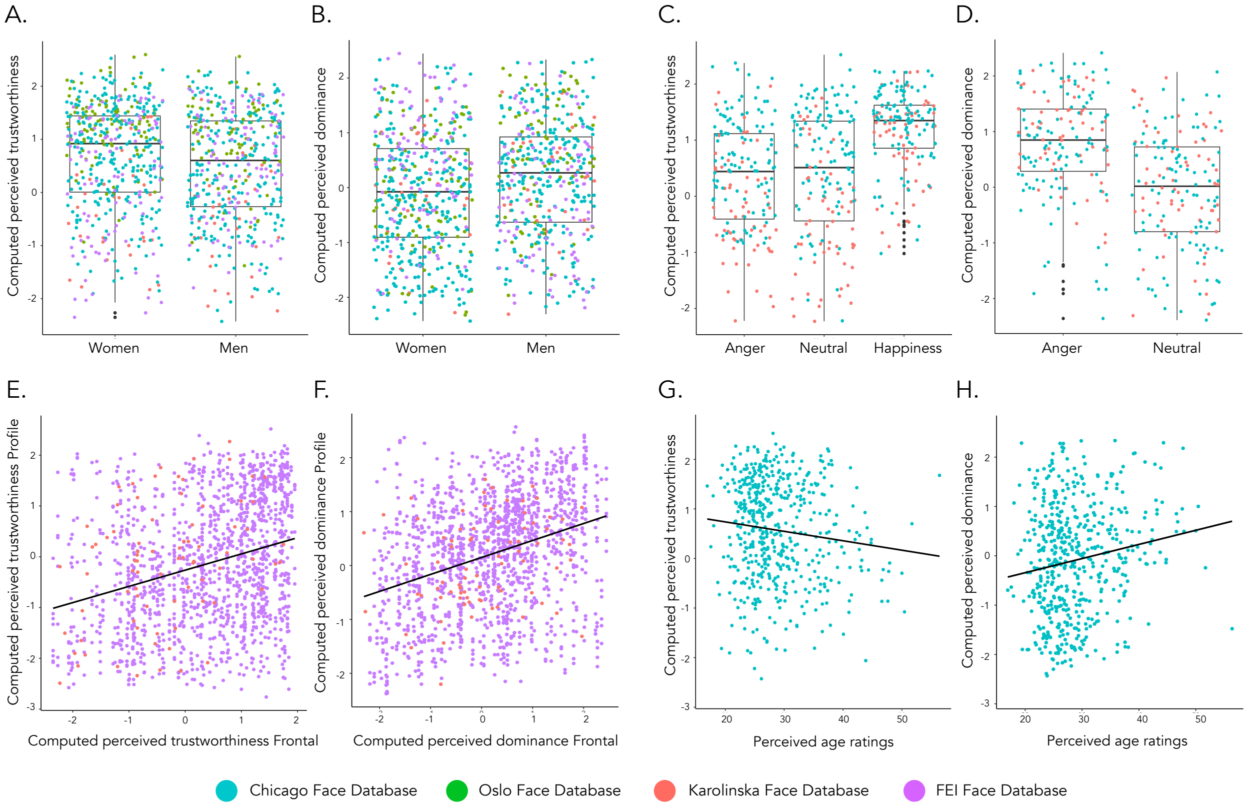

Supplementary Figure 4 Recovery of classical effects of gender (**A-B**, Student t-test: perceived trustworthiness $t(972) = 2.67$, $p = .008$; perceived dominance: $t(972) = -3.63$, $p < .001$), emotion (**C-D**, two-level linear regression: perceived trustworthiness: $t(167) = 10.64$, $p < .001$; perceived dominance: $t(167) = 9.42$, $p < .001$), head orientation (**E-F**; Pearson correlations: perceived trustworthiness $r = .29$, $t(1500) = 11.51$, $p < .001$; perceived dominance: $r = 0.34$, $t(1500) = 13.79$, $p < .001$) and age (**G-H**, Pearson correlations: perceived trustworthiness: $r = -.12$, $t(518) = -2.68$, $p = .008$; perceived dominance: $r = 0.16$, $t(518) = 3.70$, $p < .001$) in the perceived trustworthiness and perceived dominance estimates computed using our random forest algorithm. In the boxplots (**A-D**), the centre line corresponds to the median, the lower and upper bounds of the box to the 25th and 75th percentiles and the whiskers to the largest and lowest values in a limit of 1.5 times the inter-quartile range from the box bounds. Source data are provided as raw data and scripts on the online depository.

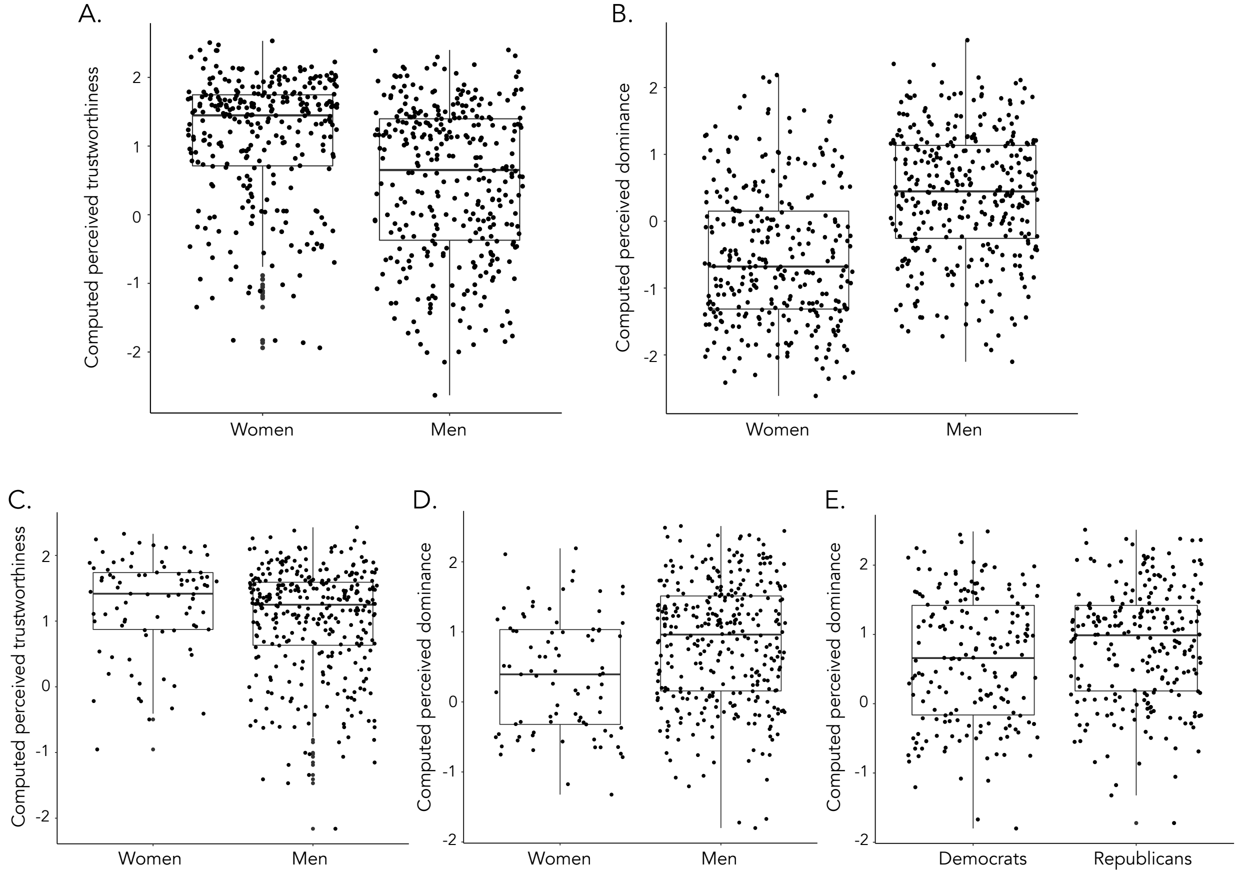

Supplementary Figure 5 Results on natural images

A-B Recovery of the classical effects of gender in Google Image portraits of 'Women' (*N* = 304 images) and 'Men' (*N* = 330 images); **C-E** Recovery of the classical gender (**C-D**) and party (**E**) effects on the portraits of the House of the Representatives (women : *N* = 85 images ; men : *N* = 334 images ; democrats : *N* = 182 images ; republicans : *N* = 237 images). The centre line corresponds to the median, the lower and upper bounds of the box to the 25th and 75th percentiles and the whiskers to the largest and lowest values in a limit of 1.5 times the inter-quartile range from the box bounds. Source data are provided as raw data and scripts on the online depository.

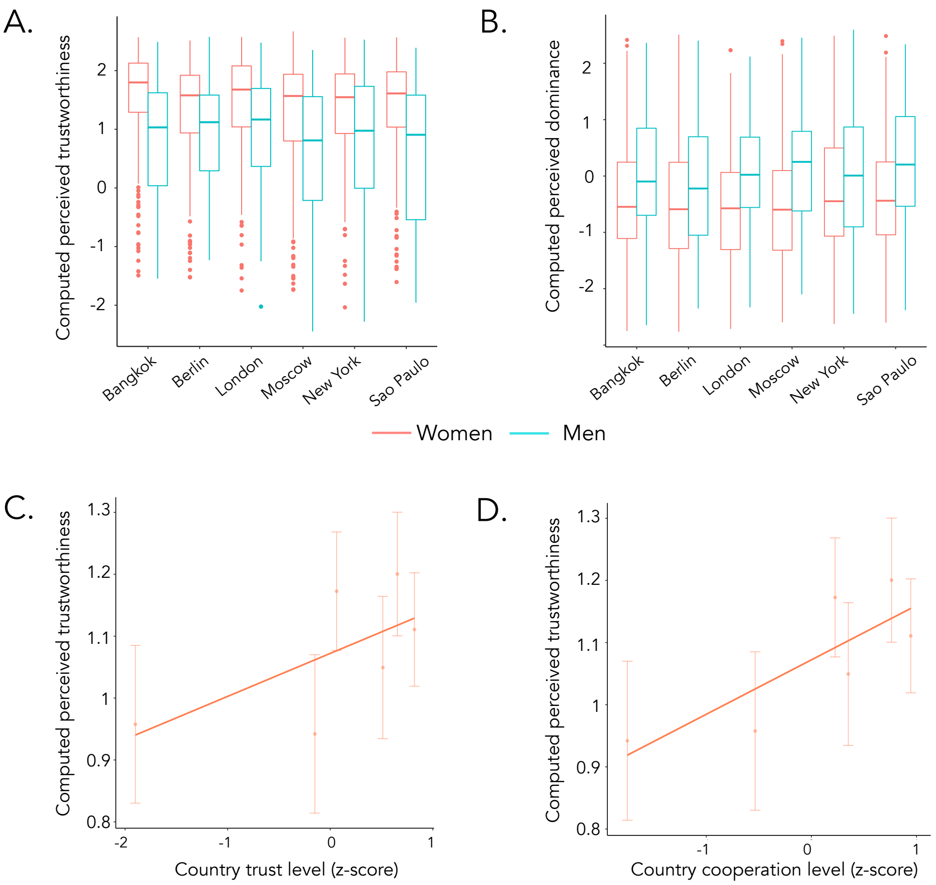

Supplementary Figure 6 Results on the Selfiecity Database
A-B Recovery of the classical effects of gender (Bangkok : $N = 247$ selfies of women, $N = 169$ selfies of men ; Berlin : $N = 239$ selfies of women, $N = 163$ selfies of men ; London : $N = 217$ selfies of women, $N = 134$ selfies of men ; Moscow : $N = 338$ selfies of women, $N = 82$ selfies of men ; New York : $N = 210$ selfies of women, $N = 127$ selfies of men ; Sao Paulo : $N = 231$ selfies of women, $N = 120$ selfies of men). The centre line corresponds to the median, the lower and upper bounds of the box to the 25th and 75th percentiles and the whiskers to the largest and lowest values in a limit of 1.5 times the inter-quartile range from the box bounds.; **C-D** Significant association between the country's level of interpersonal trust (**C;**) and cooperation (**D**) and the mean perceived trustworthiness estimated on the pictures of the Selfiecity database averaged between portraits of women and men, the red line corresponds to the effect computed in the regression controlling for the gender of the sitters (interpersonal trust: $b = 0.81 \pm 0.23$, $z = 3.50$, $p < .001$; cooperation: $b = 0.13 \pm 0.03$, $z = 3.67$, $p < .001$). Data are represented as mean values and error bars correspond to standard errors to the mean (Bangkok : $N = 416$ selfies ; Berlin : $N = 402$ selfies ; London : $N = 351$ selfies ; Moscow : $N = 420$ selfies ; New York : $N = 337$ selfies ; Sao Paulo : $N = 351$ selfies). Source data are provided as raw data and scripts on the online depository.

Analysis of the National Portrait Gallery and the Web Gallery of Art

Text	Code	Example
Century	Century + 50	16th century = 1550
Late century	Centruy + 90	Late 16th century = 1590
Early century	Century + 10	Early 16th century = 1510
Half of century	Century + 50	Half of 16th century = 1550
Decade+s	Decade	1650s = 1655
Around/about/perhaps/probably/circa/after + Date	Date	Circa 1655 = 1655
Date 1 – Date 2	Rounded mean of Date 1 and Date 2	1650-1655 = 1652

Supplementary Table 2 – Coding of the date of the portraits

The information about the sitters' gender and age allowed us to replicate the classic findings that older sitters appear more dominant and less trustworthy than younger sitters and that female sitters appear more trustworthy and less dominant than male sitters (perceived trustworthiness: gender effect: $t(1960) = 9.69$, $p < .001$; age effect: $t(1960) = -6.63$, $p < .001$; perceived dominance: gender effect: $t(1960) = 7.24$, $p < .001$; age effect: $t(1960) = -9.12$, $p < .001$; Supplementary Figure 7). As for the NPG, we accurately recovered the gender effect on perceived trustworthiness and d perceived dominance on the portraits of the Web Gallery of Art (perceived trustworthiness: $z = 17.70$, $p < .001$; perceived dominance: $z = -13.35$, $p < .001$; Supplementary Figure 8).

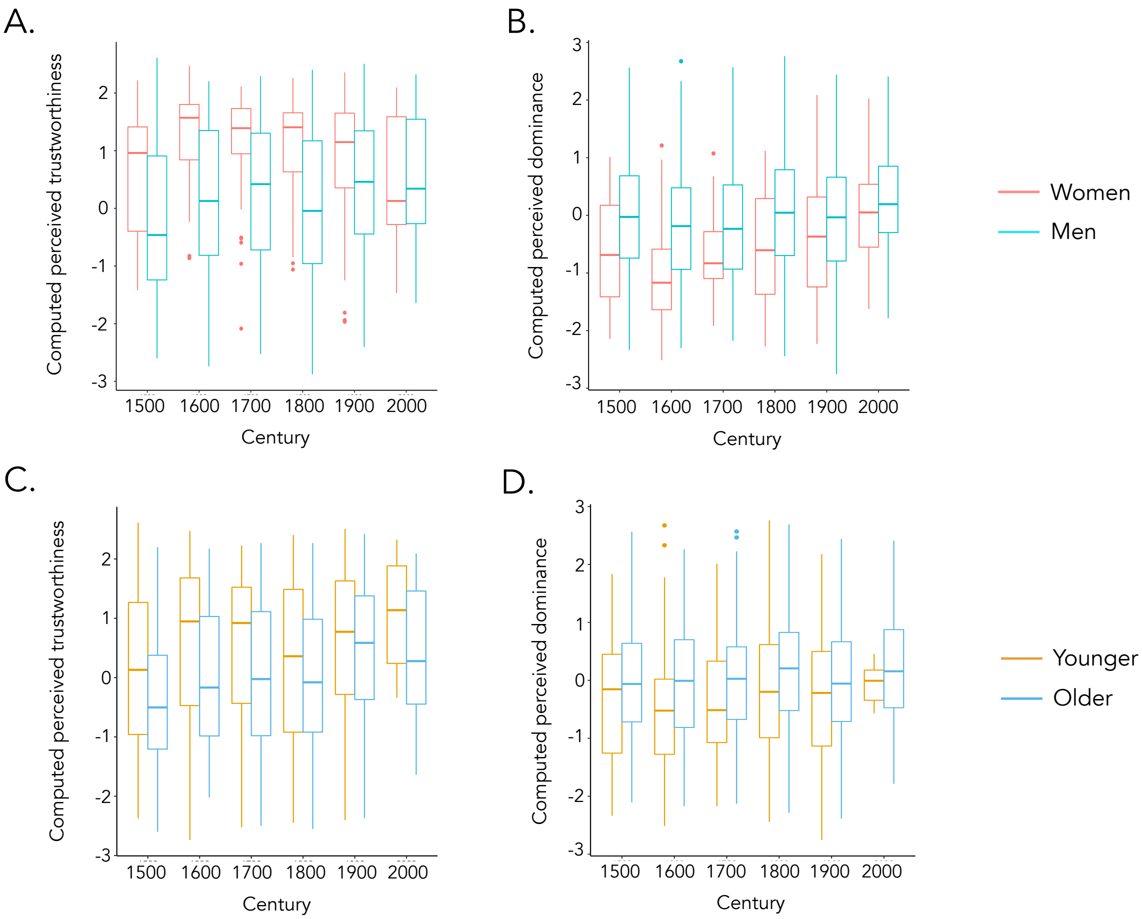

Supplementary Figure 7 Recovery of the gender (**A-B**) (1500 : $N = 23$ portraits of women, $N = 68$ portraits of men ; 1600 : $N = 50$ portraits of women, $N = 236$ portraits of men ; 1700 : $N = 53$ portraits of women, $N = 432$ portraits of men ; 1800 : $N = 44$ portraits of women, $N = 609$ portraits of men ; 1900 : $N = 98$ portraits of women, $N = 351$ portraits of men ; 2000 : $N = 19$ portraits of women, $N = 42$ portraits of men) and age (**C-D**) effects in the National Portrait Gallery database over the centuries (the 'Younger' category is defined as sitters being under 48 year old; 1500 : $N = 61$ portraits of younger sitters, $N = 30$ portraits of older sitters; 1600 : $N = 188$ portraits of younger sitters, $N = 96$ portraits of younger sitters ; 1700 : $N = 280$ portraits of younger sitters, $N = 194$ portraits of older sitters; 1800 : $N = 273$ portraits of younger sitters, $N = 345$ portraits of older sitters; 1900 : $N = 187$ portraits of younger sitters, $N = 249$ portraits of older sitters; 2000 : $N = 8$ portraits of younger sitters, $N = 53$ portraits of older sitters). The centre line corresponds to the median, the lower and upper bounds of the box to the 25th and 75th percentiles and the whiskers to the largest and lowest values in a limit of 1.5 times the inter-quartile range from the box bounds. Source data are provided as raw data and scripts on the online depository.

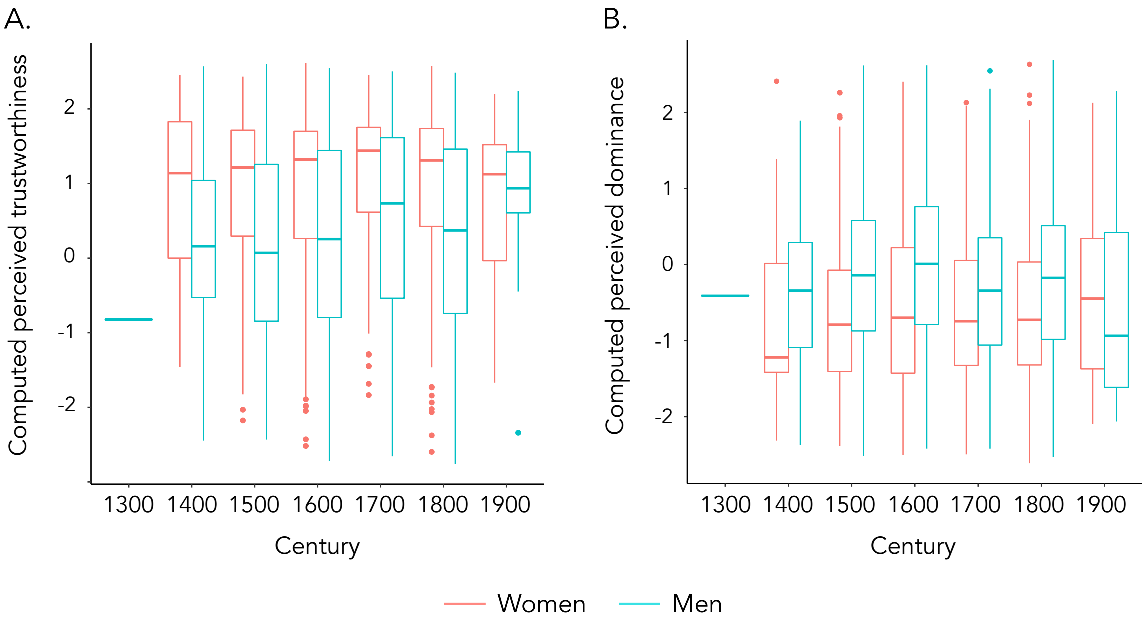

Supplementary Figure 8 Recovery of the gender effects in the Web Gallery of Art (1300 : *N* = 1 portrait of man ; 1400 : *N* = 137 portraits of men, *N* = 41 portraits of women ; 1500 : *N* = 696 portraits of men, *N* = 291 portraits of women ; 1600 : *N* = 963 portraits of men, *N* = 509 portraits of women ; 1700 : *N* = 418 portraits of men, *N* = 350 portraits of women ; 1800 : *N* = 349 portraits of men, *N* = 307 portraits of women ; 1900 : *N* = 22 portraits of men, *N* = 22 portraits of women) for perceived trustworthiness (**A**) and perceived dominance (**B**). The centre line corresponds to the median, the lower and upper bounds of the box to the 25th and 75th percentiles and the whiskers to the largest and lowest values in a limit of 1.5 times the inter-quartile range from the box bounds. Source data are provided as raw data and scripts on the online depository.

Dependent variable	Perceived Trustworthiness		GDP per capita	Democratization
Independent variable of interest	GDP per capita	Democratization	Perceived Trustworthiness	Perceived Trustworthiness
Delay Two decades				
Model comparison	F(40,1) = 12.38<br>p = .001	F(15,1) = 0.11<br>p > .250	F(41,1) = 0.76<br>p > .250	F(16,1) = 6.54<br>p = .022
Effect	b = 0.04 ± 0.01<br>t(40) = 3.52<br>p = .001	b = -0.01 ± 0.03<br>t(14) = -0.33<br>p > .250	b = 0.59 ± 0.68<br>t(41) = 0.87<br>p > 250	b = -5.82 ± 2.27<br>t(15) = -2.56<br>p = .022
Delay One decade				
Model comparison	F(41,1) = 11.40<br>p = .002	F(16,1) = 1.11<br>p > .250	F(42,1) = 0.01<br>p > .250	F(17,1) = 5.26<br>p = .036
Effect	b = 0.03 ± 0.01<br>t(40) = 3.38<br>p = .002	b = -0.02 ± 0.02<br>t(15) = -1.05<br>p > .250	b = -0.05 ± 0.66<br>t(41) = -0.08<br>p > .250	b = -4.19 ± 1.82<br>t(16) = 0.64<br>p > .250

Supplementary Table 3 Temporal dynamics of perceived trustworthiness, GDP per capita and democratization in the paintings of the National Portrait Gallery. Model comparison corresponds to the comparison of the model that included the delayed variable of interest with the model in which this variable was excluded. Effect corresponds to

the estimation of the regression coefficient of the delayed variable of interest. All the tests are two-sided. Following APA's recommendations, exact p-values are provided for p-s between .001 and .250. Source data are provided as raw data and scripts on the online depository.

Dependent variable	Perceived Trustworthiness		GDP per capita	Democratization
Independent variable of interest	GDP per capita	Democratization	Perceived Trustworthiness	Perceived Trustworthiness
Delay One decade				
Model comparison	X(1) = 4.00<br>p = .046	X(1) = 0.01<br>p > .250	X(1) = 2.48<br>p = .115	X(1) = 0.65<br>p > .250
Effect	b = 0.12 ± 0.05<br>z = 2.61<br>p = .009	b = 0.00 ± 0.01<br>z = -0.11<br>p > .250	b = -0.03 ± 0.02<br>z = -1.56<br>p = .119	b = 0.38 ± 0.49<br>z = 0.78<br>p > .250
Delay Two decades				
Model comparison	X(1) = 6.42<br>p = .011	X(1) = 0.81<br>P > .250	X(1) = 2.02<br>p = .155	X(1) = 0.72<br>p > .250
Effect	b = 0.19 ± 0.06<br>z = 3.48<br>p < .001	b = -0.01 ± 0.01<br>z = -0.84<br>p > .250	b = -0.05 ± 0.04<br>z = -1.42<br>p = .157	b = 0.45 ± 0.55<br>z = 0.82<br>p > .250

Supplementary Table 4 Temporal dynamics of perceived trustworthiness, GDP per capita and democratization in the paintings of the Web Gallery of Art. All the tests are two-sided. Following APA's recommendations, exact p-values are provided for p-values between .001 and .250. Source data are provided as raw data and scripts on the online depository.

	Affluence only		Time + Affluence		Armed conflict only		Time + Armed conflict	
	National Portraits Gallery	Web Gallery of Art	National Portraits Gallery	Web Gallery of Art	National Portraits Gallery	Web Gallery of Art	National Portraits Gallery	Web Gallery of Art
year			.11±.02<br>z = 5.46<br>p < .001	.05±.01<br>z = 3.54<br>p < .001			.14±.02<br>z = 7.55<br>p<.001	.05±.01<br>z = 4.13<br>p < .001
Number of book titles per capita	.35±.06<br>z = 6.15<br>p <.001	.29±.10<br>z = 2.77<br>p = .006	.21±.06<br>z = 3.45<br>p = .001	.14±.11<br>z = 1.26<br>p = .208				
Presence of an armed conflict					.01±.05<br>z = 0.30<br>p > .250	.00±.03<br>z =-0.01<br>p > .250	.05±.05<br>z = 1.05<br>p > .250	-.01±.03<br>z = -0.39<br>p > .250
Control variables								
Perceived dominance	-.78±.02<br>z = -40.10<br>p < .001	-.75±.02<br>z = -54.29<br>p < .001	-.79±.02<br>z = -40.85<br>p < .001	-.74±.01<br>z = -54.13<br>p < .001	-.78±.02<br>z = -39.79<br>p < .001	-.74±.01<br>z = -54.85<br>p < .001	-.79±.02 z=-40.74<br>p < .001	-.74±.02 z=-54.86<br>p < .001
Gender	-.31±.06<br>z = -5.27<br>p <.001	-.33±.03<br>z = -11.13<br>p < .001	-.29±.06<br>z = -5.09<br>p < .001	-.32±.03<br>z = -10.52<br>p < .001	-.37±.06<br>z = -6.41<br>p < .001	-.33±.03<br>z = -11.51<br>p < .001	-.33±.06<br>z = -5.68<br>p<.001	-.31±.03<br>z = - 10.49<br>p < .001

Age	-.00±.00<br>z = -1.35<br>p = .178		-.00±.00<br>z = -2.49<br>p = .013		.00±.00<br>z = 0.21<br>p > .250		-.00±.00<br>z = -2.01<br>p = .044	
Sample								
N	1962	3801	1962	3801	1962	3927	1962	3927

Supplementary Table 5 Replication analyses on perceived trustworthiness in the National Portrait Gallery and the Web Gallery of Art using the Number of book titles per capital as a proxy of affluence as well as the presence of armed conflict as indicator of periods of war and social unrest.
The first line corresponds to the regression coefficient with their associated standard error to the mean (mean ± s.e.m.). Results in bold corresponds to statistically significant effects of the variables of interest. The upper part of the table presents the effects of the variables of interest (time, affluence and democratization), while the lower part presents the effects of the control variables (perceived dominance, gender and age). All the tests are two-sided. Following APA's recommendations, exact p-values are provided for p-values between .001 and .250. Source data are provided as raw data and scripts on the online depository.

Copyright of the analysed databases

All the exploited databases (Prof. Todorov's avatar datasets, Karolinska database, Oslo Face database, Chicago Face database, FEI Face database, the National Portrait Gallery database and the Web Gallery of Art database) are free of use for non-commercial research purposes. The use of the Selfiecity database has been authorized by its owner, Dr. Lev Manovuch.

Supplementary References

1. Oosterhof, N. N. & Todorov, A. The functional basis of face evaluation. *Proc. Natl. Acad. Sci.* **105**, 11087–11092 (2008).
2. Du, S., Tao, Y. & Martinez, A. M. Compound facial expressions of emotion. *Proc. Natl. Acad. Sci.* **111**, E1454–E1462 (2014).
3. Baltrušaitis, T., Robinson, P. & Morency, L. OpenFace: An open source facial behavior analysis toolkit. in *2016 IEEE Winter Conference on Applications of Computer Vision (WACV)* 1–10 (2016). doi:10.1109/WACV.2016.7477553.
4. Todorov, A., Dotsch, R., Porter, J. M., Oosterhof, N. N. & Falvello, V. B. Validation of data-driven computational models of social perception of faces. *Emotion* **13**, 724–738 (2013).
5. Stewart, L. H. *et al.* Unconscious evaluation of faces on social dimensions. *J. Exp. Psychol. Gen.* **141**, 715–727 (2012).
6. Safra, L., Ioannou, C., Amsellem, F., Delorme, R. & Chevallier, C. Distinct effects of social motivation on face evaluations in adolescents with and without autism. *Sci. Rep.* **8**, 1–8 (2018).
