# Supplementary material for: Tracking historical changes in perceived trustworthiness in Western Europe using machine learning analyses of facial cues in paintings
Source: Nat Commun. 2020 Sep 22;11:4728. doi: 10.1038/s41467-020-18566-7 (PMC7508927; doi:10.1038/s41467-020-18566-7)
Supplement: Supplementary file 2 — Reporting Summary [file 41467_2020_18566_MOESM2_ESM.pdf]

nature research

Corresponding author(s): *Double-blind peer review submissions: write DBPR and your manuscript number here instead of author names.*

Last updated by author(s): *YYYY-MM-DD*

Reporting Summary

Nature Research wishes to improve the reproducibility of the work that we publish. This form provides structure for consistency and transparency in reporting. For further information on Nature Research policies, see Authors & Referees and the Editorial Policy Checklist.

Statistics

For all statistical analyses, confirm that the following items are present in the figure legend, table legend, main text, or Methods section.

n/a	Confirmed	
☐	☒	The exact sample size (n) for each experimental group/condition, given as a discrete number and unit of measurement
☐	☒	A statement on whether measurements were taken from distinct samples or whether the same sample was measured repeatedly
☐	☒	The statistical test(s) used AND whether they are one- or two-sided<br>*Only common tests should be described solely by name; describe more complex techniques in the Methods section.*
☐	☒	A description of all covariates tested
☐	☒	A description of any assumptions or corrections, such as tests of normality and adjustment for multiple comparisons
☐	☒	A full description of the statistical parameters including central tendency (e.g. means) or other basic estimates (e.g. regression coefficient) AND variation (e.g. standard deviation) or associated estimates of uncertainty (e.g. confidence intervals)
☐	☒	For null hypothesis testing, the test statistic (e.g. F, t, r) with confidence intervals, effect sizes, degrees of freedom and P value noted<br>*Give P values as exact values whenever suitable.*
☐	☒	For Bayesian analysis, information on the choice of priors and Markov chain Monte Carlo settings
☒	☐	For hierarchical and complex designs, identification of the appropriate level for tests and full reporting of outcomes
☐	☒	Estimates of effect sizes (e.g. Cohen's d, Pearson's r), indicating how they were calculated

Our web collection on statistics for biologists contains articles on many of the points above.

Software and code

Policy information about availability of computer code

Data collection	Bash scripts were used to download portraits for the different databases.
Data analysis	Data were analyzed using OpenFace 1.0.1, OpenCV 3.3.0 and R 3.6.1. All analyses scripts presented in the main text and in the supplementary materials are accessible online at: https://osf.io/j68xu/?view_only=61995a283e9f4c55b43c9f31d6bd1e97.

For manuscripts utilizing custom algorithms or software that are central to the research but not yet described in published literature, software must be made available to editors/reviewers. We strongly encourage code deposition in a community repository (e.g. GitHub). See the Nature Research guidelines for submitting code & software for further information.

Data

Policy information about availability of data

All manuscripts must include a data availability statement. This statement should provide the following information, where applicable:

- Accession codes, unique identifiers, or web links for publicly available datasets
- A list of figures that have associated raw data
- A description of any restrictions on data availability

All data analysed in the main text and in the supplementary materials are accessible online at: https://osf.io/j68xu/?view_only=61995a283e9f4c55b43c9f31d6bd1e97. A reporting summary for this Article is available as a Supplementary Information file. The images analysed in this article are available at: Prof. Todorov avatars: http://tlab.princeton.edu ; Chicago Face database : https://chicagofaces.org/default/ ; Oslo Face database : https://sirileknes.com/oslo-face-database/ ; Karolinska Face database : https://www.kdef.se/index.html ; FEI Face database : https://fei.edu.br/~cet/facedatabase.html ; House of Representative official portraits : https://www.house.gov/representatives ; Selfiecity : http://selfiecity.net ; National Portrait Gallery : https://www.npg.org.uk ; Web Gallery of Art : https://www.wga.hu .

Field-specific reporting

Please select the one below that is the best fit for your research. If you are not sure, read the appropriate sections before making your selection.

☐ Life sciences ☒ Behavioural & social sciences ☐ Ecological, evolutionary & environmental sciences

For a reference copy of the document with all sections, see nature.com/documents/nr-reporting-summary-flat.pdf

Behavioural & social sciences study design

All studies must disclose on these points even when the disclosure is negative.

Study description	This paper presents quantitative analysis of the evolution of trustworthiness displays in history using machine learning techniques. It aims at quantifying the historical evolution of trustworthiness displays in European paintings.
Research sample	We used portraits from the National Portrait Gallery and the Web gallery of art as they provide large samples of European portraits painted from the 14th century.
Sampling strategy	All the available portraits of these two databases were collected
Data collection	The download of the portraits was automatized using bash scripts in order to collect all the portraits available on the National Portrait Gallery and the Web Gallery of Art websites. As this collection was exhaustive, the scripts were written by Lou Safra who was not blind to the hypotheses.
Timing	Portraits from the National Portrait Gallery were downloaded on 02/04/2018 and portraits from the Web Gallery of Art were downloaded on 05/22/2018
Data exclusions	Portraits for which OpenFace's face identification points were misaligned with the real face's contours (as rated by 2 or 3 evaluated raters) were excluded. In addition, for the National Portrait Gallery, 66 portraits were excluded for not having a date of realization or for being painted after the sitter's death. In total, 547 images for the National Portrait Gallery were excluded, and 762 images were excluded for the Web Gallery of Art.
Non-participation	Not applicable, all the available portraits were collected
Randomization	Not applicable, this study exploits existing historical portraits

Reporting for specific materials, systems and methods

We require information from authors about some types of materials, experimental systems and methods used in many studies. Here, indicate whether each material, system or method listed is relevant to your study. If you are not sure if a list item applies to your research, read the appropriate section before selecting a response.

Materials & experimental systems

n/a	Involved in the study	
☒	☐	Antibodies
☒	☐	Eukaryotic cell lines
☒	☐	Palaeontology
☒	☐	Animals and other organisms
☒	☐	Human research participants
☒	☐	Clinical data

Methods

n/a	Involved in the study	
☒	☐	ChIP-seq
☒	☐	Flow cytometry
☒	☐	MRI-based neuroimaging
