# Supplementary material for: Tracking historical changes in perceived trustworthiness in Western Europe using machine learning analyses of facial cues in paintings
Source: Nat Commun. 2020 Sep 22;11:4728. doi: 10.1038/s41467-020-18566-7 (PMC7508927; doi:10.1038/s41467-020-18566-7)
Supplement: Supplementary file 3 — Original reviewer TPR [file 41467_2020_18566_MOESM3_ESM.pdf]

nature portfolio

Peer Review File

Tracking historical changes in perceived trustworthiness in Western Europe using machine learning analyses of facial cues in paintings

Open Access This file is licensed under a Creative Commons Attribution 4.0 International License, which permits use, sharing, adaptation, distribution and reproduction in any medium or format, as long as you give appropriate credit to the original author(s) and the source, provide a link to the Creative Commons license, and indicate if changes were made. In the cases where the authors are anonymous, such as is the case for the reports of anonymous peer reviewers, author attribution should be to 'Anonymous Referee' followed by a clear attribution to the source work. The images or other third party material in this file are included in the article's Creative Commons license, unless indicated otherwise in a credit line to the material. If material is not included in the article's Creative Commons license and your intended use is not permitted by statutory regulation or exceeds the permitted use, you will need to obtain permission directly from the copyright holder. To view a copy of this license, visit http://creativecommons.org/licenses/by/4.0/.

Reviewers' comments:

Reviewer #1 (Remarks to the Author):

This is a very interesting manuscript using advances in machine learning and modeling of social perception of faces to study displays of trustworthiness in portraits and how changes in these displays are related to historical changes. The results are straightforward showing that increases in trustworthiness displays correspond to increases in social trust, as measured my various indices and best predicted by increases in GDP. The time-lag analysis was informative and necessary to go a step beyond the observed correlations.

I liked the fact that the machine learning algorithm was first validated on existing human ratings before being applied to works of art. The correlations were not generally high, but this is to be expected as the FaceGen faces/ modeling doesn't capture face texture well and the latter is very important for judgments.

I have no serious concerns about the manuscript. The approach is innovative and may open new venues for psychological research on cultural artifacts.

In Fig. 1 (also Fig. 2A), there is a discernible dip in trustworthiness displays around years 1800-1850. This could be just measurement noise. Or alternatively might reflect particular historical events at the time. Were there any events in the UK that could have led to a temporary decrease in trust? I guess the bigger conceptual question is how fine grained this analysis could be. It might be that it can only capture trends over extended periods of time because of measurement limitations, including available data points. Or perhaps, it could capture more fine grained times scales given the appropriate measures.

Alex Todorov

Reviewer #2 (Remarks to the Author):

Review of „Tracking the rise of trust in history using machine learning and paintings"

The paper uses machine learning to assess trustworthiness of faces presented in paintings from 14th-21th centuries. The results show that trustworthiness increased over time, and it is predicted by raising GDP but not by rising political democratization. The paper uses novel techniques and data sources to

describe historical changes of trustworthiness. This is very exciting. On the other hand however, I would appreciate more insights into the meaning of these results and more in-depth reflection on mechanisms which may create these patterns.

Below I list substantial comments to the analysis.

1. The correlation of trustworthiness with GDP over time is interesting and suggest that security and better living conditions produce higher trustworthiness. However, I wonder how reliable data on historical GDP are, and how well they reflect living standards (especially if you are dealing with the data for 19 countries, in the analysis for the WGA). Do the results hold if, instead of GDP, you account for periods of wars and social unrest (e.g. revolutions)?

2. How should we interpret the level of trustworthiness of a portrait? Does it reflect the trustworthiness of the painter or of the sitter (who may wishes a trustworthy-looking representation of themselves). You seem to take the second view as you excluded those portraits which were painted after the sitter died (page 9 Supplementary materials). I imagine the change probably reflects cultural shift, and therefore reflects the desires of the artist, of the model, and of the audience at the same time. In my view, this issue requires some discussion. Moreover, do your results hold if you do not exclude portraits which were painted after the sitter died?

3. Your analysis controls for dominance, but its meaning is not explained in the paper. Do you expect that dominance corresponds to some cultural traits (as trustworthiness does)? All your models control for dominance, but I see no reason for it. Why would dominance be a confounder? Do the results hold if you exclude this control variable?

4. The plots S7 and S8 suggest that trustworthiness increased over time for men, whereas women were always presented as rather trustworthy. Is the gender difference statistically significant? What is the meaning of this difference? Does it mean that the change occurred only for people in position of power?

5. To test whether trustworthiness changed with GDP per capita and democratization I would recommend to use individual (rather that aggregated) data for trustworthiness and multilevel models (individual level trustworthiness as predicted by other individual variables and macro-level factors such as GDP; this method would be desirable especially for WGA data including 19 countries as it would control for clustering of portraits produced in a single country).

Points that in my view require clarification:

1. Main text page 2, lines 68-69: how to read the results for “women portraits” vs. “men portraits”? I understand that women’s portraits were evaluated as more trustworthy (with difference of 7.98) but also as more dominant (difference of 11.79), which is at odds with the typical pattern of gender difference. Is this maybe a mistake and the difference should read “-11,79”?

2. Description of gender differences is generally confusing. I found it hard to understand the direction: is it the effect of being a woman or an effect of being a man? I had the impression that you used different directions in various parts of the text (e.g. gender difference is the effect of being a man in Supplementary materials page 9 lines 2-3 from the bottom; but it seems the effect of being a woman on page 7 line 7-8 in Supplementary materials). I recommend clarifying it (e.g. “here and in other analyses presented gender difference refers to an effect of being a woman compared to being a man”) and sticking to one direction.

3. Supplementary materials page2 lines 7-8; there were 3 sets of avatars, so I imagine that one controlled for dominance and trustworthiness, one (and not two) for trustworthiness only, and one (and not two) for dominance only. Moreover, why did you choose such a setup?

4. Why is happiness missing in figure S4D?

5. Interpersonal trust vs. trustworthiness (pages 7-8 in the Supplementary materials): I do not find the main results in the section, it is illustrated in Figure S6C and D, but no test statistics or results are provided in the text.

6. Goodness of fit of points’ position seems to be an important feature but it is not introduced or explained. Moreover, it was coded as 0 or 1 and the codes were used as weights. Does it mean that pictures with poorly recognized points are excluded altogether (i.e. weigh 0)? Finally, is poor recognition correlated either with dominance or trustworthiness?

7. Supplementary materials page 8 “Table” should be “Table S2”

8. In the analysis of change over time, you use 20 years as the baseline distance and run a robustness check for 10 years. Therefore, Tables S3 and S4 should show results for delay of 2 decades first, as these are the main results. Moreover, why test robustness of 10 years but not 30? Do the conclusions hold for 30 years lag?

9. Overall, you provide very few information on the statistical method used. I imagine that I see the results of a mean difference (t-test) and OLS regressions, but it has not been specified. It is not clear to me what the “model comparison” in table S3 and S4 is, and why each table shows different test statistics.

Hope these comments are useful!
Best wishes,
Malgorzata Mikucka

Reviewer #3 (Remarks to the Author):

Referee report on ” Tracking the rise of trust in history using machine learning and online art galleries” Nature Communications

This paper attempts to trace the development of social trust in Europe since 1500. It does so by using an algorithm that automatically generates assessments of trustworthiness from a large database of historical portraits. The findings suggest that trust has increased dramatically since 1500, and in particular since 1850, and is a consequence of affluence.

I think this is a very smart paper and one that probably confirms many people’s priors. However, I also believe that the paper suffers from two truly major shortcomings, which leads me to recommend that it is rejected.

A first major problem is that the authors almost do not engage with the modern trust literature arising out of the work of Putnam (his 1993 book) and Knack and Keefre (1997 in the Quarterly Journal of Economics). The author is there not aware – or chooses to ignore – the growing literature that documents the persistence of trust. Part of this was pioneered by Uslaner (2008 in Public Opinion Quarterly). Further evidence is for example available in Berggren and Bjornskov (2011 in the Journal of Economic Behavior & Organization), as well as several studies by Ekaterina Zhuravskaya and various coauthors.

The problem is that a number of these studies clearly show how that trust is stable over rather long periods of time. No one would claim that trust differences are persistent over centuries, but the main assumption behind the present paper is that trust is malleable and reflects changes towards less violent societies and modernity. That is quite simply contrary to findings in the literature of the last decade that the authors don’t cite. Instead, they vaguely claim that their findings are “in line with historical work” (p. 2).

That the authors are ignorant of more recent work on social trust in the social sciences is also quite evident in the claim that trust can “be construed as an investment in social interactions with the potential benefits (in the event of cooperation) and also potential losses (in the event of defection).” This type of construction was popular on the first studies on social trust in the 90s that took their starting point in simple game theoretical modelling. However, soon after a number of studies debunked Putnam’s idea that social trust is created by social interaction in voluntary associations. The conceptualization of social trust – the belief that most people can be trusted instead of the assessment that known individuals are trustworthy – has changed substantially since the 1990s and quite clearly doesn’t fit with the investment theory that the present paper seems to rest upon.

The second main problem concerns the assumptions underlying the identification of trust changes over time. The authors use cues of trustworthiness in facial expressions that work with modern subjects.

However, the problem here is that the authors apply modern cues of trustworthiness to old pictures as if such cues do not change over time. In other words, their entire exercise rests on an implicit assumption that very specific behavourial norms do not change over time, but assessments of social trust and the trustworthiness of anonymous others does.

The work by Jones (2014) on the smile revolution in European paintings and photos, which the authors use, seems to suggest that this assumption is invalid. After the revolution, a smile has been perceived as a cue of benevolence and trustworthiness. However, before the smile revolution, a smiling face in a painting was more a cue of otherness and low intelligence. In old paintings, it is often easy to identify morons, because they are the only ones who are smiling at the spectator. I have to admit that I suspect that much, and perhaps all, of the increase in trust that the authors claim they identifies, is simply a consequence of changing social norms of behaviour and looks.

Overall, I therefore cannot recommend this paper for publication. It doesn’t at all engage with the modern literature on social trust and even though the methodology is very clever, the findings are inconsistent with known facts about trust.

Reviewer #4 (Remarks to the Author):

The work is an interesting application of already known facial analysis technique. The authors claimed "apply novel methods to extract quantitative information" however, I could not see any novelty in the work. Although, if the authors can conduct more detail analysis by introducing compound emotions in trustworthiness analysis, I believe the work would have enough impact to be accepted. Compound emotion work can be found at:
Guo, Jianzhu, et al. "Dominant and complementary emotion recognition from still images of faces." IEEE Access 6 (2018): 26391-26403.

Reviewer 1.

This is a very interesting manuscript using advances in machine learning and modeling of social perception of faces to study displays of trustworthiness in portraits and how changes in these displays are related to historical changes. The results are straightforward showing that increases in trustworthiness displays correspond to increases in social trust, as measured my various indices and best predicted by increases in GDP. The time-lag analysis was informative and necessary to go a step beyond the observed correlations.

I liked the fact that the machine learning algorithm was first validated on existing human ratings before being applied to works of art. The correlations were not generally high, but this is to be expected as the FaceGen faces/ modeling doesn't capture face texture well and the latter is very important for judgments.

I have no serious concerns about the manuscript. The approach is innovative and may open new venues for psychological research on cultural artifacts.

In Fig. 1 (also Fig. 2A), there is a discernible dip in trustworthiness displays around years 1800-1850. This could be just measurement noise. Or alternatively might reflect particular historical events at the time. Were there any events in the UK that could have led to a temporary decrease in trust? I guess the bigger conceptual question is how fine grained this analysis could be. It might be that it can only capture trends over extended periods of time because of measurement limitations, including available data points. Or perhaps, it could capture more fine grained times scales given the appropriate measures.

Alex Todorov

We would like to thank Pr. Todorov for his positive and very encouraging review. Concerning the precision of our measure, unfortunately we cannot provide a definitive answer to this question because our study was not designed to assess the time resolution of trustworthiness displays. Therefore, while we completely agree with Pr. Todorov about the importance of this question, additional analyses of our data would only provide speculative answers regarding the potential effects of historical events, such as the industrial revolution. Therefore, we believe that providing an estimate of the time resolution of our measure is beyond the scope of the present paper and should be investigated in the future. This could be done, for example, by using punctual external shocks on resource levels in order to assess their potential causal impact on differences in trustworthiness displays.

Reviewer 2.

1. The correlation of trustworthiness with GDP over time is interesting and suggest that security and better living conditions produce higher trustworthiness. However, I wonder how reliable data on historical GDP are, and how well they reflect living standards (especially if you are dealing with the data for 19 countries, in the analysis for the WGA). Do the results hold if, instead of GDP, you account for periods of wars and social unrest (e.g. revolutions)?

We would like to thank Dr. Mikucka for this remark. In order to assess the robustness of our results to the use of different measures of affluence, we conducted additional analyses with two other predictors of living conditions: the number of book titles per capita, which is a validated proxy of affluence (CIT) and the presence of an armed conflict (either internal or international, as suggested by Dr. Mikucka). The effect of affluence on trustworthiness displays was replicated using number of book titles per 1000 capita (which is another yet less sensitive proxy of affluence) in both the National Portrait Gallery and the Web Gallery of Art databases (see table below). However, no significant effect of armed conflicts was evidenced. This result may be explained by the fact that armed conflicts are not monotonously linked to affluence (wars can start in periods of poverty or of affluence) and that we could not find a database on the intensity of armed conflicts. We included the effect of book titles per capita as a supplementary analysis in the revised version of the manuscript.

	Affluence only		Time + Affluence		Armed conflict only		Time + Armed conflict	
	National Portraits Gallery	Web Gallery of Art	National Portraits Gallery	Web Gallery of Art	National Portraits Gallery	Web Gallery of Art	National Portraits Gallery	Web Gallery of Art
year			**.11±.02<br>z=5.46<br>p<.001**	**.05±.01<br>z=3.54<br>p<.001**			**.14±.02<br>z=7.55<br>p<.001**	.05±.01 z=<br>4.13<br>p < .001
Number of book titles per capita	**.35±.06<br>z=6.15<br>p <.001**	**.29±.10<br>z=2.77<br>p = .006**	**.21±.06<br>z=3.45<br>p=.001**	**.14±.11<br>z=1.26<br>p = .208**				
Presence of an armed conflict					**.01±.05<br>z=0.30<br>p > .250**	.00±.03 z=-<br>0.01<br>p > .250	.05±.05<br>z=1.05<br>p > .250	-.01±.03<br>z =-0.39<br>p > .250
Control variables								
Dominance	-.78±.02 z=-<br>40.10<br>p < .001	-.75±.02 z=-<br>54.29 p <<br>.001	-.79±.02 z=-<br>40.85<br>p < .001	-.74±.01 z=-<br>54.13<br>p < .001	-.78±.02 z=-<br>39.79<br>p < .001	-.74±.01 z=-<br>54.85<br>p < .001	-.79±.02 z=-<br>40.74<br>p < .001	-.74±.02 z=-<br>54.86<br>p < .001
Gender	-.31±.06 z=-<br>5.27<br>p <.001	-.33±.03 z=-<br>11.13 p <<br>.001	-.29±.06 z=-<br>5.09<br>p < .001	-.32±.03 z=-<br>10.52<br>p < .001	-.37±.06 z=-<br>6.41<br>p < .001	-.33±.03 z=-<br>11.51<br>p < .001	-.33±.06 z=-<br>5.68 p<.001	-.31±.03 z=-<br>10.49<br>p < .001
Age	-.00±.00 z=-<br>1.35<br>p =.178		-.00±.00 z=-<br>2.49<br>p =.013		.00±.00<br>z=0.21<br>p > .250		-.00±.00 z=-<br>2.01<br>p = .044	
Sample								
N	1962	3801	1962	3801	1962	3927	1962	3927

2. How should we interpret the level of trustworthiness of a portrait? Does it reflect the trustworthiness of the painter or of the sitter (who may wishes a trustworthy-looking representation of themselves). You seem to take the second view as you excluded those portraits which were painted after the sitter died (page 9 Supplementary materials). I imagine the change probably reflects cultural shift, and therefore reflects the desires of the artist, of the model, and of the audience at the same time. In my view, this issue requires some discussion. Moreover, do your results hold if you do not exclude portraits which were painted after the sitter died?

We would like to thank Dr. Mikucka for pointing out that we needed to clarify this point. The goal of our study is to document changes in social trust, that correspond to a global change in mentalities (i.e., of the artist, the model and the audience). We decided to exclude posthumous portraits as they may reflect a re-interpretation of historical figures that can be depicted with characteristics that are believed to belong to previous times. Importantly, this filter could only be applied to the National Portrait Gallery, which allows us to automatically access the sitter's information. Removing this filter did not change the significance of our results:

- Effect of GDP per capita: $b = 0.03 \pm 0.01$, $z = 7.40$, $p < .001$
  when controlling for time: $b = 0.02 \pm 0.01$, $z = 3.22$, $p = .001$
- Effect of democratization index: $b = 0.03 \pm 0.01$, $z = 5.93$, $p < .001$
  when controlling for time: $b = -0.00 \pm 0.01$, $z = -0.29$, $p > .250$
- Time-lag effect of affluence on trust: $F(40,1) = 12.36$, $p = .001$
  Time-lag effect of trust on affluence: $F(40,1) = 1.19$, $p > .250$

In the revised version of the manuscript we clearly explain that our method aims to measure the evolution of global social trust.

Main text, p.3, lines 65-66

> These shifts in cultural artefacts reveal global changes in mentalities, reflecting the preference of the sitter, the artist and the audience altogether.

Supplementary Materials, p.9

> Importantly, in order to ensure that the portraits accurately reflected the level of trust at the time the portrait was painted and to avoid re-interpretation of past historical figures, only portraits painted during the sitter's lifetime were analyzed.

3. Your analysis controls for dominance, but its meaning is not explained in the paper. Do you expect that dominance corresponds to some cultural traits (as trustworthiness does)? All your models control for dominance, but I see no reason for it. Why would dominance be a confounder? Do the results hold if you exclude this control variable?

We would like to thank Dr. Mikucka for pointing out that we needed to clarify this point. Dominance is considered, together with trustworthiness as one of the main dimensions of social interactions (Oosterhof & Todorov, 2008). More specifically, dominance is considered as a signal of power and advertising one's dominance is thus influenced by specific factors (such as the presence of a threat to social status). Although dominance and trustworthiness are two distinct traits, perceived trustworthiness and perceived dominance are nonetheless correlated (in line with the literature on first impressions, in our data correlation coefficients up to -0.46 were found, SI

p.6). For this reason, we systematically included dominance as a control variable. We provide additional explanations about this point in the revised version of the supplementary analyses. Importantly, we replicated the general pattern of results presented in the manuscript when removing dominance from the regression:

National Portrait Gallery

- Effect of GDP per capita: $b = 0.03 \pm 0.01$, $z = 3.81$, $p < .001$
  when controlling for time: $b = 0.02 \pm 0.01$, $z = 2.28$, $p = .023$
- Effect of democratization index: $b = 0.03 \pm 0.01$, $z = 3.64$, $p < .001$
  when controlling for time: $b = -0.01 \pm 0.02$, $z = -0.64$, $p > .250$
- Time-lag effect of affluence on trust: $F(41,1) = 1.63$, $p = .209$
  Time-lag effect of trust on affluence: $F(41,1) = 3.42$, $p = .072$

Web Gallery of Art

- Effect of GDP per capita: $b = 0.12 \pm 0.05$, $z = 2.37$, $p = .018$
  when controlling for time: $b = 0.02 \pm 0.05$, $z = 0.46$, $p >.250$
- Effect of democratization index: $b = -0.01 \pm 0.01$, $z = -1.73$, $p = .084$
  when controlling for time: $b = -0.12 \pm 0.18$, $z = -0.65$, $p > .250$
- Time-lag effect of affluence on trust: $X(1) = 8.754$, $p = .003$
  Time-lag effect of trust on affluence: $X(1) = 0.00$, $p > .250$

Supplementary Materials, p.1

> In order to quantify trustworthiness displays in historical paintings, we developed an algorithm automatically estimating perceived trustworthiness from faces. Our algorithm also extracted perceived dominance since dominance has been shown to be, together with trustworthiness, one of the main dimensions of social perception (1). Crucially, although dominance displays carry signals of power that are distinct from the cooperation-related signals associated with trustworthiness displays, perceived dominance and perceived trustworthiness are correlated (1). This correlation entails that it is of paramount importance to control for perceived dominance when analyzing perceived trustworthiness.

4. The plots S7 and S8 suggest that trustworthiness increased over time for men, whereas women were always presented as rather trustworthy. Is the gender difference statistically significant? What is the meaning of this difference? Does it mean that the change occurred only for people in position of power?

Following Dr. Mikucka comment, we tested the interaction between gender and time and found no significant effect in both the National Portrait Gallery ($b = 0.04 \pm 0.04$, $z = 0.87$, $p >.250$) and the Web Gallery of Art databases ($b = 0.03 \pm 0.02$, $z = 1.33$, $p = .185$). Nonetheless, we further tested the hypothesis that only people in position of power displayed a change in trustworthiness by testing the effect of having an aristocratic or prestigious title on the evolution of trustworthiness in the National Portrait Gallery. No significant effect was evidenced (main effect: $b = 0.26 \pm 0.64$, $z = 0.40$, $p > .250$; interaction with time: $b = -0.01 \pm 0.04$, $z = -0.31$, $p > .250$) suggesting that the increase in trustworthiness displays was not limited to people in position of power.

5. To test whether trustworthiness changed with GDP per capita and democratization I would recommend to use individual (rather that aggregated) data for trustworthiness and multilevel models (individual level trustworthiness as predicted by other individual variables and macro-level factors such as GDP; this method would be desirable especially for WGA data including 19 countries as it would control for clustering of portraits produced in a single country).

We would like to thank Dr. Mikucka for pointing out that we needed to clarify the analyses we conducted. Indeed, we conducted individual analyses for GDP per capita and democratization, with two-level models for the Web Gallery of Art database. The only aggregated analyses we conducted were time-lag analyses as it required to analyze the effect of trustworthiness displays at a specific time point on the GDP per capita decades later. We clarified the presentation of our analyses plan in the revised version of the manuscript.

Supplementary Materials, p.10

> A total of 1943 data points were included in the analyses looking at the effect of GDP per capita. A total of 1115 data points were included in the analyses looking at the effect of Polity2. Paintings were analyzed using individual linear models (each painting corresponding to one data point), taking the sitter's gender, age and level of dominance as control variables.

Supplementary Materials, p.10

> Finally, time-lag analyses were conducted to analyze the temporal dynamics between trustworthiness, GDP per capita and democratization. To do so, data were averaged by decades and analyzed at the aggregated level.

Supplementary Materials, p.11

> As for the National Portrait Gallery, the missing levels of affluence and democratization were completed using the previous complete value. The same models as previously were used except that a random effect was included to taki the localization of the paintings into account. This resulted, for the analysis of the effect of GDP per capita and democratization in two-level mixed models, taking painting as individual data points clustered by country of production. Correspondingly, for time-lag analyses, we use two-level mixed models but with data aggregated by decades.

Points that in my view require clarification:

1. Main text page 2, lines 68-69: how to read the results for "women portraits" vs. "men portraits"? I understand that women's portraits were evaluated as more trustworthy (with difference of 7.98) but also as more dominant (difference of 11.79), which is at odds with the typical pattern of gender difference. Is this maybe a mistake and the difference should read "-11,79"?

We would like to thank Dr. Mikucka for spotting this typo, it has been corrected in the revised version of the manuscript.

2. Description of gender differences is generally confusing. I found it hard to understand the

direction: is it the effect of being a woman or an effect of being a man? I had the impression that you used different directions in various parts of the text (e.g. gender difference is the effect of being a man in Supplementary materials page 9 lines 2-3 from the bottom; but it seems the effect of being a woman on page 7 line 7-8 in Supplementary materials). I recommend clarifying it (e.g. “here and in other analyses presented gender difference refers to an effect of being a woman compared to being a man”) and sticking to one direction.

We would like to thank Dr. Mikucka for this comment: we only presented the analysis of being a woman in the revised version of the manuscript.

Main text, Table 1

	Time-only		Affluence only		Time + Affluence		Democratization only		Time + Democratization	
	National Portraits Gallery	Web Gallery of Art	National Portraits Gallery	Web Gallery of Art	National Portraits Gallery	Web Gallery of Art	National Portraits Gallery	Web Gallery of Art	National Portraits Gallery	Web Gallery of Art
year	**.14±.02<br>z=7.49<br>p< .001**	**.07±.01<br>z=5.33<br>p< .001**			**.08±.03<br>z=3.17<br>p=.002**	**.06±.02<br>z=2.87<br>p=.007**			**.32±.11<br>z=2.86<br>p=.004**	-.13±.14<br>z=-.98<br>p > .250
GDP per capita			**.03±.00<br>z=7.13<br>p <.001**	**.09±.03<br>z=3.16<br>p = .002**	**.02±.01<br>z=3.16<br>p=.002**	**.07±.04<br>z=1.98<br>p = .048**				
Democracy index							**.03±.01<br>z=5.24<br>p < .001**	-.01±.01<br>z=-1.96<br>p = .051	-.01±.01<br>z=-0.50<br>p > .250	-.01±.01<br>z =-.96<br>p > .250
Control variables										
Dominance	-.79±.02<br>z=-40.74<br>p < .001	-.74±.01<br>z=-56.58<br>p < .001	-.78±.02<br>z=-40.10<br>p < .001	-.75±.02<br>z=-46.29<br>p < .001	-.78±.02<br>z=-40.30<br>p < .001	-.74±.02<br>z=-46.05<br>p < .001	-.77±.03<br>z=-30.76<br>p < .001	-.71±.04<br>z=20.17<br>p < .001	-.77±.03<br>z=-30.83<br>p < .001	-.71±.04<br>z=-20.17<br>p < .001
Gender	.32±.06<br>z=5.64<br>p < .001	.31±.03<br>z=10.76<br>p < .001	.29±.06<br>z=5.01<br>p <.001	.30±.04<br>z=8. 31 p < .001	.30±.06<br>z=5.10<br>p < .001	.29±.04<br>z=7.98<br>p < .001	.28±.08<br>z=3.61<br>p < .001	.25±.07<br>z=3.30<br>p = .001	.25±.08<br>z=3.16<br>p=.002	.25±.07<br>z=3.37<br>p < .001
Age	-.00±.00<br>z=-2.03<br>p=.043		-.00±.00<br>z=-1.88<br>p=.060		-.00±.00<br>z=-2.26<br>p=.024		.00±.00<br>z=0.48<br>p > .250		-.00±.00<br>z=-0.17<br>p > .250	
Sample										
N	1962	4106	1943	2706	1943	2706	1115	565	1115	565

Table 1 – Effect of time, GDP per capita and democratization on the portraits of National Portrait Gallery and the Web Gallery of Art

Supplementary Materials, p.5

gender effect (females appear as less dominant and more trustworthy than males; trustworthiness: real effect: $t(768) = 7.94$, $p < .00$; recovered effect: $t(972) = 2.67$, $p = .008$; dominance: real effect: $t(769) = -7.80$, $p <.001$; recovered effect: $t(972) = -3.63$, $p <.001$; Figure S4A-B)

Supplementary Materials, p. 9

The information about the sitters’ gender and age allowed us to replicate the classic findings that older sitters appear more dominant and less trustworthy than younger sitters and that female sitters appear more dominant and less trustworthy than male sitters (trustworthiness: gender effect: $t(1960) = 9.69$, $p < .001$; age effect: $t(1960) = -6.63$, $p < .001$; dominance: gender effect: $t(1960) = 7.24$, $p < .001$; age effect: $t(1960) = -9.12$, $p < .001$; Figure S7).

Supplementary Materials, p.11

As for the NPG, we accurately recovered the gender effect on trustworthiness and dominance on the portraits of the WGA (trustworthiness: $z = 17.70$, $p < .001$; dominance: $z = -13.35$, $p < .001$; Figure S8).

3. Supplementary materials page2 lines 7-8; there were 3 sets of avatars, so I imagine that one controlled for dominance and trustworthiness, one (and not two) for trustworthiness only, and one (and not two) for dominance only. Moreover, why did you choose such a setup?

We would like to thank Dr. Mukcka for spotting this typo, we actually optimized our model on five sets of avatars, corresponding to all the available validated sets of avatars generated with Facegen.

Supplementary Materials, p.2

We built a model that automatically extracts trustworthiness and dominance evaluations from the facial action units detected by the OpenFace algorithm (OpenFace version 1.01 using OpenCV 3.3.0 (5)). To do so, we extracted the facial action units of five sets of avatars generated with Facegen and controlled for dominance, trustworthiness or both dominance and trustworthiness (Fig. S1) (6). More precisely, one set of avatars was controlled for both dominance and trustworthiness (N = 49), two controlled for trustworthiness only (each N = 175) and two controlled for dominance only (each N = 175). These five sets of avatars correspond to all the available validated avatars controlled for trustworthiness or dominance generated by Facegen.

4. Why is happiness missing in figure S4D?

Happiness was not included for the validation of dominance estimates as no strong association between happiness and dominance has been found in the literature. On the contrary, higher levels of dominance have been shown to be associated with higher levels of anger and higher levels of trustworthiness to be associated with higher levels of happiness and lower levels of anger ((e.g., Said et al., 2009; Oosterhof & Todorov, 2009).

Said, C. P., Sebe, N., & Todorov, A. (2009). Structural resemblance to emotional expressions predicts evaluation of emotionally neutral faces. *Emotion*, *9*(2), 260.

Oosterhof, N. N., & Todorov, A. (2009). Shared perceptual basis of emotional expressions and trustworthiness impressions from faces. *Emotion*, *9*(1), 128.

5. Interpersonal trust vs. trustworthiness (pages 7-8 in the Supplementary materials): I do not find the main results in the section, it is illustrated in Figure S6C and D, but no test statistics or results are provided in the text.

The statistics associated with the results presented in Figure S6 are presented in the main text but we agree with Dr. Mikucka that this can be confusing for the reader. In the revised version of the manuscript we repeated these information in the supplementary results section, next to the associated figure.

Supplementary Materials, p.8

In line with our hypotheses, people located in places where interpersonal trust and cooperation are higher, displayed higher levels of trustworthiness in their selfies (cooperation level: $b = 0.13 \pm$

> 0.03, $z = 3.67$, $p < .001$; trust level: $b = 0.81 \pm 0.23$, $z = 3.50$, $p < .001$; Figure S6C-D)

6. Goodness of fit of points' position seems to be an important feature but it is not introduced or explained. Moreover, it was coded as 0 or 1 and the codes were used as weights. Does it mean that pictures with poorly recognized points are excluded altogether (i.e. weigh 0)? Finally, is poor recognition correlated either with dominance or trustworthiness?

We agree with Dr. Mikucka that this procedure is too succinctly presented in the manuscript, we explain it in more details in the revised version of the manuscript. Indeed, we sum the goodness of fit of points' position of multiple raters for each database, which resulted in the exclusion of only the faces for which all raters agreed that they were not well detected. We added a clearer explanation of this methodological point in the revised version of the manuscript. We found no significant difference in dominance or in trustworthiness between the faces that were excluded and those that were included (all *p*s > .250), except for the faces excluded from the National Portrait Gallery database, which were more trustworthy that those included ($b = -0.20 \pm 0.07$, $z = -2.65$, $p = .008$).

Supplementary Materials, p.7

> The identified faces were then individually analyzed by two independent raters who were asked to evaluate, for each picture, the alignment of the OpenFace's face identification points compared to the real face's contours (coded as 0 or 1). The sum of these goodness of fit was then used as weights for the analyses. Therefore, only faces for which the two raters agreed that they were not well detected were removed from the analyses. Faces for which the two raters agreed on their good detection had a weight of 2 in the analyses, and those on which they disagreed had a weight of 1.

7. Supplementary materials page 8 "Table" should be "Table S2"

Thank you, we corrected it.

8. In the analysis of change over time, you use 20 years as the baseline distance and run a robustness check for 10 years. Therefore, Tables S3 and S4 should show results for delay of 2 decades first, as these are the main results. Moreover, why test robustness of 10 years but not 30? Do the conclusions hold for 30 years lag?

Thank you, we changed for the order of presentation of the results in Table S3. We were interested only in the temporal causality and not in potentially long-lasting effects of affluence on social trust as the long- vs short-lasting effects of resources on trust is still an ongoing debate in the literature. The analysis by decades is thus a trade-off between the temporal resolution of our variables of interest and the willingness to look at effects that are shorter than a generation. However, the analyses for the 30-year lag were congruent with the results obtained for the 10- and 20-year lags (National Portrait Gallery: effect of affluence on trustworthiness displays three decades later: $F(36,1) = 13.09$, $p < .001$, effect of trustworthiness displays on affluences three

decades later: $F(36,1) = 1.17$, $p > .250$; Web Gallery of Art: effect of affluence on trustworthiness displays three decades later: $X(1) = 1.96$, $p = .161$, effect of trustworthiness displays on affluences three decades later: $X(1) = 0.01$, $p > .250$)

9. Overall, you provide very few information on the statistical method used. I imagine that I see the results of a mean difference (t-test) and OLS regressions, but it has not been specified. It is not clear to me what the "model comparison" in table S3 and S4 is, and why each table shows different test statistics.

We would like to thank Dr. Mikucka for pointing out the lack of details on the methods we used. This is now corrected in the revised version of the manuscript (Supplementary Materials section).

Supplementary Materials, p.11, legend of Table S3

> **Table S3** Temporal dynamics of trustworthiness, GDP per capita and democratization in the paintings of the National Portrait Gallery. Model comparison corresponds to the comparison of the model that included the delayed variable of interest with the model in which this variable was excluded. Effect corresponds to the estimation of the regression coefficient of the delayed variable of interest.

Reviewer #3 (Remarks to the Author):

Referee report on " Tracking the rise of trust in history using machine learning and online art galleries" Nature Communication

This paper attempts to trace the development of social trust in Europe since 1500. It does so by using an algorithm that automatically generates assessments of trustworthiness from a large database of historical portraits. The findings suggest that trust has increased dramatically since 1500, and in particular since 1850, and is a consequence of affluence.

I think this is a very smart paper and one that probably confirms many people's priors. However, I also believe that the paper suffers from two truly major shortcomings, which leads me to recommend that it is rejected.

A first major problem is that the authors almost do not engage with the modern trust literature arising out of the work of Putnam (his 1993 book) and Knack and Keefre (1997 in the Quarterly Journal of Economics). The author is there not aware – or chooses to ignore – the growing literature that documents the persistence of trust. Part of this was pioneered by Uslaner (2008 in Public Opinion Quarterly). Further evidence is for example available in Berggren and Bjornskov (2011 in the Journal of Economic Behavior & Organization), as well as several studies by Ekaterina Zhuravskaya and various coauthors.

We thank the reviewer for this point, we now cite this literature in the revised version of our paper (Putnam, 1993; Knack and Keefer, 1997; Uslaner, 2002 in both the introduction and the conclusion). While we were fully aware of the literature on the persistence of differences across countries, we chose to focus our manuscript (and therefore the cited papers) on the rise of social trust in all countries. Moreover, and as explained in more details in the response to the next point, the persistence of differences across countries and the rise of social trust in all countries are compatible (i.e. trust increases in all countries, but the place where social trust is higher remains the same).

The problem is that a number of these studies clearly show how that trust is stable over rather long periods of time. No one would claim that trust differences are persistent over centuries, but the main assumption behind the present paper is that trust is malleable and reflects changes towards less violent societies and modernity. That is quite simply contrary to findings in the literature of the last decade that the authors don't cite. Instead, they vaguely claim that their findings are "in line with historical work" (p. 2).

We totally agree with the reviewer that they are stable differences in social trust across time. Yet, the persistence of trust differences is not incompatible with a concomitant growth in trust over time. Consider for instance GDP per capita. There has been large and persistent differences in GDP per capita (between Northern and Southern Europe or between Western and Eastern Europe for instance, see Fouquet & Broadberry, 2017 for instance), and yet, GDP per capita has grown massively in the last 500 years, in all parts of Europe (see again Fouquet & Broadberry, 2017). Therefore, demonstrating an increase in social trust (as we do in the paper) is fully compatible with the persistence of differences in social trust across countries (and within countries).

Fouquet, R., & Broadberry, S. (2015). Seven centuries of European economic growth and decline. *Journal of Economic Perspectives*, *29*(4), 227-44.

That the authors are ignorant of more recent work on social trust in the social sciences is also quite evident in the claim that trust can "be construed as an investment in social interactions with the potential benefits (in the event of cooperation) and also potential losses (in the event of defection)." This type of construction was popular on the first studies on social trust in the 90s that took their starting point in simple game theoretical modelling. However, soon after a number of studies debunked Putnam's idea that social trust is created by social interaction in voluntary associations. The conceptualization of social trust – the belief that most people can be trusted instead of the assessment that known individuals are trustworthy – has changed substantially since the 1990s and quite clearly doesn't fit with the investment theory that the present paper seems to rest upon.

The reviewer is right. Our paper is grounded in game theoretical modelling, more specifically in evolutionary game modelling (Berg, Dickhaut, & McCabe, 1995 ; Mohtashemi, & Mui, 2003; Nowak & Sigmund, 2005). This is a widely shared framework in behavioral sciences, including in behavioral social sciences.

Nowak, M. A., & Sigmund, K. (2005). Evolution of indirect reciprocity. *Nature*, *437*(7063), 1291.

Berg, J., Dickhaut, J., & McCabe, K. (1995). Trust, reciprocity, and social history. *Games and economic behavior*, *10*(1), 122-142.

Mohtashemi, M., & Mui, L. (2003). Evolution of indirect reciprocity by social information: the role of trust and reputation in evolution of altruism. *Journal of theoretical biology*, *223*(4), 523-531.

The second main problem concerns the assumptions underlying the identification of trust changes over time. The authors use cues of trustworthiness in facial expressions that work with modern subjects. However, the problem here is that the authors apply modern cues of trustworthiness to old pictures as if such cues do not change over time. In other words, their entire exercise rests on an implicit assumption that very specific behavourial norms do not change over time, but assessments of social trust and the trustworthiness of anonymous others does.

This is a very crucial point. However, this is not an implicit assumption. We have been very explicit about that (lines 53-55):

> 'Experimental work have indeed revealed that specific facial features, such as a smiling mouth or wider eyes, are consistently recognized as cues of trustworthiness across individuals and cultures (14–19).'

And we cite the following studies:

14. Walker M, Jiang F, Vetter T, Sczesny S. Universals and cultural differences in forming personality trait judgments from faces. Soc Psychol Personal S 200 ci. 2011;2(6):609–617.

15. Xu F, Wu D, Toriyama R, Ma F, Itakura S, Lee K. Similarities and differences in Chinese and Caucasian adults' use of facial cues for trustworthiness judgments. PloS One. 2012;7(4):e34859.
16. Bente G, Dratsch T, Kaspar K, H..ler T, Bungard O, Al-Issa A. Cultures of Trust: Effects of Avatar Faces and Reputation Scores on German and Arab Players in an Online Trust-Game. PLOS ONE.
205
17. Engell AD, Haxby JV, Todorov A. Implicit Trustworthiness Decisions: Automatic Coding of Face Properties in the Human Amygdala. J Cogn Neurosci. 22 ao.t 2007;19(9):1508-19.
18. Birk.s B, Dzhelyova M, L.badi B, Bereczkei T, Perrett DI. Cross-cultural perception of trustworthiness: The effect of ethnicity features on evaluation of faces' observed trustworthiness across four samples. Personal Individ. Differ. 1 oct 2014;69:56-61.
210 19. Todorov A, Olivola CY, Dotsch R, Mende-Siedlecki P. Social Attributions from Faces: Determinants, Consequences, Accuracy, and Functional Significance. Annu Rev Psychol. 3 janv 2015;66(1):519-45.

More generally, since the seminal work of Eckman (1971), a large body of empirical studies has shown that facial cues of emotions are stable across societies.

Ekman, P., & Friesen, W. V. (1971). Constants across cultures in the face and emotion. *Journal of personality and social psychology*, *17*(2), 124.

We also tested the cross-cultural validity of our method by studying the SelfieCity database (pictured analyzed N = 2277). This database includes high income countries (UK, USA, Germany) and middle-income countries (Russia, Brazil, Thailand), Western (UK, USA, Germany) countries and non-Western countries (Thailand, and Brazil to some extant). Despite the cultural variability of social norms of trust, cooperation and self-presentation, we observed that people located in places where interpersonal trust and cooperation are higher (as assessed in the European and World Value Surveys) displayed higher levels of trustworthiness in their selfies.

Finally, another evidence that facial cues of trustworthiness have not changed are artistic works from the past such as Charles Le Brun's *Méthode pour apprendre à dessiner les passions* or Franz Xaver Messerschmidt's busts, which show that the facial cues associated with basic emotions such as sadness or anger have not changed in the last 300 years.

The work by Jones (2014) on the smile revolution in European paintings and photos, which the authors use, seems to suggest that this assumption is invalid. After the revolution, a smile has been perceived as a cue of benevolence and trustworthiness. However, before the smile revolution, a smiling face in a painting was more a cue of otherness and low intelligence. In old paintings, it is often easy to identify morons, because they are the only ones who are smiling at the spectator. I have to admit that I suspect that much, and perhaps all, of the increase in trust that the authors claim they identifies, is simply a consequence of changing social norms of behaviour and looks.

We totally agree that the social meaning of smiling must have changed during the period. In fact, this change is compatible with our results. In a low-trust society, one should not smile to others as they are no good reason to look nice (one should rather look dominant and aggressive to prevent aggression). Thus, only morons smile as the Reviewer rightly notes. But as trust increases, it becomes more rational to smile, because one is going to expect more and more peaceful and

cooperative interactions. What we quantify is precisely the ‘changing social norms of behaviour and looks’ (Reviewer 3) regarding trust and cooperation. More people smile because social behaviors, social interactions, social norms have changed and have become more cooperative. In this context, it is not irrational anymore to smile and to be nice. Rather, it becomes the best strategy.

Also, note that humans display different kinds of smiles. One important distinction is between the Duchenne smile (which triggers cues of trustworthiness) and non-Duchenne smiles, which are much less pro-social and can actually be aggressive (for instance, the so-called ‘rictus sardonicus’ (Jones, 2014) of the 18th c.). What we predict is not simply that people will smile more as trust increases, but that the kind of smile they display will change over time and become associated with cues of trustworthiness (and not aggressivity). In other words, they should display more and more Duchenne prosocial smiles. This is what historians observe: the smiles depicted in the oldest painting tend to be ‘snooty and aggressive’ (Jones, 2014), while the smiles depicted in the later periods are much trustworthy and Duchenne like (see Jones 2014 again, chapters 1-3). Compare for instance the smile of the famous portrait of Antonello de Messine (1470) to the smile of Vigée-Le Brun in her auto-portrait (1786). They both smile but our intuition, as well as our software, tell us that the man’s smile is much less trustworthy than Vigée-Le Brun’s smile (-0,24 compare to +0,42) This suggests that what has changed in not just the ‘cultural meaning’ of the smile, but also the facial appearance of the smile. People used more prosocial smiles in order to display more prosocial dispositions.

Example of untrustworthy smile: Portrait of an unknown man
by Antonello de Messine, 1470

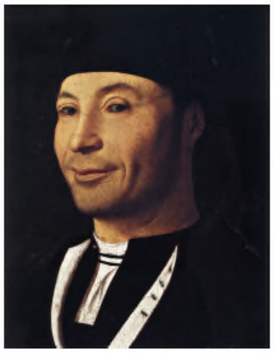

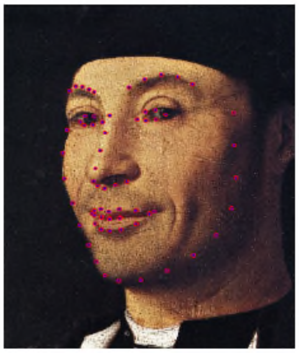

Estimated trustworthiness:
-0.24

Example of a trustworthy smile: Madame Vigée-Le Brun et sa fille
by Elisabeth-Louise Vigée-Le Brun, 1786

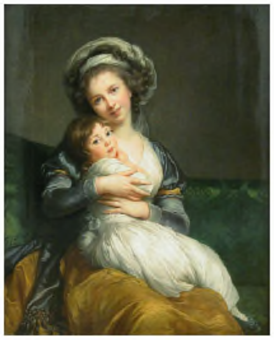

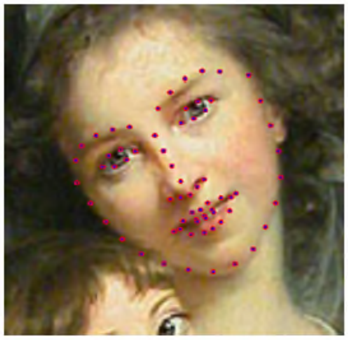

Estimated trustworthiness:
0.42

Reviewer 4.

The work is an interesting application of already known facial analysis technique. The authors claimed "apply novel methods to extract quantitative information" however, I could not see any novelty in the work. Although, if the authors can conduct more detail analysis by introducing compound emotions in trustworthiness analysis, I believe the work would have enough impact to be accepted. Compound emotion work can be found at: Guo, Jianzhu, et al. "Dominant and complementary emotion recognition from still images of faces." IEEE Access 6 (2018): 26391-26403.

We would like to thank Reviewer 4 for their comment. In the revised version of the manuscript, we now clearly specify that the novelty of our work rests in the use of vision machine learning techniques to historical datasets, notably to pictorial representations.

Main text, p.3, line 70

> In this paper, we apply recent machine-learning methods to extract quantitative information about the evolution of social cues contained in portraits.

Moreover, in line with previous research on compound emotions showing the importance of analyzing multiple social signals simultaneously for studying social evaluations, we systematically estimated both trustworthiness and dominance displays in the present studies. Indeed, dominance and trustworthiness have been identified as the two main dimensions of social evaluations. Thus, it appeared of paramount importance to control for the evolution of dominance while analyzing the evolution of trustworthiness displays. In the revised version of the manuscript, we provide more details about our methods and about the importance of controlling for dominance as well as the links between this framework and compound emotions.

Supplementary Materials, p.2

> In order to quantify trustworthiness displays in historical paintings, we developed an algorithm automatically estimating perceived trustworthiness from faces. Our algorithm also extracted perceived dominance since dominance has been shown to be, together with trustworthiness, one of the main dimensions of social perception (1). Crucially, although dominance displays carry signals of power that are distinct from the cooperation-related signals associated with trustworthiness displays, perceived dominance and perceived trustworthiness are correlated (1). This correlation entails that it is of paramount importance to control for perceived dominance when analyzing perceived trustworthiness. This type of analysis, studying together distinct but related social signals, has already been shown to be particularly promising in the emotion domain by revealing the importance of taking into account the existence of compound emotions (2).

Reviewers' comments:

Reviewer #1 (Remarks to the Author):

I was already favorably predisposed to this manuscript. The revision addresses many of the critical concerns of the other reviewers. It is an interesting, creative approach to quantitatively test hypotheses about cultural, historical changes using cultural artifacts.
Alex Todorov

Reviewer #2 (Remarks to the Author):

Dear Nicolas,
I read the new version of your manuscript with interest, I am also satisfied with the additional explanations provided in response to the reviews.
I only have minor additional comments.
1. Correlation between trustworthiness and dominance is not a sufficient reason to treat it as a confounder and control for it in the analysis. You need it as a control if it is correlated with both trustworthiness and time (or Gdp) but does not mediate the relationship between the two.
2.I believe that on p. 9 of SM (l. 196) is there is a typo, as women should be more and not less trustworthy than men.
3. Page 4 l. 113 - do you mean "proxy of the level of social trust"? To my knowledge, researchers generally measure the levels of social trust and not its importance.

Reviewer #3 (Remarks to the Author):

I have reviewed the revised version of the manuscript and can first of all note that it has improved. The authors have taken most of my comments into consideration. While I am still far from convinced, I think the paper and the main idea and method behind the paper are so interesting that it deserves publication. I therefore believe that it can be published in its current form. I'm looking forward to future studies that delve deeper into this important topic.

Reviewer #5 (Remarks to the Author):

REVIEWER STATEMENT

My review was requested to evaluate the appropriateness of the machine learning techniques used in this submission. As such, I focus on this question and (mostly) defer to the other reviewers to evaluate the other aspects of the work.

SUMMARY

In this submission, the authors created a dataset of simulated faces using the Facegen software. Features were then extracted from these simulated faces in the form of facial action units using OpenFace. Predictive models were finally trained using GLM, linear SVM, and random forest algorithms using a non-independent train-test split. The authors attempted to provide validity evidence by applying these models to several available photograph images before ultimately applying them in the current submission to portrait paintings from different centuries in history. Overall, more details about this process are required for the work to be understandable and reproducible. Much of the supplemental materials felt rushed and underdescribed. I also found some of the validity evidence to be underwhelming or only indirectly relevant to the current study. As such, I recommend revisions focused on clarification/explication and further validation in the specific domain of portrait paintings.

DETAILS

Please provide more details about the five “sets of avatars” and what is meant by “controlled for dominance, trustworthiness, or both.” I can think of two different interpretations. First, I take it that Facegen provides different baseline faces that can be morphed using parametric controls to have slightly different facial structure or expressions. Perhaps the different “sets of avatars” correspond to five different baseline faces with parametric controls for dominance, trustworthiness, or both. Thus, the final images were created from these baseline faces by setting (i.e., controlling) these parametric controls, resulting in similar faces expected to be perceived differently based on the structural and expressive changes caused by the parametric control. Alternatively, perhaps avatars with pre-existing perceptual ratings were available and therefore they were not morphed but just used as-is. Please confirm what was done and revise the section for clarity. I’d also recommend replacing figure S1 with one showing a sample of faces from all five sets. Furthermore, if morphing was done, then I assume there was some sort of numerical input to set the face to a certain position on each dimension. If so, please explain what numerical values were selected and why the final Ns were 49, 175, 175, 175, and 175.

For each simulated face image, features were extracted using the OpenFace toolkit. Specifically, it seems that estimates of facial action units were used. If so, which action units? All of them or only those with a certain amount of variability? Were the binary classification results for action units used or the pseudo-continuous estimates of action unit intensity? Given that action units represent expressive motion and not structural differences, the models seem to be using only a subset of the information available to human perceivers. Using the positions of the facial landmark points, as well as the histogram of oriented gradients (HOG) features, provided by OpenFace in addition to the action units would overcome this limitation without requiring technical expertise in computer vision. Adding experiments with these features included would be interesting and may improve predictive performance. It would also lessen the reliance on OpenFace’s ability to accurately detect low intensity, infrequent, and overlapping action units in data it wasn’t trained on, which are all notoriously difficult tasks (and are not being validated here).

Based on the Ns reported, this dataset had a total of 399 simulated facial images – a small set for machine learning but probably adequate for relatively simple models like GLM, random forest, and linear SVM. However, I do wonder why the authors did not elect to train the models on data from both Facegen and the photograph-based "validation" datasets. An explicit rationale for this decision would be appreciated. The authors' dataset was then partitioned into a training set (80%) and testing set (20%). It is stated that "the percentage of avatars coming from each database was equal in the training and test samples for both trustworthiness and dominance" but the meaning of this was not clear as the word "database" had not been defined in this context/section. Perhaps the authors meant the sets of avatars? Given that there were five databases and 1/5 of the data was used for testing, it seems that a leave-one-database-out evaluation would have made more sense. To the extent that there were dependencies and similarities within databases (e.g., if a morphing approach was used, then the faces would be quite similar within a database), this would have also been a more rigorous approach in that it would have estimated the algorithm's ability to generalize to new databases (which can be challenging in many domains). I suppose the downside of this approach would have been that the databases were not equal in size. However, given that the authors used 20-fold cross-validation, they do not seem overly concerned with the size of the testing/evaluation sets. Some rationale for this decision would be good.

More details about the implementation of the machine learning models are required for review and reproducibility. Were these models estimated in R, python, etc.? How were the hyperparameters optimized (e.g., grid search over what values)? How were the features selected? What family and link functions were selected for GLM? Why was a linear SVM selected (e.g., rather than an RBF kernel)?
In Table S1, clarify in the caption what the margins of error correspond to (e.g., SD, SE, or CI); it may also help some readers to note for each metric whether higher or lower numbers indicate better predictions. Please clarify what is meant by "with a number of variabled samples as candidates at each split equals 9." Figure S2 seems to be missing a caption and it was not described in text where these three different legend values came from (e.g., maximally distinct faces and Caucasian faces). In describing the four different face databases (Karolinska, Oslo, Chicago, FEI), please provide the number of images provided in each; this is important for interpreting the significance of the correlations. Figure S3 appears to be missing a caption and also visualization of the FEI database. I would also say that correlation scores of 0.22 and 0.16 between predictions and human perceptions are pretty underwhelming. The fact that they are significantly different from zero is not very impressive given the apparent sample size. These results again make me wonder if a model with more training data (e.g., combining the avatar and photograph data) and more rich feature representations (e.g., including landmark positions and HOG features) would perform better. That said, I did find the ability to reproduce classical findings in social cognition to be more persuasive and was glad that those experiments were included.

Although replicating gender and political party differences in portraits from Google is an interesting and indirect form of validation for the prediction models, I would like to see a subsample of paintings from the NPG and WGA rated for trustworthiness and dominance by human perceivers (e.g., on a web-based service like Mechanical Turk or Prolific) and directly compared to the algorithms' predictions. Good performance here (e.g., correlations above 0.5) would be very reassuring.

As a final point, stepping away from my methodological focus for a moment, I would agree with one of the other reviewers that it is important to consider whether the cues that indicate trustworthiness and dominance are stable over both time and culture. The authors make some argument about the apparent cultural stability of emotion and interpersonal perceptions (which should be noted is a controversial topic), but do not discuss the temporal stability piece. Without a time machine, this will be difficult to study, but it is worth mentioning as a limitation and discussion point that the same cues we would today perceive as trustworthy or dominant may have been interpreted in a different way when the portraits were created. This fact would make the use of an interpretable model more desirable, as we could at least be sure the current study was contributing a description of how facial appearance changed over time. However, I would not recommend that be explored here unless the authors also add some validation evidence regarding the action unit measures.

-Jeffrey M. Girard

Reviewer #5 (Remarks to the Author):

REVIEWER STATEMENT

My review was requested to evaluate the appropriateness of the machine learning techniques used in this submission. As such, I focus on this question and (mostly) defer to the other reviewers to evaluate the other aspects of the work.

SUMMARY

In this submission, the authors created a dataset of simulated faces using the Facegen software. Features were then extracted from these simulated faces in the form of facial action units using OpenFace. Predictive models were finally trained using GLM, linear SVM, and random forest algorithms using a non-independent train-test split. The authors attempted to provide validity evidence by applying these models to several available photograph images before ultimately applying them in the current submission to portrait paintings from different centuries in history. Overall, more details about this process are required for the work to be understandable and reproducible. Much of the supplemental materials felt rushed and underdescribed. I also found some of the validity evidence to be underwhelming or only indirectly relevant to the current study. As such, I recommend revisions focused on clarification/explication and further validation in the specific domain of portrait paintings.

We would like to thank Dr. Girard for his careful reading of our manuscript and for raising our attention to the points which lack clarity and more details. We added the requested explanations in the revised version of the manuscript which, we hope, provide, together will the online scripts, all the necessary information to understand and replicate our work.

DETAILS

Please provide more details about the five “sets of avatars” and what is meant by “controlled for dominance, trustworthiness, or both.” I can think of two different interpretations. First, I take it that Facegen provides different baseline faces that can be morphed using parametric controls to have slightly different facial structure or expressions. Perhaps the different “sets of avatars” correspond to five different baseline faces with parametric controls for dominance, trustworthiness, or both. Thus, the final images were created from these baseline faces by setting (i.e., controlling) these parametric controls, resulting in similar faces expected to be perceived differently based on the structural and expressive changes caused by the parametric control. Alternatively, perhaps avatars with pre-existing perceptual ratings were available and therefore they were not morphed but just used as-is. Please confirm what was done and revise the section for clarity. I’d also recommend replacing figure S1 with one showing a sample of faces from all five sets. Furthermore, if morphing was done, then I assume there was some sort of numerical input to set the face to a certain position on each dimension. If so, please explain what numerical values were selected and why the final Ns were 49, 175, 175, 175, and 175.

We would like to thank Reviewer 5 for pointing out the lack of clarity of our manuscript in the description of the training sets. In the revised version of the text, we explain in more details that the sets of avatar faces have been previously generated by Prof. Todorov's team and validated across multiple experiments conducted by various research teams. In addition, we more clearly present the five sets of avatars which corresponds to one face manipulated to present all the combinations of 7 levels of trustworthiness and 7 levels of dominance (corresponding to -3 to +3 standard deviations in Todorov and Oosterhof model; set 1), a set of 25 maximally distinct faces manipulated to either present these 7 levels of trustworthiness (set 2) or these 7 levels of dominance (set 3) and a set of 25 Caucasian faces manipulated to either present these same 7 levels of trustworthiness (set 4) or these same 7 levels of dominance (set 5). As suggested by Dr. Girard, we also updated Figure S1 in order to provide a clearer presentation of the avatars.

Lines 44-62:

> Each avatar is generated from an initial face and manipulated to either express a specific level of dominance, trustworthiness or both based on the model developed by Oosterhof & Todorov (1). These avatar faces have been shown to successfully elicit ratings of dominance and trustworthiness in participants (4–6). Thus, compared to participants' ratings on photographs that may be sensitive to the participants characteristics and to experimental protocol factors (such as the type of scale used to give the ratings), using avatars allow us to have well-validated sets of faces to train our model. These sets of avatars correspond to all existing and available validated avatars controlled for trustworthiness or dominance and generated by Facegen.
>
> More precisely, one set of avatars was generated from one single face and manipulated for both dominance and trustworthiness (*N* = 49; 7 levels of dominance and 7 levels of trustworthiness, each of the 7 levels corresponds to a standard deviation in Oosterhof and Todorov's (1) model ranging between -3 to +3 SD; set 1). Two other sets of faces correspond to 25 maximally distinct faces manipulated either on trustworthiness only (*N* = 175; 7 different levels of trustworthiness; set 2) or dominance only (*N* = 175; 7 different levels of dominance; set 3). Finally, the two last sets are composed of 25 Caucasian faces manipulated to present the same 7 levels of trustworthiness (*N* = 175; set 4) or of dominance (*N* = 175; set 5). Thus, three sets of avatars were used to build the model automatically extracting trustworthiness levels (sets 1, 2 and 4) and three were used to build the model automatically extracting dominance levels (sets 1, 3 and 5).

For each simulated face image, features were extracted using the OpenFace toolkit. Specifically, it seems that estimates of facial action units were used. If so, which action units? All of them or only those with a certain amount of variability? Were the binary classification results for action units used or the pseudo-continuous estimates of action unit intensity? Given that action units represent expressive motion and not structural differences, the models seem to be using only a subset of the information available to human perceivers. Using the positions of the facial landmark points, as well as the histogram of oriented gradients (HOG) features, provided by OpenFace in addition to the action units would overcome this limitation without requiring technical expertise in computer vision. Adding experiments with these features included would be interesting and may improve predictive performance. It would also lessen the reliance on OpenFace's ability to accurately detect low intensity, infrequent, and overlapping action units in data it wasn't trained on, which are all notoriously difficult tasks (and are not being validated here).

We would like to thank Dr. Girard for raising these different points. Because of the existence of some discrepancies between the binary and continuous estimations of action units by OpenFace, we used both measures in order to maximize the available information for our model. As the different avatar sets were generated using the same trustworthiness and dominance models, action units were filtered on their variance for discarding those who were infrequent or of very low intensity and thus of low informative value for our model. In the revised of the manuscript, we explain this methodological point. We decided to only use action units as a first step because of the well-known association between emotions and structural facial features used in first impressions, and especially in trustworthiness and dominance evaluations. However, we are aware of the potential of using richer features and it is our project to develop a home-built algorithm using landmarks to estimate perceived trustworthiness and dominance in portraits.

Lines 86-90:

> Because all our avatars were generated using the same models for trustworthiness and dominance, actions units with a variance inferior to 0.01 were discarded as not informative enough regarding cues of trustworthiness and dominance. The reason was that they were either too low in frequency or too low in intensity (ten action units discarded over thirty-three in both the trustworthiness and dominance avatar sets).

Based on the Ns reported, this dataset had a total of 399 simulated facial images – a small set for machine learning but probably adequate for relatively simple models like GLM, random forest, and linear SVM. However, I do wonder why the authors did not elect to train the models on data from both Facegen and the photograph-based "validation" datasets. An explicit rationale for this decision would be appreciated.

We would like to thank Dr. Girard for proposing this idea which was actually one of the options we considered before starting our study. However, real ratings on photographs are sensitive to the participant's sample characteristics and ratings on a same set of faces can vary from one participant sample to the other. Therefore, using real photographs for model optimization would make our algorithms highly sensitive to the participant sample from which we extracted the ratings. This is why we preferred using sets of avatars faces that have been shown to accurately elicit different levels of trustworthiness and dominance on different participant samples in order to extract the features that are consensually recognized as markers of trustworthiness and dominance. In the revised version of the manuscript, we added a sentence to explain this decision.

Lines 47-50:

> These avatar faces have been shown to successfully elicit ratings of dominance and trustworthiness in participants (4–6). Thus, compared to participants' ratings on photographs that may be sensitive to the participants characteristics and to experimental protocol factors (such as the type of scale used to give the ratings), using avatars allow us to have well-validated sets of faces to train our model.

The authors' dataset was then partitioned into a training set (80%) and testing set (20%). It is stated that "the percentage of avatars coming from each database was equal in the training and test samples for both trustworthiness and dominance" but the meaning of this was not clear as the word "database" had not been defined in this context/section. Perhaps the authors meant the sets of avatars?

We would like to thank Dr. Girard for pointing out this inconsistency, we replaced the word ‘database’ by ‘avatar set’ in the revised version of the manuscript.

Line 64-67:

> The total sample of avatar faces were then split in a training sample (80% of the faces) and a test sample (20% of the faces). Importantly, the percentage of avatars coming from each avatar set was equal in the training and test samples for both trustworthiness and dominance (Trustworthiness: $X^2(2) = 0.02$, $p > .250$; Dominance: $X^2(2) = 0.01$, $p > .250$).

Given that there were five databases and 1/5 of the data was used for testing, it seems that a leave-one-database-out evaluation would have made more sense. To the extent that there were dependencies and similarities within databases (e.g., if a morphing approach was used, then the faces would be quite similar within a database), this would have also been a more rigorous approach in that it would have estimated the algorithm’s ability to generalize to new databases (which can be challenging in many domains). I suppose the downside of this approach would have been that the databases were not equal in size. However, given that the authors used 20-fold cross-validation, they do not seem overly concerned with the size of the testing/evaluation sets. Some rationale for this decision would be good.

We would like to thank Dr. Girard for this proposition. However, we could not apply a leave-one-database-out procedure for optimizing our algorithms for two reasons. First the avatar set controlled for both dominance and trustworthiness is critical because these two features are correlated and thus faces controlled for these two traits simultaneously is necessary to train accurate models of trustworthiness and of dominance. Second each model is optimized on only 3 avatar sets – the 5 avatar sets correspond to the total number of avatar sets used for the optimization of the two models, a point we made clearer in the revised version of the manuscript. Thus a leave-one-database-out would imply to optimize on only 2/3 of the faces instead of 4/5 we used in for optimizing our models.

Lines 58-60:

> Thus, three sets of avatars were used to build the model automatically extracting trustworthiness levels (sets 1, 2 and 4) and three were used to build the model automatically extracting dominance levels (sets 1, 3 and 5).

More details about the implementation of the machine learning models are required for review and reproducibility. Were these models estimated in R, python, etc.? How were the hyperparameters optimized (e.g., grid search over what values)? How were the features selected? What family and link functions were selected for GLM? Why was a linear SVM selected (e.g., rather than an RBF kernel)?

We totally agree with Dr. Girard that we omitted several information in the presentation of our machine learning models. In the revised version of the manuscript, we specify that these models were estimated using the caret package of R and that the hyperparameters were optimized using a random search and that all the action units with a variance superior to 0.01 in our avatar sets (i.e., those with sufficient frequencies and intensities) were included in our models. We also that the GLM model used in our optimization is a linear model. Finally, a linear SVM was selected to be comparable to the GLM model but following Dr. Girard question we also included a RBF kernel SVM. This analysis revealed that the random forest

model was better than the RBF kernel SVM for trustworthiness modelling on all our fit indicators and similar to the RBF kernel SVM for the dominance modelling. This comparison was added to the revised version of the manuscript.

Lines 86-100:

As all our avatars were generated using the same models for trustworthiness and dominance, actions units with a variance inferior to 0.01 were discarded for not being informative on the features used as cues of trustworthiness and dominance either because of their very low frequency or only very low intensity (ten action units discarded over thirty-three in both the trustworthiness and dominance avatar sets). To determine which type of algorithm (linear model, random forest model from the *RandomForest* R package (7) - Breiman's random forest algorithm (8) - , or support vector model either linear or radial from the *kernlab* R package (9)) would provide the most accurate evaluations, we ran a repeated 20-folds cross-validation (five repetitions) on the training test of each of these models separately for dominance and trustworthiness using *caret* R package (10). Each model's hyperparameters were optimized using a random search. The hyperparameters optimized for each model are presented in Table S1. This analysis revealed significantly better performance for the random forest model than for the linear model and the linear SVM model in terms of mean absolute error, root square mean error and r-squared and was and better than, for the trustworthiness model, and similar to, for the dominance model, the radial SVM model (Table S1).

	**SVM linear**	**SVM radial**	**Random forest**	**Linear model**
**Hyperparameters**	*Cost (C)*	Cost (C) & sigma	*mtry*	ø
	Trustworthiness			
**Mean absolute error**	0.88 ± 0.02	**0.87 ± 0.02**	**0.82 ± 0.01**	0.87 ± 0.01
**Root mean squared deviation**	1.10 ± 0.02	**1.05 ± 0.02**	**0.99 ± 0.01**	1.06 ± 0.02
**R squared**	0.71 ± 0.01	**0.74 ± 0.01**	**0.78 ± 0.01**	0.72 ± 0.01
	Dominance			
**Mean absolute error**	0.92 ± 0.02	**0.79 ± 0.02**	**0.80 ± 0.01**	0.90 ± 0.02
**Root mean squared deviation**	1.14 ± 0.02	**0.99 ± 0.02**	**0.98 ± 0.02**	1.11 ± 0.02
**R squared**	0.68 ± 0.01	**0.76 ± 0.01**	**0.77 ± 0.01**	0.70 ± 0.01

Table S1. Model selection for extracting trustworthiness and dominance evaluations. Three indices of fit were computed, two which minimization indicates a better fit (mean absolute error and root mean squared deviation) and one which maximization indicates a better fit (R squared). The random forest was outperforming the linear model and the linear support vector model in the three indices of fit tested: mean absolute error, root mean squared deviation and r-squared. The random forest model was better than the radial support vector model for the trustworthiness model and similar to the radials support vector model for the dominance model. Values are presented as mean ± standard error to the mean.

In Table S1, clarify in the caption what the margins of error correspond to (e.g., SD, SE, or CI); it may also help some readers to note for each metric whether higher or lower numbers indicate better predictions.

We would like to thank Dr. Girard for this comment and this suggestion, in the revised version of the manuscript, we improved Table S1 accordingly.

Please clarify what is meant by "with a number of variables sampled as candidates at each split equals 9."

We realize the lack of clarity of this sentence which aimed at providing an intuitive understanding of the optimized hyperparameter of the random forest model. This sentence has been replaced in the revised version of the manuscript to be more understandable.

Lines 100-102:

> For both trustworthiness and dominance, the optimal m_{try} hyperparameter of the random forest models was found to be equal to 9, corresponding to setting the number of variables to consider at each tree to 9.

Figure S2 seems to be missing a caption and it was not described in text where these three different legend values came from (e.g., maximally distinct faces and Caucasian faces).

We would like to thank Dr. Girard for pointing out this issue, we corrected it in the revised version of the manuscript by presenting the three types of datasets in the Supplementary Information text.

Lines 53-60:

> More precisely, one set of avatars was generated from one single face and manipulated for both dominance and trustworthiness ($N = 49$; 7 levels of dominance and 7 levels of trustworthiness, each of the 7 levels corresponds to a standard deviation in Oosterhof and Todorov's (1) model ranging between -3 to +3 SD; set 1). Two other sets of faces correspond to 25 maximally distinct faces manipulated either on trustworthiness only ($N = 175$; 7 different levels of trustworthiness; set 2) or dominance only ($N = 175$; 7 different levels of dominance; set 3). Finally, the two last sets are composed of 25 Caucasian faces manipulated to present the same 7 levels of trustworthiness ($N = 175$; set 4) or of dominance ($N = 175$; set 5).

In describing the four different face databases (Karolinska, Oslo, Chicago, FEI), please provide the number of images provided in each; this is important for interpreting the significance of the correlations.

We agree with Dr. Girard that this important information was lacking and we added it in the revised version of the manuscript.

Lines 126-145:

> To assess the accuracy our trustworthiness and our dominance generator algorithm, we tested their predictions on four different face databases: the Karolinska database ($N = 70$ distinct faces) (11), the Oslo Face database ($N = 185$ distinct faces) (12), the Chicago database ($N = 520$ distinct faces) (13) and the FEI database ($N = 520$ distinct faces) (14). Given that our model was optimized on avatar faces, comparing our model's prediction to real participants ratings in a second step allows us to assess whether our model would give overall coherent ratings with those of real human beings. Our first analysis confirmed the significant correlation of the modeled trustworthiness and dominance estimates with the actual participants' ratings of trustworthiness and dominance ratings on the faces from these databases (except the FEI database which did not provide subjective ratings; Fig. S3). We found significant correlations for both trustworthiness and dominance estimates (trustworthiness: $r = .22$, $p < .001$, dominance: $r = .16$, $p < .001$ – $N = 768$ for each correlation, to not artificially increase the statistical power of this analysis only the

neutral and facing version of the faces were used for these correlations), confirming that our model gave trustworthiness and dominance estimates that are coherent with real participants' evaluations on these traits.

Figure S3 appears to be missing a caption and also visualization of the FEI database.

We would like to thank Dr. Girard for noticing that the absence of the FEI database on this figure may be confusing. This is due to the absence of subjective ratings on the pictures of the FEI database (the correlations between our automatically computed scores and participants' ratings were done on the three other databases). We corrected Figure S3 caption accordingly and we added this information when presenting the correlations.

Lines 138-141:

Our first analysis confirmed the significant correlation of the modeled trustworthiness and dominance estimates with the actual participants' ratings of trustworthiness and dominance ratings on the faces from these databases (except the FEI database which did not provide subjective ratings; Fig. S3).

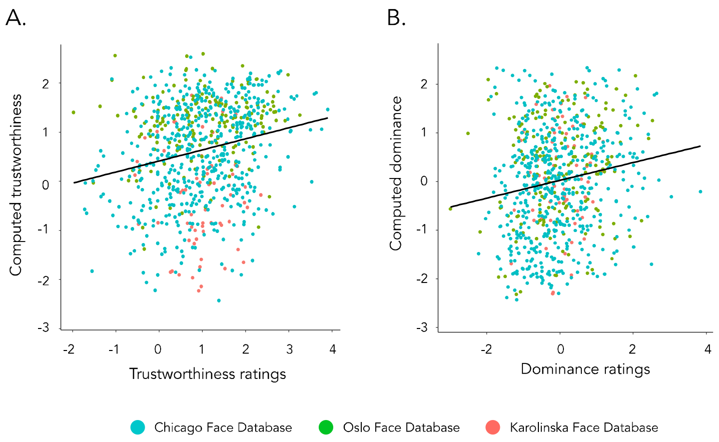

Fig. S3 Correlation between actual ratings of trustworthiness and dominance in the three databases providing subjective ratings of trustworthiness and dominance (the Chicago Face Database, the Oslo Face Database and the Karolinska Face Database) and the recovered trustworthiness and dominance levels using the Facial Action Units detected by Open Face and our random-forest model.

I would also say that correlation scores of 0.22 and 0.16 between predictions and human perceptions are pretty underwhelming. The fact that they are significantly different from zero is not very impressive given the apparent sample size. These results again make me wonder if a model with more training data (e.g., combining the avatar and photograph data) and more rich feature representations (e.g., including landmark positions and HOG features) would perform better. That said, I did find the ability to reproduce classical findings in social cognition to be more persuasive and was glad that those experiments were included.

We agree with Dr. Girard that the correlations between participants ratings and the extracted trustworthiness and dominance scores are quite small. However, ratings given by real participants are sensitive to the participant sample's characteristics (e.g., the proportion of female or the mean age of the sample) sometimes leading to lowly correlated ratings across participant samples. A model optimized on more data or using more features would not necessarily perform better. On the contrary, the well-known effects of gender, age and emotions as well as the stability of the evaluations across face positions were replicated by our models testifies its validity.

Although replicating gender and political party differences in portraits from Google is an interesting and indirect form of validation for the prediction models, I would like to see a subsample of paintings from the NPG and WGA rated for trustworthiness and dominance by human perceivers (e.g., on a web-based service like Mechanical Turk or Prolific) and directly compared to the algorithms' predictions. Good performance here (e.g., correlations above 0.5) would be very reassuring.

We would like to thank Dr. Girard for this suggestion. However, it has been shown that first impressions and face evaluations are highly sensitive to external features such as the clothes (see the recent paper by Oh, Shafir and Todorov of this very point). Thus, participants will certainly use these features in their evaluations if we present them paintings from the NPG and the WGA. Going one step further, even if we crop the faces to conduct such a study, participants could rely on cues such as the painting style to identify the period at which the portrait was paint which could influence their evaluations. This is actually why we decided to rely only on machine learning techniques which are blind to these external features and thus why it was important to be able to replicate classical social cognition findings on face evaluations with our algorithms.

Oh, D., Shafir, E., & Todorov, A. (2019). Economic status cues from clothes affect perceived competence from faces. *Nature Human Behaviour*, 1-7.

As a final point, stepping away from my methodological focus for a moment, I would agree with one of the other reviewers that it is important to consider whether the cues that indicate trustworthiness and dominance are stable over both time and culture. The authors make some argument about the apparent cultural stability of emotion and interpersonal perceptions (which should be noted is a controversial topic), but do not discuss the temporal stability piece. Without a time machine, this will be difficult to study, but it is worth mentioning as a limitation and discussion point that the same cues we would today perceive as trustworthy or dominant may have been interpreted in a different way when the portraits were created. This fact would make the use of an interpretable model more desirable, as we could at least be sure the current study was contributing a description of how facial appearance changed over time. However, I would not recommend that be explored here unless the authors also add some validation evidence regarding the action unit measures.

We agree with Dr. Girard that it is not properly speaking possible to test whether or not people from the past used the same facial cues as modern citizens to estimate trustworthiness and dominance in others. However, data showing the stability of the

facial features used to assess these traits both across individuals and cultures (notably, both European and non-European ones) suggests that the reliance of these facial cues is a consistent across the human species (see references in the main text). In particular, for dominance the very same facial features can be found in the non-human animal literature (e.g., Lefevre et al., 2014; Borgi & Majolo, 2016; Martin et al., 2019). It is more parsimonious to hypothesize that these very same facial features were used in the past in Europe to evaluate trustworthiness and dominance than hypothesizing that these cues have changed within this specific culture to converge across cultures in the modern period. However, we fully understand that this may be a concern for the readers of our paper and we strengthened this statement on this point in the revised version of the manuscript.

Borgi, M., & Majolo, B. (2016). Facial width-to-height ratio relates to dominance style in the genus Macaca. PeerJ, 4, e1775.

Lefevre, C. E., Wilson, V. A., Morton, F. B., Brosnan, S. F., Paukner, A., & Bates, T. C. (2014). Facial width-to-height ratio relates to alpha status and assertive personality in capuchin monkeys. PloS one, 9(4), e93369.

Martin, J. S., Staes, N., Weiss, A., Stevens, J. M. G., & Jaeggi, A. V. (2019). Facial width-to-height ratio is associated with agonistic and affiliative dominance in bonobos (Pan paniscus). Biology letters, 15(8), 20190232.

p. 3, lines 72-75:

> Experimental work have indeed revealed that specific facial features, such as a smiling mouth or wider eyes, are consistently recognized as cues of trustworthiness across individuals and cultures (17–22) and it is thus highly probable that the interpretation of these facial features have also been stable across time in Europe.

Reviewers' comments:

Reviewer #5 (Remarks to the Author):

The authors addressed most of my initial comments and added information that increases the clarity and reproducibility of the work. I have several comments after reading the rebuttal, all of which are focused on the question of measurement validation.

1. Given that the cross-validation procedure included the same base faces in both training and testing sets (and given that there were only three base faces total), the performance estimates from this procedure are likely overestimates of how well the system can generalize to new faces (which is the overall goal in this paper, not to mention generalizing from CGI avatar faces to portrait paintings). If the authors don't want to do an independent train-test split (or can't with their current dataset), then this should be acknowledged as a limitation of this piece of validity evidence.

2. The authors' response to my comment that the correlation scores (between algorithm predictions and human ratings on human photographs) were low did not really address the issue. Whether or not a model trained on more data and better features would perform better is an empirical question that the authors have the ability to explore, so saying that such a model "would not necessarily perform better" is a bit unsatisfying. Replicating well-known gender, age, and emotion effects provides relevant, indirect validity evidence but does not close the issue in a categorical sense as the response seems to imply. I defer to the editor to make the final decision about whether the provided validity evidence is sufficient for publication in this journal.

3. Similarly, I did not find very persuasive the authors' response to my comment that they should directly compare the algorithms' predictions to human ratings of trustworthiness and dominance on a sample of cropped faces from actual portrait paintings (as opposed to CGI avatar faces or human photographs). Even if painting style cues influenced human ratings to some degree (which to my knowledge has not been established), this would still be a much more direct form of validation than the other evidence provided (which also has limitations) and could be discussed in the context of such concerns and evaluated holistically with all the other evidence. It would also be relatively inexpensive and fast to conduct such an experiment online, so I will ask for this again (acknowledging that the editor may disagree and overrule me).

The authors addressed most of my initial comments and added information that increases the clarity and reproducibility of the work. I have several comments after reading the rebuttal, all of which are focused on the question of measurement validation.

1. Given that the cross-validation procedure included the same base faces in both training and testing sets (and given that there were only three base faces total), the performance estimates from this procedure are likely overestimates of how well the system can generalize to new faces (which is the overall goal in this paper, not to mention generalizing from CGI avatar faces to portrait paintings). If the authors don't want to do an independent train-test split (or can't with their current dataset), then this should be acknowledged as a limitation of this piece of validity evidence.

2. The authors' response to my comment that the correlation scores (between algorithm predictions and human ratings on human photographs) were low did not really address the issue. Whether or not a model trained on more data and better features would perform better is an empirical question that the authors have the ability to explore, so saying that such a model "would not necessarily perform better" is a bit unsatisfying. Replicating well-known gender, age, and emotion effects provides relevant, indirect validity evidence but does not close the issue in a categorical sense as the response seems to imply. I defer to the editor to make the final decision about whether the provided validity evidence is sufficient for publication in this journal.

We understand Reviewer 5's argument. In fact, we considered the methodological options suggested by Reviewers 5 when we started this project two years ago and concluded that they were unfit for our goals and dataset. In what follows, we unpack the decision-making process that led us to select specific methods. Note that all our methodological choices were then based on the current scientific consensus about first impressions and are very close to what others researchers in the field do, including A. Todorov.

First, it has been shown that face evaluations have a universal component that makes them stable across individuals, but also an idiosyncratic component that makes them vary from one individual to another (Hehman et al., 2017). For instance, some cues (such as the size of the eyes, or the shape of the lips) are universally used to assess trustworthiness (Todorov et al., 2013; Walker et al., 2011; Xu et al., 2012). However, individuals also rely on their own experience to evaluate these traits. As an example, individuals use their previous social experience to assess the trustworthiness of strangers (Dotsch et al., 2016). In our study, our goal was to design a model that would only extract universal cues and be, as much as possible, exempt of the idiosyncratic biases that associated with specific societies or cultures.

This is why we decided not to train a machine learning model on real faces rated by participants. The problem with using real faces rated by participants is that it would make the model highly dependent on the idiosyncratic biases of that specific participant pool unless the participant pool approximates universality. Achieving this pre-condition would require testing a participant pool going way beyond WEIRD participants recruited online. A recent paper by the Psychological Accelerator Project, on which one of the authors is a collaborator, demonstrates just how difficult that is: in order to obtain ratings from a "universal" sample, hundreds of researchers collected data in the context of an international collaboration (Jones

et al., 2018). We therefore favored an alternative method, which was to rely on avatars that are known to adequately capture universal dimensions. Todorov's avatars are perfect in that respect because they reliably elicit gradients of trustworthiness cross-culturally, a feature that explains why so many researchers in the field consider them to be the cleanest available stimuli (Todorov et al., 2013; Xu et al., 2012). Training our model on these avatars therefore appears to be the optimal solution.

Training the model on avatars, however, led us to apply a specific procedure to check the validity of the model. In our case, we were forced to use all the sets for training because each avatar set exposed the algorithm to different features and to use every avatar in each set (for a total of about 400 per model). This means that we had to assess validity on a completely different set of faces (real photographs of contemporary people retrieved from psychology databases and drawings, paintings and photographs retrieved from Google image). We believe that our validity procedure, therefore, is extremely conservative, more so than it would have been had we been able to restrict training to a subset of avatars.

Indeed, all our validity checks yielded robust results. First, the evaluations produced by our model on the photographs are correlated with those provided by human participants. More importantly, our model reproduces classical effects from social cognitive science: it rates younger, feminine, and happy faces as more trustworthy. This second test is of paramount importance because it indicates that our model produces the same biases as those systematically evidenced in human participants. We believe that these checks demonstrate that the model successfully generalizes to other datasets (including psychology databases and Google images) than the ones it was trained on, which provides a stronger proof of its validity than would a simple correlation with an additional set of avatars. In order to account for Reviewer 5's comment, which may be shared by other researchers in machine learning, we acknowledge the impossibility to use standard machine learning procedure and clearly explain the rationale of our method in the revised version of the manuscript:

Supplementary Materials, lines 108-116:

> This method differs from the classical train-test split used in machine learning which was not applicable given that each avatar of our dataset presented unique features in terms of luminance, texture and face shape which was important to increase the accuracy of our algorithms. However, our procedure is a highly conservative test of the validity of our models as the test set is completely different and independent of the training set. This conservative method for assessing the validity of the algorithms is particularly critical in the present study as our goal is to generalize the estimated trustworthiness and dominance evaluations to historical portraits, a completely different set of images than those classically used in social cognition research.

3. Similarly, I did not find very persuasive the authors' response to my comment that they should directly compare the algorithms' predictions to human ratings of trustworthiness and dominance on a sample of cropped faces from actual portrait paintings (as opposed to CGI avatar faces or human photographs). Even if painting style cues influenced human ratings to some degree (which to my knowledge has not been established), this would still be a much more direct form of validation than the other evidence provided (which also has limitations) and could be discussed in the context of such concerns and evaluated holistically with all the other evidence. It would also be relatively inexpensive and fast to conduct such an experiment online, so I will ask for this again (acknowledging that the editor may disagree and overrule me).

We understand Reviewer 5's comment. In fact, we considered this methodological option when we started this project two years ago and concluded that they were unfit for our goals and dataset. One major issue for psychologists working on first impressions in faces is that people are influenced by irrelevant cues surrounding the face (hairstyle, the presence or absence of glasses, etc.). Therefore, the standard is to use well-controlled photographs and, even more convincingly, avatar faces that are fully devoid of such cues.

This issue affects our stimuli even more strongly. What exactly would we be measuring if we ask online participants to provide their first impressions on historical portraits? It is unlikely that we would solely capture their first impressions. Rather, we'd most likely measure a combination of people's impression of the face, their impression of the historical period when they think the portrait was painted, their impression based on the social rank of the sitter suggested by their clothes, etc. (Oh et al., 2020).

A standard approach to this problem would be to crop the paintings to only display the face (a technique we routinely use in our lab). But even after cropping, there would be no way for us to hide the fact that the portrait was painted in the past, which means that participants' ratings would still capture both their impression of the face and of the historical period itself. Therefore, if online contemporary participants judged our historical stimuli as looking more trustworthy with time, we would have no way of knowing how much their judgment is confounded by other cues, e.g. they might perceive more recent portraits as more trustworthy just because they identify them as more recent, and thus as living in more trustworthy societies.

Another, slightly more sophisticated option, would be to use a software, such as FaceGen, to extract the facial features from the painting and remove all potential confounding cues by mapping them on avatars. This approach is extremely interesting but the technology simply isn't ready (see Figure).

The method we present in the paper adequately overcomes these challenges by training a model that analyzes the facial features of the portrait (and ignores all other cues). In addition to being unconfounded by non-relevant cues, this method also allows us to work on a much larger sample (over 3000 portraits in total), thereby increasing the reliability of our results. We acknowledge that the strength of our results will benefit from future replications, when better techniques become available. Yet, given our specific goal (recovering trustworthiness impressions from the past), and given the state of the art, we believe that we used the best available options, and more importantly that our methodology has a high degree of validity.

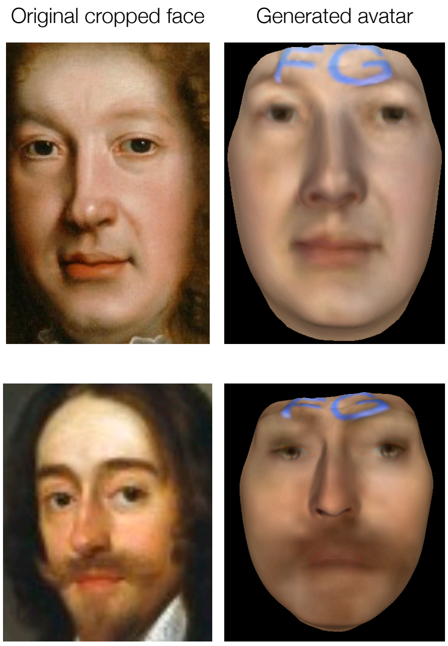

Figure – Example of generated avatar faces from historical portraits (National Portrait Gallery)
Softwares such as FaceGen propose to automatically create avatar faces from pictures. However, while this process is efficient for some portraits (such as the portrait of John Dryden painted in 1668, top) for others, the methods is perfectible (such as the portrait of King Charles I painted in 1631) making this option inappropriate to study the evolution of portraits for the moment.

References

Dotsch, R., Hassin, R. R., & Todorov, A. (2016). Statistical learning shapes face evaluation. *Nature Human Behaviour*, *1*(1), 1-6. https://doi.org/10.1038/s41562-016-0001

Hehman, E., Sutherland, C. A. M., Flake, J. K., & Slepian, M. L. (2017). The unique contributions of perceiver and target characteristics in person perception. *Journal of Personality and Social Psychology*, *113*(4), 513-529. https://doi.org/10.1037/pspa0000090

Jones, B. C., DeBruine, L. M., Flake, J. K., Liuzza, M. T., Antfolk, J., Arinze, N. C., Ndukaihe, I. L. G., Bloxsom, N., Lewis, S., Foroni, F., Willis, M., Cubillas, C. P., Vadillo, M. A., gilead, michael, Simchon, A., Saribay, S. A., Owsley, N. C., Calvillo, D. P., Włodarczyk, A., … Coles, N. A. (2018). *To Which World Regions Does the Valence-Dominance Model of Social Perception Apply?* [Preprint]. PsyArXiv. https://doi.org/10.31234/osf.io/n26dy

Oh, D., Shafir, E., & Todorov, A. (2020). Economic status cues from clothes affect perceived competence from faces. *Nature Human Behaviour*, *4*(3), 287-293. https://doi.org/10.1038/s41562-019-0782-4

Todorov, A., Dotsch, R., Porter, J. M., Oosterhof, N. N., & Falvello, V. B. (2013). Validation of data-driven computational models of social perception of faces. *Emotion*, *13*(4), 724-738. https://doi.org/10.1037/a0032335

Walker, M., Jiang, F., Vetter, T., & Sczesny, S. (2011). Universals and Cultural Differences in Forming Personality Trait Judgments From Faces. *Social Psychological and Personality Science*, *2*(6), 609-617. https://doi.org/10.1177/1948550611402519

Xu, F., Wu, D., Toriyama, R., Ma, F., Itakura, S., & Lee, K. (2012). Similarities and Differences in Chinese and Caucasian Adults' Use of Facial Cues for Trustworthiness Judgments. *PLOS ONE*, *7*(4), e34859. https://doi.org/10.1371/journal.pone.0034859

***REVIEWERS' COMMENTS:

Reviewer #5 (Remarks to the Author):

The added text regarding my first comment about cross-validation is fine. The cross-validation results may be overestimates of generalizability to new domains but the authors did include the other validation results as well to explore generalizability to other domains. I'm not sure that these are "extremely conservative" estimates of how well the model will generalize to historical portraits as the authors stated in the rebuttal, but I think the phrasing in the revision is fine. Certainly, I too would like more people in the field to do external validations.

The authors' rationale for not training models either on photographs only or on avatars and photographs is also fine. I think this would be an interesting experiment and am not ready to rule out the possibility that this approach may yield better predictive performance than the avatar only model, but I defer to the authors as experts in this area. If they feel strongly there are good reasons not to do this, then I accept that.

My only remaining concern is the final one. This leads me to two questions:

First, would it add *relevant* information to know how correlated the algorithm's predictions of trustworthiness are with human judgments of trustworthiness in the specific domain of historical portraits? Even after reading the rebuttal, I think the answer here is yes. The algorithm was ostensibly trained on human judgments and human judgments were used to evaluate it in the context of avatars and photographs. The same could be done with historical portraits. If there are concerns about setting and clothing, then crop the face out. If there are concerns about time-related cues (e.g., painting and hair style), then model this (e.g., report the partial correlation between human judgments and algorithmic predictions, controlling for time). As I stated in my original comment, I agree that this approach would have some limitations, but so do the other approaches the authors used. I was and still am suggesting that these different approaches are complementary and could all be synthesized and discussed holistically. I agree with the author's conclusion that the FaceGen solution is not yet ready and that this will be a great direction for future research whenever it is ready.

Second, would it add *necessary* information to know how correlated the algorithm's predictions of trustworthiness are with human judgements of trustworthiness in the specific domain of historical portraits? I'm less sure about this. I think it would strengthen the paper and could see some readers wanting this (especially in a premier outlet like this and especially given the modest correlation of 0.22 in the photograph data). That said, the authors do provide some portrait-specific validation in the form of replications of the gender and age effects using the algorithm in the National Portrait Gallery data. So I'm open to the compromise of the authors adding a bit of text to the supplemental materials write-up about the pros and cons that would come from using my suggested validation approach (and other alternatives, like the FaceGen one they found to not be ready) and an explanation for why they decided not to go this route. I just ask them to try hard to provide a balanced view of this approach (i.e., not to

undersell its pros and oversell its cons).

To keep the publication process moving, I am recommending acceptance with the request that the authors add the text just described.
