# Supplementary material for: Author Correction: Tracking historical changes in perceived trustworthiness in Western Europe using machine learning analyses of facial cues in paintings
Source: Nat Commun. 2022 Sep 14;13:5379. doi: 10.1038/s41467-022-31843-x (PMC9474442; doi:10.1038/s41467-022-31843-x)
Supplement: Supplementary file 3 — Supplementary Information 3 [file 41467_2022_31843_MOESM3_ESM.docx]

Dear Dr Safra & Dr. Baumard,

We have compiled a list of issues (appended below) that we think would benefit from further clarification. Some of these issues are those that emerged from the recent social media exchanges, and do not reflect our own views, however they should be addressed for the benefit of the readers.

Please could you respond to these in a point-by-point fashion, and clearly state what modifications, if any, you are prepared to include from our requests, as well as other suggestions you have for addressing these points? We would hope to receive these changes within 1-2 weeks.

As we had previously discussed, we may also decide to involve the existing and/or new reviewers in the assessment of your responses. We also believe that it would be beneficial to publish these additional reports, together with the pre-publication reports.

Please let us know if you have any further questions about this request.

Best regards,

***

1. The present wording of the title and areas of the main text suggest that the algorithm measures the actual trustworthiness of individuals based on their facial characteristics. Some of our readers have linked this to physiognomy which has been discarded as pseudoscience. We understand that the algorithm instead measures the trustworthiness of the subject as perceived by contemporary humans, i.e. those against whom the algorithm was calibrated. Please explain areas of the manuscript that can be edited to clarify this point.

2. Related to the misunderstanding above regarding actual versus perceived trust ratings, readers have raised concerns about the potential public misuse of the algorithm for identifying individuals who are versus aren’t trustworthy (and further linking this to levels of affluence). There were also related concerns that this algorithm may lead to biases against particular groups, given that it is based on the existing biases humans have in evaluating the trustworthiness of faces. We would strongly encourage you to include a disclaimer, explaining that the algorithm was not built for this purpose, and that it cannot provide accurate information about the actual trustworthiness levels of individuals.

3. Correlation effects such as those evidenced in Suppl. Fig. 3 are not clearly described. The plot seems to suggest that the data has been fit linearly to produce a trend line, if so the reliability of such fit is not stated. Please clarify the analytical methods used to extract all correlations throughout to fit the data. We may ask our reviewers to reassess the appropriateness of your analytical approach.

4. Some of our readers raised concerns about the small effect sizes. We understand that small effects are to be expected, based on the approach used and the dataset available, for answering this research question. However, it would be helpful to readers if these points were explicitly made. Please acknowledge the size of the effects, and provide this context.

5. There were concerns raised regarding the limitations of relying on a curated set of paintings of (primarily) Western European portraits. For example, it would be inappropriate to imply that these relationships extend to the larger population of the time (or extend past what might be assumed to be a relatively elite, wealthy population represented in the portraits used--a detail that may be especially relevant to consider when drawing conclusions about relationships to broader social and economic development). We would strongly encourage you to state potential generalizability issues.

6. Some of our readers have raised the concern that the algorithm is biased, given the use of predominantly Caucasian faces in the training set. It is not clearly stated in the manuscript how varied the ethnicities of the avatars are, what systematic bias this might produce, and how it would affect the interpretation of the results. Please clarify these points. In doing so, it would also help to clarify the ethnicity distribution of the human ratings against which the algorithm was validated.

7. Some readers have criticised the use of GDP as a measure of affluence, noting that when applied to pre-capitalist societies GDP is unable to describe economic progress or living standards of the entire population. We understand that a similar point was raised in review. Please justify your choice of GDP, discuss its limitations, and include in the manuscript and/or SI the other analyses you conducted that speak to this issue.

8. Some readers have noted that it is not possible to know if the facial cues we currently rely on to evaluate trustworthiness based on faces (and which are built into the algorithm) are the same as those used in the past. This was a point that was raised in review, and the limitations discussed there should be clarified in the main manuscript text.

**Response**

In what follows, we provide detailed responses to the clarification questions raised by the editors. In addition to the suggested modifications below, we would like the full review history to be made public in a spirit of full transparency.

***1. The present wording of the title and areas of the main text suggest that the algorithm measures the actual trustworthiness of individuals based on their facial characteristics. Some of our readers have linked this to physiognomy which has been discarded as pseudoscience. We understand that the algorithm instead measures the trustworthiness of the subject as perceived by contemporary humans, i.e. those against whom the algorithm was calibrated. Please explain areas of the manuscript that can be edited to clarify this point.***

It is indeed of paramount importance that there is no possible misunderstanding on this point. We would like to make all necessary changes to ensure we are unambiguous throughout the paper.

The readers are right that some face research is about establishing a link between perceived and actual trustworthiness (e.g., Little et al., 2013) but our study isn’t about this. Our study is solely about self-presentation in portraits and *perceived* trustworthiness. In fact, we fully agree that faces are no reliable indicator of character and we make no claim whatsoever about individuals' actual trustworthiness. In order to make this idea clearer, we would like to request a number of changes.

First, we would like the title of the manuscript to be changed. In the original title and in other places in the manuscript, we had intended “trustworthiness” as a shorthand to refer to our measure of “perceived trustworthiness”. We recognize this was a source of much confusion and apologize for the misconceptions this has generated:

‘Tracking historical changes in trustworthiness displays in Western Europe using machine learning analyses of facial cues in painting’.

Second, we would also like to modify the abstract (modifications appear in bold):

Social trust is linked to a host of positive societal outcomes, including improved economic performance, lower crime rates and more inclusive institutions. Yet, the origins of trust remain elusive, partly because social trust is difficult to document in time. Building on recent advances in social cognition, we design an algorithm to automatically generate **ratings** **of perceived** trustworthiness for the facial action units (smile, eye brows, etc.) of European portraits in large historical databases. Our results show that trustworthiness **displays** in portraits increased over the period 1500 – 2000 paralleling the decline of interpersonal violence and the rise of democratic values observed in Western Europe. Further analyses suggest that this rise of trustworthiness displays is associated with increased living standards.

Third, we realised that we had neglected to ensure that every occurence of 'trustworthiness' came with an unambiguous qualifier. In doing so, we erred too far on the side of readability and streamlined rhetoric. We realise now that clarity on this point is absolutely paramount.

found that some occurrences of the word “trustworthiness” in our paper did not come along with a qualifying word unambiguously stating that we are referring to perceived trustworthiness (e.g., either ‘displays’, ‘perceived’, ‘appearing’ or ‘signaling’). We would like to disambiguate these occurrences.

We believe that these three revisions will avoid further misconceptions. To be very clear, we are interested in what people display in their portraits: by choosing a specific pose, for instance, the same sitter will *appear* more dominant, more trustworthy, or more kind (Todorov et al., 2014). On the other hand, depending on the social context or on the sitter’s specific intentions, the sitter will want to *display* different traits. This does not say anything about their true character. In fact, there is evidence that one image of the very same person can trigger different first impression ratings (Todorov et al., 2014) and that cues of perceived trustworthiness can be largely *counterbalanced* by specific poses and muscle activations (Gill et al., 2014). This is why our algorithm is based on the detection of the activation of Facial Action Units. In other words, our algorithm aims to detect facial muscles activations, and *not* fixed facial features which are not under the individual’s control.

References

Little, A. C., Jones, B. C., DeBruine, L. M., & Dunbar, R. I. (2013). Accuracy in discrimination of self-reported cooperators using static facial information. *Personality and Individual Differences*, *54*(4), 507-512.

Gill, D., Garrod, O. G., Jack, R. E., & Schyns, P. G. (2014). Facial movements strategically camouflage involuntary social signals of face morphology. *Psychological science*, *25*(5), 1079-1086.

Todorov, A., & Porter, J. "Misleading first impressions: Different for different facial images of the same person." *Psychological science* 25.7 (2014): 1404-1417.

***2. Related to the misunderstanding above regarding actual versus perceived trust ratings, readers have raised concerns about the potential public misuse of the algorithm for identifying individuals who are versus aren’t trustworthy (and further linking this to levels of affluence). There were also related concerns that this algorithm may lead to biases against particular groups, given that it is based on the existing biases humans have in evaluating the trustworthiness of faces. We would strongly encourage you to include a disclaimer, explaining that the algorithm was not built for this purpose, and that it cannot provide accurate information about the actual trustworthiness levels of individuals.***

To avoid the potential misuse of the developed algorithm, we have already restricted access to the algorithm. It is only available upon request and only for research and education purposes. In addition, we would like to add the following disclaimer to the main text (please note that we have already added this disclaimer on the Open Science Framework repository:

**Disclaimer:** The present algorithm was built to mimic participants’ ratings of *perceived* trustworthiness on the processed images based on a set of facial muscles activations. The scores computed by the algorithm approximate real participants’ subjective evaluations of trustworthiness on the processed images based on the pose taken by the individual. It provides no meaningful information whatsoever about individuals’ *actual* trustworthiness.

We deeply care about developing an algorithm that is not biased against specific groups. We address concerns regarding these potential biases in response to point 6.

***3. Correlation effects such as those evidenced in Suppl. Fig. 3 are not clearly described. The plot seems to suggest that the data has been fit linearly to produce a trend line, if so the reliability of such fit is not stated. Please clarify the analytical methods used to extract all correlations throughout to fit the data. We may ask our reviewers to reassess the appropriateness of your analytical approach.***

For each analysis presented in the paper, we provide reliability values indicated as either z-values, r correlation coefficients for correlations and t-values for Student’s t-tests. In the version of the manuscript that was evaluated by the five reviewers, the supplementary figures were presented alongside the methods section describing the analyses, which may have facilitated the direct matching between text and figures. In order to make things clearer for the readers, we propose to add details about the specific tests we conducted directly in the caption of the figures.

For example:

**Supplementary Figure 3** Correlation between participants’ ratings of trustworthiness and dominance in the three databases providing subjective ratings of perceived trustworthiness and perceived dominance (the Chicago Face Database, the Oslo Face Database and the Karolinska Face Database) and the perceived trustworthiness (**A**, Pearson correlation: *r* = .22, t(768) = 6.19, p < .001) and perceived dominance (**B**, Pearson correlation: *r* = .16, t(769) = 4.54, p < .001) levels estimated using the Facial Action Units detected by Open Face and our random-forest model. Source data are provided as raw data and scripts on the online depository.

Concerning our analytical approach, we chose to conduct Pearson’s correlations to assess the coherence of our algorithm’s output with first impressions provided by real participants. More precisely, the goal of this first analysis was to assess whether pictures that received higher evaluations of perceived trustworthiness by real participants were rated as higher on perceived trustworthiness by our algorithm. A perfect correspondence between the estimations generated by our algorithm and those generated by real participants would lead to a perfect linear relation. Pearson correlations are therefore appropriate to assess the existence of a *linear* relation between these two measures. Similarly, we performed Pearson correlations to estimate the correspondence between the output of our algorithm for front and profile views for the same faces. Here again, a perfect correspondence would have led to a perfect linear relation between these two values, which makes Pearson correlations appropriate. The last correlation we conducted estimates the relation between estimations of faces’ age by real participants and the estimations of perceived trustworthiness made by these participants and by our model. We conducted this analysis once again by running a Pearson correlation as we expect these two variables to covary linearly.

Next, we ran Student t-tests to compare the output of our model when processed on images of men and women, as well as of Democrat and Republican representatives. This is the standard approach to compare between-group differences on quantitative values for large samples.

In addition, the effect of emotion on the output of our model was estimated using a two-level regression, first estimating the slope of the emotion effect on each image and testing the significance of this effect against zero at the group level. As the above-mentioned statistical methods, this method is classically used when dealing with repeated measures.

Finally, we analyzed the output of our algorithm using linear regressions taking each picture as an individual data point. This analytical strategy was further explained in the Methods section of the manuscript:

Selfie City database analysis:

Extracted perceived trustworthiness was analyzed using a linear model taking the sitter’s gender, the city longitude and latitude and the sitter’s perceived dominance as control variables.

National Portrait database analyses:

In order to keep a maximal temporal resolution, missing values in the GDP per capita and Polity2 indices were completed using the closest previous value, except for the time-lag analyses in which no imputation was made. A total of 1943 data points were included in the analyses looking at the effect of GDP per capita. A total of 1115 data points were included in the analyses looking at the effect of Polity2. Paintings were analyzed using individual linear models (each painting corresponding to one data point), taking the sitter’s gender, age and level of perceived dominance as control variables. Bayes factor analyses were conducted using the BIC approximation, which approximates Bayes factors computed under the unit information prior ^63^.

Finally, time-lag analyses were conducted to analyze the temporal dynamics between trustworthiness displays, GDP per capita and democratization. To do so, data were averaged by decades and analyzed at the aggregated level. The model on trustworthiness displays at decade *d* included the simultaneous level of perceived dominance at decade *d*, the linear effect of the time, the delayed levels of perceived trustworthiness and perceived dominance at *d-2*, and the level of GDP per capita or democratization at *d-2*. On the other hand, models of GDP per capita or democratization included the linear effect of time, the delayed level of GDP at *d-2* and the delayed levels of perceived trustworthiness and of perceived dominance at *d-2*. For each variable, the model with the delayed variable of interest (GDP per capita or democratization for the perceived trustworthiness models, and perceived trustworthiness for the models on GDP per capita and democratization) were compared with the models in which this variable was removed. Finally, in order to assess the robustness of our effects, we also tested the same models with a delay of one decade instead of two decades (Supplementary Table 3).

Web Gallery of art database analyses:

As for the National Portrait Gallery, the missing levels of affluence and democratization were completed using the previous complete value. The same models as previously were used except that a random effect was included to take the localization of the paintings into account. This resulted, for the analysis of the effect of GDP per capita and democratization in two-level mixed models, taking each painting as an individual data point clustered by the country of production. Correspondingly, for time-lag analyses, we use two-level mixed models but with data aggregated by decades.

Finally, we would like to further indicate that all the analyses scripts and the scripts used to produce the figures have been openly accessible on the Open Science Framework repository linked to the paper.

***4. Some of our readers raised concerns about the small effect sizes. We understand that small effects are to be expected, based on the approach used and the dataset available, for answering this research question. However, it would be helpful to readers if these points were explicitly made. Please acknowledge the size of the effects, and provide this context.***

We understand that the size of some of the effects, especially the correlation between the output of our algorithm and real participants’ evaluations may appear small for readers outside the field of first impressions from faces. But these effect sizes are in the expected ballpark, as noticed by Prof. Todorov in his review of our manuscript (which we would like to make public): ‘*The correlations were not generally high, but this is to be expected as the FaceGen faces/ modeling doesn't capture face texture well and the latter is very important for judgments.*’

In addition, our algorithm was trained to generate ratings of perceived trustworthiness based on the facial features that are commonly used to produce these evaluations. In other words, our algorithm was not trained to *mimic* the evaluations of a specific pool of participants (who will each have their own idiosyncratic biases). Indeed, individuals notably rely on idiosyncratic features when forming a first impression on a new face. This is a point that we discussed with Reviewer 5 and copy below:

‘*We agree with Dr. Girard that the correlations between participants ratings and the extracted trustworthiness and dominance scores are quite small. However, ratings given by real participants are sensitive to the participant sample’s characteristics (e.g., the proportion of female or the mean age of the sample) sometimes leading to lowly correlated ratings across participant samples. A model optimized on more data or using more features would not necessarily perform better. On the contrary, the well-known effects of gender, age and emotions as well as the stability of the evaluations across face positions were replicated by our models testifies its validity.* ‘

‘*First, it has been shown that face evaluations have a universal component that makes them stable across individuals, but also an idiosyncratic component that makes them vary from one individual to another (Hehman et al., 2017). For instance, some cues (such as the size of the eyes, or the shape of the lips) are universally used to assess trustworthiness (Todorov et al., 2013; Walker et al., 2011; Xu et al., 2012). However, individuals also rely on their own experience to evaluate these traits. As an example, individuals use their previous social experience to assess the trustworthiness of strangers (Dotsch et al., 2016). In our study, our goal was to design a model that would only extract universal cues and be, as much as possible, exempt of the idiosyncratic biases that associated with specific societies or cultures.*‘

Therefore, this analysis was only conducted to assess whether our algorithm’s output was broadly aligned with real participants’ ratings. We found that it was the case. Concerning the other validation analyses, whenever it was possible, we reported the effects that have been found in the ratings provided by real participants. In both cases, the effect sizes obtained when analyzing the output of our model were coherent with the analysis conducted on real participants’ ratings.

Finally, we would like to point out that we followed the latest APA recommendations for reporting statistics and reported exact statistical results, which is more accurate than relying on qualitative rule-of-thumbs (as it used to be the case for p-values). We believe this gives the informed readership of *Nature Communication* all the information needed to critically read our manuscript and interpret our results. In addition, we provided an open access to the analysis scripts and datasets on the Open Science Framework repository linked to the manuscript. We believe publishing the full review history of our paper will alleviate the readers’ concerns but we are happy to also include further details in the main text if relevant.

***5. There were concerns raised regarding the limitations of relying on a curated set of paintings of (primarily) Western European portraits. For example, it would be inappropriate to imply that these relationships extend to the larger population of the time (or extend past what might be assumed to be a relatively elite, wealthy population represented in the portraits used--a detail that may be especially relevant to consider when drawing conclusions about relationships to broader social and economic development). We would strongly encourage you to state potential generalizability issues.***

We totally agree that, although they have been carefully chosen for being diverse and, for the National Portrait Gallery, for its purpose of documenting English historical figures, our results are restricted to the analyzed datasets. It is possible that the evolution we study is limited to the wealthiest European Western people. In fact, there is evidence that there is a socioeconomic gradient of general social trust in contemporary populations and that the social attitudes measured in people living in deprived environments are different from those measured in people living in more affluent environment (Zwirner & Raihani, 2020; Pepper & Nettle, 2017; Brandt et al., 2015; Holland et al., 2011; Korndörfer et al., 2015; Levine et al., 2008).

In addition to the geographical restriction of our findings, which is now stated in the proposed new title, we would like to add the following paragraph at the end of the discussion:

Finally, several limitations are to be noted. First, one cannot assume that the evolution of trustworthiness displays depicted in this study extends to the larger population of the period. In fact, there is evidence that there is a socioeconomic gradient of general social trust in contemporary populations and that social attitudes measured in people living in deprived environment are different from those measured in people living in more affluent environments (Zwirner & Raihani, 2020; Pepper & Nettle, 2017; Brandt et al., 2015; Holland et al., 2011; Korndörfer et al., 2015; Levine et al., 2008;). Thus, the phenomenon described in this article might be limited to the relatively elite, wealthy population represented in the portraits. (...)

References

Brandt, M. J., Wetherell, G., & Henry, P. J. (2015). Changes in income predict change in social trust : A longitudinal analysis. *Political Psychology*, *36*(6), 761–768.

Holland, Jo, Antonio S. Silva, and Ruth Mace. Lost letter measure of variation in altruistic behaviour in 20 neighbourhoods. *PloS one* 7.8 (2012): e43294.

Korndörfer, Martin, Boris Egloff, and Stefan C. Schmukle. A large scale test of the effect of social class on prosocial behavior. *PloS one* 10.7 (2015): e0133193.

Levine, Robert V., Stephen Reysen, and Ellen Ganz. "The kindness of strangers revisited: A comparison of 24 US cities." *Social indicators research* 85.3 (2008): 461-481.

Pepper, G., & Nettle, D. (2017). The behavioural constellation of deprivation: Causes and consequences. *Behavioral and Brain Sciences*, *40*, e314.

Zwirner, E., & Raihani, N. (2020). Neighbourhood wealth, not urbanicity, predicts prosociality towards strangers. *Proceedings of the Royal Society B*, *287*(1936), 20201359.

***Editor 6. Some of our readers have raised the concern that the algorithm is biased, given the use of predominantly Caucasian faces in the training set. It is not clearly stated in the manuscript how varied the ethnicities of the avatars are, what systematic bias this might produce, and how it would affect the interpretation of the results. Please clarify these points. In doing so, it would also help to clarify the ethnicity distribution of the human ratings against which the algorithm was validated.***

Developing an algorithm that was not biased against any particular group was of central importance when we conducted this study. As explained in the Methods section, we trained our algorithm on all the existing validated avatars available on Prof. Todorov’s website. These avatars have been generated to represent different ethnicities. All the avatars that were included in the model’s optimization process are openly accessible on the Open Science Framework depository linked to the manuscript.

Similarly, the algorithm was validated on samples of pictures that are diverse in terms of ethnicities. In particular, the Chicago Face Database, on which we validated our algorithm, has been created to be ethnically diverse. Additional analyses confirmed that the perceived trustworthiness scores generated by our algorithm were not significantly higher for Caucasian faces than for any other ethnicity. In addition, the ratings against which we validated our algorithm were provided by participant samples with ethnic diversity, as explained in the original article presenting the Chicago Face Database: ‘*A convenience sample of 1,087 raters made subjective ratings of the standardized neutral faces. Participants included 552 females and 308 males (227 did not report) and came from diverse racial backgrounds (516 White, 117 Asian, 74 Black, 72 biracial or multiracial, 57 Latino, 18 other, and 233 did not report).*’ This information is fully available in the original paper describing the database (Ma et al., 2015).

We would like to add the following clarifications to Methods section of the main text:

Taken together, these databases are ethnically diverse. Given that our model was optimized on avatar faces, comparing our model’s prediction to real participants' ratings in a second step allows us to assess whether our model would give ratings that are coherent overall with those of real human beings, notably of an ethnically diverse participants sample (Ma et al., 2015).

As for the avatars used in the optimization process, the list of the faces included in our validation process is openly accessible on the Open Science Framework repository linked to the project.

References

Ma, D. S., Correll, J., & Wittenbrink, B. (2015). The Chicago face database: A free stimulus set of faces and norming data. *Behavior research methods*, 47(4), 1122-1135.

***Editor 7. Some readers have criticised the use of GDP as a measure of affluence, noting that when applied to pre-capitalist societies GDP is unable to describe economic progress or living standards of the entire population. We understand that a similar point was raised in review. Please justify your choice of GDP, discuss its limitations, and include in the manuscript and/or SI the other analyses you conducted that speak to this issue.***

The use of GDP per capita is a standard way to measure economic development in economic history. To give an idea of how standard such a measure is, one can consult classical papers in economic history in which GDP before 1800 is quantified: Maddison *The world economy* (2007, 7243 citations), Maddison, *Contours of the world economy* (2007, 1716 citations), Broadberry et al., *British economic growth, 1270–1870* (2015, 525 citations), Broadberry & Gupta, *The early modern great divergence: wages, prices and economic development in Europe and Asia, 1500–1800* (2006, 499 citations), etc. There are now GDP times series for England (Broadberry, Campbell, et al., 2015), Italy (Malanima, 2011), Belgium (Buyst, 2011), The Netherlands (Van Zanden & Van Leeuwen, 2012), Germany (Pfister, 2011), Poland (Malinowski & van Zanden, 2017), China (Broadberry et al., 2018), Japan (Bassino et al., 2019), Iraq (Pamuk & Shatzmiller, 2014), Sweden (Schön & Krantz, 2012), India (Broadberry, Custodis, et al., 2015). These times series are discussed and regularly updated, notably through the Maddison Project Database hosted by the University of Gröningen (https://www.rug.nl/ggdc).

Likewise, the idea that medieval and early modern societies (or non-Western societies) are 'pre-capitalist' societies has been debunked a long time ago. As historian of English medieval economy Jim Galloway writes: '*Few would now doubt the pervasive influence of money and monetary equivalences, or the impact of the market upon the organisation of production as well as distribution, from at least as early as the thirteenth century.*' (Galloway, 2000). Many empirical papers have tried to quantify the extent to which there was some market integration in pre-industrial societies. To give just one example, using information of grain yields and prices at 227 different locations, Clark (2015) shows that *'the most important of all markets, that for grain, was nevertheless both extensive and relatively efficient as early as 1209.*' He adds: *'Whatever the rhetoric of medieval law and social thought the real effect of constraints on the operation the grain market seems to have been minimal. England had an elaborate market economy at least 500 years before it had sustained economic growth.*'

In fact, the very reason why it is possible to reconstruct GDP in medieval and early modern societies is precisely because, there are data about prices, yields per acre, wages, agricultural and industrial production and urbanization. This last point means that GDP per capita is not the only tool that allows economic historians to quantify the evolution of living standards in history. Allen, for instance, has produced long time series about the evolution of wages in Western Europe that suggest that English and Dutch workers were better off than their European counterparts during the early modern period (Allen, 2001). Another standard tool in economic history, although less accurate, is urbanization rate (Acemoglu et al., 2002; De Long & Shleifer, 1993). Urbanization rate is well correlated with economic development (Jedwab & Vollrath, 2015) because it tracks agricultural surplus, and often easier to measure (Bosker et al., 2013). Yet another tool is the book production per capita (Buringh & Van Zanden, 2009). Books production is linked to a number of variables used in growth theories (in particular human capital). Moreover, they are easy to track and quantify in the long-run. They have been shown to be well correlated with economic development (Buringh & Van Zanden, 2009).

To conclude, we agree that GDP per capita is not perfect and it is actually not the only tool to measure economic progress. We used it because it is the most standard tool to quantify economic development. However, the same analyses can be performed with other indicators such as book production. In fact, we presented such results during the review process in response to Reviewer 2:

‘*We would like to thank Dr. Mikucka for this remark. In order to assess the robustness of our results to the use of different measures of affluence, we conducted additional analyses with two other predictors of living conditions: the number of book titles per capita, which is a validated proxy of affluence (CIT) and the presence of an armed conflict (either internal or international, as suggested by Dr. Mikucka). The effect of affluence on trustworthiness displays was replicated using number of book titles per 1000 capita (which is another yet less sensitive proxy of affluence) in both the National Portrait Gallery and the Web Gallery of Art databases (see table below). However, no significant effect of armed conflicts was evidenced. This result may be explained by the fact that armed conflicts are not monotonously linked to affluence (wars can start in periods of poverty or of affluence) and that we could not find a database on the intensity of armed conflicts. We included the effect of book titles per capita as a supplementary analysis in the revised version of the manuscript.*’

These results had been taken out of the SI later on during the review process. We have put them back in the SI.

We also propose to add the following sentence to the final paragraph:

Finally, several limitations are to be noted. (...) Third, times series of GDP per capita are only estimates. They do not fully capture the evolution of living standards and inequalities. There is no doubt that they will be improved by future work, and that our results will need to be tested again with more accurate estimates.

References

Acemoglu, D., Johnson, S., & Robinson, J. A. (2002). *The rise of Europe : Atlantic trade, institutional change and economic growth*. National Bureau of Economic Research Cambridge, Mass., USA.

Allen, R. C. (2001). The great divergence in European wages and prices from the Middle Ages to the First World War. *Explorations in economic history*, *38*(4), 411–447.

Bassino, J.-P., Broadberry, S., Fukao, K., Gupta, B., & Takashima, M. (2019). Japan and the great divergence, 730–1874. *Explorations in Economic History*, *72*, 1–22.

Bosker, M., Buringh, E., & Van Zanden, J. L. (2013). From baghdad to london : Unraveling urban development in europe, the middle east, and north africa, 800–1800. *Review of Economics and Statistics*, *95*(4), 1418–1437.

Broadberry, S., Campbell, B. M., Klein, A., Overton, M., & Van Leeuwen, B. (2015). *British economic growth, 1270–1870*. Cambridge University Press.

Broadberry, S., Custodis, J., & Gupta, B. (2015). India and the great divergence : An Anglo-Indian comparison of GDP per capita, 1600–1871. *Explorations in Economic History*, *55*, 58–75.

Broadberry, S., Guan, H., & Li, D. D. (2018). China, Europe, and the great divergence : A study in historical national accounting, 980–1850. *The Journal of Economic History*, *78*(4), 955–1000.

Buringh, E., & Van Zanden, J. L. (2009). Charting the “Rise of the West” : Manuscripts and Printed Books in Europe, a long-term Perspective from the Sixth through Eighteenth Centuries. *The Journal of Economic History*, *69*(2), 409–445.

Buyst, E. (2011). Towards Estimates of Long Term Growth in the Southern Low Countries, ca. 1500-1846. *Results presented at the Conference on Quantifying Long Run Economic Development, Venice*, 22–24.

Clark, G. (2015). Markets before economic growth : The grain market of medieval England. *Cliometrica*, *9*(3), 265–287.

De Long, J. B., & Shleifer, A. (1993). Princes and merchants : European city growth before the industrial revolution. *The Journal of Law and Economics*, *36*(2), 671–702.

Galloway, J. A. (2000). *One market or many? London and the grain trade of England*.

Jedwab, R., & Vollrath, D. (2015). Urbanization without growth in historical perspective. *Explorations in Economic History*, *58*, 1–21.

Korndörfer, M., Egloff, B., & Schmukle, S. C. (2015). A large scale test of the effect of social class on prosocial behavior. *PloS one*, *10*(7), e0133193.

Malanima, P. (2011). The long decline of a leading economy : GDP in central and northern Italy, 1300–1913. *European Review of Economic History*, *15*(2), 169–219.

Malinowski, M., & van Zanden, J. L. (2017). Income and its distribution in preindustrial Poland. *Cliometrica*, *11*(3), 375–404.

Pamuk, Ş., & Shatzmiller, M. (2014). Plagues, wages, and economic change in the Islamic Middle East, 700–1500. *The Journal of Economic History*, *74*(1), 196–229.

Pfister, U. (2011). Economic growth in Germany, 1500–1850. *conference on quantifying long run economic development, Venice*, 22–24.

Schön, L., & Krantz, O. (2012). The Swedish economy in the early modern period : Constructing historical national accounts. *European Review of Economic History*, *16*(4), 529–549.

Van Zanden, J. L., & Van Leeuwen, B. (2012). Persistent but not consistent : The growth of national income in Holland 1347–1807. *Explorations in economic history*, *49*(2), 119–130.

***Editor 8. Some readers have noted that it is not possible to know if the facial cues we currently rely on to evaluate trustworthiness based on faces (and which are built into the algorithm) are the same as those used in the past. This was a point that was raised in review, and the limitations discussed there should be clarified in the main manuscript text.***

We agree that it is not possible to have a definite confirmation that the facial cues we currently rely on to evaluate perceived trustworthiness based on faces (and which are built into the algorithm) are the same as those used in the past. Indeed, to know whether this is the case, one would need to have faces that have been labelled by the people of the past. These data are necessarily scarce, but they are not non-existent.

First, qualitative evidence demonstrates that some facial cues have not changed over time. For instance, the French painter Charles Le Brun wrote the *Expressions des passions de l’âme* (Expressions of the passions of the soul) in 1649, right in the middle of the period studied in our article. His drawings of emotions such as anger and contempt (see pictures) suggest that modern people partly use the same facial cues as people of the past to assess whether someone has good intentions toward someone else (online version available at the [Bibliothèque Nationale de France](https://gallica.bnf.fr/ark:/12148/bpt6k1352510)).

However, these are only examples of qualitative evidence. Actually, quantitative evidence in art history also speaks in favor of some degree of universality in facial cues. In a study published this year in *Science Advances*, Cowen and Keltner (2020) studied facial expressions portrayed in artwork created by members of cultures isolated from Western civilization, namely ancient Americas before their contact with Europe. Using data-driven methods, they find that facial expressions depicted in 63 sculptures from the ancient Americas tend to accord with Western expectations for emotions that unfold in specific social contexts. Ancient American sculptures tend to portray at least five facial expressions in contexts predicted by Westerners - including “pain” in torture, “determination”/“strain” in heavy lifting, “anger” in combat, “elation” in social touch, and “sadness” in defeat - supporting the universality of these expressions.

Another example are the masks of Japanese Noh theatre. These masks go back to the 15th c. and have evolved independently of the European tradition. Yet, recent experimental works have demonstrated that they are in line with modern intuitions (Kawai et al., 2013; Miyata et al., 2012). The authors write that their results *'clearly demonstrated that the judgment of emotional expressions of a Noh mask is predominantly made based on the shapes of the mouth, with the mouth of an upward/downward tilted Noh mask expressing sadness/happiness, respectively. Thus, when presented with the face of a Noh mask alone, the categorization of the emotional expressions seems to rely primarily on a biological feature such as the shape of the mouth, rather than on the traditionally formulated styles of the Noh drama. This further seems in agreement with the notion that the recognition of facial expressions is universal and thus is biologically-based, even when the artificial masks are used instead of the human faces.*' (Miyata et al., 2012)

Obviously, further work in experimental art history would be needed. We now acknowledge this limitation in the paper. We propose to add the following sentence to the last paragraph of the discussion:

Finally, several limitations are to be noted. (...) Second, our study is based on the assumption that facial cues used as cues for assessing perceived trustworthiness trustworthiness are shared across time. Although both qualitative evidence (such as pre-modern treatises on facial expression written by painters (Le Brun, 1727) and philosophers (Descartes, 1728)) and quantitative evidence (on ancient American artworks (Cowen & Keltner, 2020) and Japanese ancient theatre (Miyata et al., 2012)) have demonstrated such stability, further work is needed to fully test this assumption.

References

Cowen, A. S., & Keltner, D. (2020). Universal facial expressions uncovered in art of the ancient Americas : A computational approach. *Science advances*, *6*(34), eabb1005.

Descartes, R. (1728). *Les passions de l’âme*.

Le Brun, C. (1727). *Expressions des Passions de l’Ame, représentées en plusieurs têtes gravées d’après les dessins de feu M. Le Brun, premier peintre du Roy*. Jean Audran.

Kawai, N., Miyata, H., Nishimura, R., & Okanoya, K. (2013). Shadows Alter Facial Expressions of Noh Masks. *PLOS ONE*, *8*(8), e71389. https://doi.org/10.1371/journal.pone.0071389

Miyata, H., Nishimura, R., Okanoya, K., & Kawai, N. (2012). The Mysterious Noh Mask : Contribution of Multiple Facial Parts to the Recognition of Emotional Expressions. *PLOS ONE*, *7*(11), e50280. https://doi.org/10.1371/journal.pone.0050280

**Response after review**

Dear Dr Baumard,

I'd like to reiterate our apology for the time it has taken for us to send you the next steps regarding your paper.

We appreciate the comments that you provided in response to the list of concerns that we requested you address.

After reading your replies, we decided to ask two of the reviewers (Reviewers 1 and 2 from the original submission) for further input. We explained to these reviewers the criticisms that had been raised by readers around the paper, and asked them if they would be able to review your responses to these criticisms.

We also attempted to secure an expert who could comment on aspects of the paper’s framing that are related to art history. We contacted several people with this expertise but, unfortunately, none were able to provide a detailed report. One did, however, provide general comments, which we have paraphrased below (these comments follow the original reviewer's new comments).

You will see from the additional comments we gathered that both Reviewers 1 and 2 agree that the paper would benefit from changes to the language used to describe the measure of “trustworthiness,” such that it is clear in all areas of the text that the approach is meant to capture “perceived” and not “actual” trustworthiness. We appreciate that you already proposed such changes in your response but it is clear from the reports we received that there are still areas that require further clarification.

Reviewer 1 also strongly suggests that it is made clearer that perceptions of trustworthiness can be biased by a number of different factors, and that the algorithm was trained on models of perception that are themselves subject to biases. Reviewer 1 has suggested extensions to the disclaimer along these lines, which should be incorporated.

In addition, Reviewer 2 has made suggestions for improving the presentation of the results, and the need to recognise that the effects observed are small, and to provide context for their size.

Please also incorporate the reviewers’ other comments in your response and your proposal for correcting the manuscript.

While we believe that many of the changes made, and the further changes in response to the reviewers’ new comments here, generally cover the points raised by the art historian, we would urge you to ensure that limitations of the paintings datasets and GDP measure are made explicit, and that it is clear from the language used that you are measuring “perceived” and not “actual” trustworthiness.

Below are additional comments from the editorial team, based on some of the changes you had proposed in your previous response:

TITLE

We thank you for proposing a change to the title (’Tracking historical changes in trustworthiness displays in Western Europe using machine learning analyses of facial cues in painting’). However, we are concerned that “trustworthiness displays” may not be entirely clear to our readers, and believe this would be better understood if replaced with “perceived trustworthiness," here and throughout the main text.

We propose the title: “‘Tracking historical changes in perceived trustworthiness in Western Europe using machine learning analyses of facial cues in paintings’.

ABSTRACT

It is not clear from the abstract that you are using perceived trustworthiness of faces as a proxy for “social trust.”  Please ensure this is spelled out for the reader.

It would be useful to replace “building on recent advances in social cognition” with a sentence or two explaining the existing approaches for understanding perceptions of trustworthiness in faces, and your application of that approach here.

Please remove “paralleling the decline of interpersonal violence and the rise of democratic values observed in Western Europe.”

DISCLAIMER

In addition to incorporating the suggestions for your disclaimer provided by Reviewer 1, we would encourage you to modify the statement so that it states both what this tool could be used for, but also what it should not be used for (see, for example: [https://medium.com/@GovAI/a-guide-to-writing-the-neurips-impact-statement-4293b723f832](https://urldefense.proofpoint.com/v2/url?u=https-3A__medium.com_-40GovAI_a-2Dguide-2Dto-2Dwriting-2Dthe-2Dneurips-2Dimpact-2Dstatement-2D4293b723f832&d=DwMFaQ&c=vh6FgFnduejNhPPD0fl_yRaSfZy8CWbWnIf4XJhSqx8&r=KSpTq03cqVsQ14yhHABiKzAY6mM1k0CqIdSssPTUQbk&m=L80H9vD6RIzU_f4BjAqlyErEMCiawrla7Cfw5-fJ-Hc&s=dOJNXofgc8M2P5ywBgSErTdYJMmvDq_gNLMEznjxTmI&e=)).

The editorial team also made minor changes to the wording of the existing disclaimer (including the use of “Human raters” instead of “participants”). Please ensure that revised disclaimer you provide use similar wording:

(edited disclaimer): “The present algorithm was built to mimic how human raters would perceive trustworthiness of faces, based on a set of facial muscles activations. The scores computed by the algorithm approximate human raters’ subjective perceptions of trustworthiness of the processed images based on the pose taken by the individual. It does not provide information about individuals’ actual character, including their trustworthiness.”

EFFECT SIZES

We see that you have commented on the effect sizes associated with the correlation between the algorithm ratings and those of human raters. However, we would also require that discussion of effect sizes for changes in trust over time, and the relationship to GDP, are included.

Please also ensure that in this discussion of effect sizes you clarify in the main text why it is not unexpected to see effects of this size, based on the approach used and the dataset available.

In addition, any relevant details regarding the interpretation of the effect size currently provided only in the original responses to the reviewers should be included in the main text.

DATASET LIMITATIONS

Please ensure that the geographical restriction is also clear in the introduction.

We suggest changes to reorder/streamline the ideas in the paragraph you had planned to include in the discussion:

(edited version) “Finally, several limitations are to be noted. First, one cannot assume that the evolution of trustworthiness displays depicted in this study extends to the larger population of the period. The phenomenon described in this article might, for instance, be limited to the relatively elite, wealthy population represented in the portraits. In line with this possibility is evidence that social attitudes can vary with socioeconomic status (Zwirner & Raihani, 2020; Pepper & Nettle, 2017; Brandt et al., 2015; Holland et al., 2011; Korndörfer et al., 2015; Levine et al., 2008).”

GDP LIMITATIONS

Please include discussion of the limitations of GDP in the main text.

We would also strongly suggest that the book data analysis currently provided in the SI (and referred to in the original response to the reviewers) is included in the main text.

In order to continue, we will require this more extensive response to our previous list of concerns, as well as a revised text using tracked changes to show clearly the proposed corrections; we would like to receive your response within two weeks. We will reach a final decision when we have received your revised responses to the concerns.

Thanks once again for your patience with his process. We hope to be able to make a final decision soon.

**Reviewer 1**

As I wrote in my original review, I thought that this paper was an interesting application of machine learning methods based on modeling of social perception of faces to study historical changes. The approach opens new venues for the study of cultural artifacts.

Unfortunately, the language of the paper appeared to imply that the machine-learning algorithm is capturing real trustworthiness (of the sitter) rather than perceptions of trustworthiness. I regret that I missed this ambiguity of the language. What is obvious in one’s own field is not necessarily obvious in other fields.

Reading the response of the authors to the editors’ concerns, I think that a) the proposed changes/ corrections in title and text are absolutely necessary; and b) should go even further from the very beginning of the paper. The essential point is that the algorithm measures perceptions of trustworthiness and that these perceptions can be dramatically shifted as a function of slight variations in pose, expressions, clothing and so on (for the influence of clothing, see Oh, D., Shafir, E., & Todorov, A. (2020). Economic status cues from clothes affect perceived competence from faces. Nature Human Behavior, 4, 287-293). These points should be stated explicitly in the manuscript.

Moreover, it should be also explicitly stated that the algorithm was trained on models of perceptions. In fact, one view of all of the models we have built in my lab is that they simply capture systematic biases in social perception. These biases have all sorts of pernicious consequences in real life. Although there are some researchers who believe that biases in social perception can have a “kernel of truth,” under closer inspection the kernel is either tiny or non-existent. I review much of the relevant research in my book Face Value, which was specifically motivated to make a case against physiognomy. The facts are that people automatically form impressions from appearance and that there is shared agreement in these impressions. Unfortunately, often these facts are misinterpreted to signify that such impressions based on superficial cues are accurate. In light of this, all of us doing research in this field need to be extremely careful about how we discuss our findings.

Re editorial point 2, the use of the algorithm to measure “real” trustworthiness would be a clear misuse. The disclaimer added by the authors should help, but it should also add that any measures resulting from the algorithm at best approximate what would be the aggregate ratings of a group of raters (see below for biases in terms of the composition of the raters).

There are many other interesting and apt points in the list of editorial concerns, but I see these as secondary, mainly because they do not provide an alternative explanation of the findings.

Point 4. The size of the effects is indeed small. I attribute this to the fact that our models of perceptions built in FaceGen do not represent texture and pigmentation information well. Thus, much information used in judgments of real faces is lost in the process of training a machine-learning algorithm on a model of synthetic faces. But the correlations are significant in the predicted direction, suggesting that some reliable signal is preserved. In general, low reliability should hurt the authors in finding any meaningful relations between the measures of perceived trustworthiness and other variables. It is, of course, possible that very low reliability could lead to the “discovery” of spurious relationships, but this is less likely if the measure correlates with multiple different measures in the predicted direction.

In their response, the authors point out that impressions have a universal and an idiosyncratic component. I would refrain from using the term “universal”. “Shared” is a better term. In fact, partitioning the meaningful variance in ratings of faces shows that for every single social judgment, the meaningful idiosyncratic variance exceeds the meaningful shared variance (for attractiveness, the “proper” appearance-based judgment, the estimates are 50:50). See Martinez, J. E., Funk, F., & Todorov, A. (2020). Quantifying idiosyncratic and shared contributions to judgment. Behavior Research Methods. Published online before print on January 2, doi: 10.3758/s13428-019-01323-0.

Point 6. The algorithm is indeed biased in terms of the composition of the raters and the faces, because it was trained on our models, which are biased. Although we have applied the models to faces of different ethnicities, in the original work we used only Caucasian faces to avoid effects of racial biases on judgments. The faces were also bold and, as a result, more likely to be perceived as males than as females. Our raters were Princeton University students. So clearly, there are a number of biases that go into our models. And since the algorithm was trained on our models, the biases should be present there too (for more recent work that is not limited to specific types of faces and raters, see Oh, D., Dotsch, R., Porter, J., & Todorov, A. (2020). Gender biases in impressions from faces: Empirical studies and computational models. Journal of Experimental Psychology: General, 149, 323-342).

However, this valid concern, which should be acknowledged, cannot explain the authors’ findings. Partly, this is related to point 5 (the portraits are mainly of Caucasian, Western people) and partly this is related to the fact that differences between faces (e.g., male vs. female faces) trump differences between raters (e.g., male vs. female raters) in judgments.

Point 8 is obviously valid. We cannot know for sure what cues people used in the past to make trustworthiness judgments. But assuming that they used different cues, it is not obvious to me how this would explain the findings. With respect to the authors’ response, I don’t find the reference to Charles Le Brun very compelling. He was extremely influential during his time and his work was used as a drawing manual in many art schools across Europe. But after his death, he was extensively criticized about the artificial nature of his drawings (in this particular work). In an ironic way, the debates in the modern emotional expressions literature reflect the questions debated in the 17th and 18th century French Art Academy. The work of Cowen and Keltner is much more compelling.

**Reviewer 2**

I feel that points 2, 5, 6, 7 and 8 have been sufficiently addressed

by the authors.

Regarding point 1:

I assume that a qualifier is added at each instance of "trustworthiness" / "dominance." Alternatively, a sentence at the beginning may clarify that in all further instances the perceived trustworthiness / dominance is intended.

I also noticed some editing problems around lines 45-55.

Regarding point 3:

First, I imagine that information on fit should be added not only to Supplementary Figure 3 but also to other figures with a trend line (e.g. Fig 2, Supp Fig 2, Supp Fig 4?).

The example figure caption contains an error: it should state "retrieved vs. perceived" trustworthiness / dominance and not, as currently, "perceived vs. perceived."

Second, the description of methods (lines 160 and down) are understandable. I am used to the term "lagged" rather than "delayed"(variable), but the text is sufficiently clear.

Third, the information that the scripts are available at the Open Science Framework repository should probably be added to the main text.

Regarding point 4:

Here it seems that the authors are reluctant to comment on effect sizes in the main text. Nonetheless, I agree that it would be useful for readers. In general, while reporting the results it is desirable to account not only for statistical significance of coefficients but also for the substantive size of the effects. In this analysis it applies not just to correlations between algorithm outcome and evaluations, but also to relationships with GDP, social trust, etc.

Dear Editorial Team,

We would like to thank one more time for giving us the opportunity to clarify and thereby improve our manuscript, and we would like to reiterate our apologizes for the misconceptions that were generated. In the revised version of the article, **we clarify every instance of the word ‘trustworthiness’ as referring to ‘perceived trustworthiness’, notably in the article’s title**, and **clearly state the limits of the perception model we used to build our algorithm**. In addition, **we discuss the small sizes of the reported effects**. We also **discuss the limits of the use of historical economic indicators**, and in particular historical GDP, and **report the additional analyses taking the number of book titles per capita as a proxy economic wellbeing**, which we conducted in a previous round of review. Finally, we made **substantial additions to the disclaimer** in order to highlight what the developed algorithm could be used for and should not be used for as well as its limits. All these changes are highlighted in the attached revised manuscript.

We hope and believe that these changes make substantial improvement to the article, respond to the criticisms raised by readers and clarify the sources of much confusion.

Below you will find the point-by-point responses to the Editorial Team and the Reviewers’ comments.

Best regards,

*TITLE*

*We thank you for proposing a change to the title (’Tracking historical changes in trustworthiness displays in Western Europe using machine learning analyses of facial cues in painting’). However, we are concerned that “trustworthiness displays” may not be entirely clear to our readers, and believe this would be better understood if replaced with “perceived trustworthiness," here and throughout the main text.*

*We propose the title: “‘Tracking historical changes in perceived trustworthiness in Western Europe using machine learning analyses of facial cues in paintings’.*

We would like to thank the editorial team for this suggestion that indeed clarify the title of our article.

*ABSTRACT*

*It is not clear from the abstract that you are using perceived trustworthiness of faces as a proxy for “social trust.” Please ensure this is spelled out for the reader.*

*It would be useful to replace “building on recent advances in social cognition” with a sentence or two explaining the existing approaches for understanding perceptions of trustworthiness in faces, and your application of that approach here.*

We agree that these two key elements of our research should be made clear from the abstract. We made these changes in the revised abstract:

**Revised abstract:**

Social trust is linked to a host of positive societal outcomes, including improved economic performance, lower crime rates and more inclusive institutions. Yet, the origins of trust remain elusive, partly because social trust is difficult to document in time. Building on recent advances in social cognition, we design an algorithm to automatically estimate ratings of perceived trustworthiness from specific facial cues (such as muscle contractions associated with smiling) detected in European portraits in large historical databases. We used this measure as a proxy of social trust in history. Our results show that estimated levels of perceived trustworthiness in portraits increased over the period 1500–2000. Further analyses suggest that this rise of perceived trustworthiness is associated with increased living standards.

*Please remove “paralleling the decline of interpersonal violence and the rise of democratic values observed in Western Europe.”*

We agree that the abstract is improved without this sentence, we deleted it from the revised version of the abstract.

*DISCLAIMER*

*In addition to incorporating the suggestions for your disclaimer provided by Reviewer 1, we would encourage you to modify the statement so that it states both what this tool could be used for, but also what it should not be used for (see, for example:* [*https://medium.com/@GovAI/a-guide-to-writing-the-neurips-impact-statement-4293b723f832*](https://medium.com/@GovAI/a-guide-to-writing-the-neurips-impact-statement-4293b723f832)*).*

*The editorial team also made minor changes to the wording of the existing disclaimer (including the use of “Human raters” instead of “participants”). Please ensure that revised disclaimer you provide use similar wording: and incorporated both a statement on the limits of the algorithm which better explain how it was constructed, as suggest by Reviewer 1, and highlighted the potential use and misuses of our algorithm.*

We would like to thank the editorial team for these editions. We changed the disclaimer:

**Disclaimer:**

The algorithm was built to estimate how human raters would rate perceive the trustworthiness of faces. It can be used in scientific research for this purpose. The algorithm cannot, and was not intended for, quantifying the actual trustworthiness of an individual. The algorithm should therefore not be used in any real world settings, and in particular should not be used in an attempt to determine the trustworthiness of a person.

*EFFECT SIZES*

*We see that you have commented on the effect sizes associated with the correlation between the algorithm ratings and those of human raters. However, we would also require that discussion of effect sizes for changes in trust over time, and the relationship to GDP, are included.*

We agree that these elements will certainly help the reader to have an accurate estimate of the evidenced effects. In this revised version of the manuscript, we explicitly commented on the size of the effect of time and GDP.

Lines 142-145:

Although the increase of perceived trustworthiness is small, these results are consistent with more qualitative works documenting a so-called ‘Smile Revolution’ ^28^ and a rise of prosocial displays in paintings and in novels^29^.

Lines 214-219:

These results provide evidence in favor of the association between economic wealth and social trust at the society level. However, due to the small effect sizes and the limitations of the historical economic indicators, as well as to the fact that GDP per capita is only a partial measure of wealth (which does not account, for example, for inequalities in wealth distribution), we replicated our analyses with an alternative variable known to be associated with countries’ wealth: the number of book titles per capita.

*Please also ensure that in this discussion of effect sizes you clarify in the main text why it is not unexpected to see effects of this size, based on the approach used and the dataset available.*

*In addition, any relevant details regarding the interpretation of the effect size currently provided only in the original responses to the reviewers should be included in the main text.*

We agree that adding these elements will help the readers to have a more informed understanding of our results. In the revised version of the manuscript, we included all the elements that can help to understand why the size of the association between human ratings and the scores produced by our algorithm are not completely unexpected.

Lines 271-280

At this point, we have to acknowledge the small correlation between the perceived trustworthiness ratings provided by human raters and those retrieved by our algorithm. However, this small effect size is not completely unexpected. First, the avatars on which the algorithm was trained did not represent the texture of the faces, even though this information may influence human raters’ evaluations. Similarly, the avatars are bold and our algorithm is thus blind to haircut, even though these cues are known to influence first impressions from faces (see e.g.,^27^). Finally, our algorithm was trained to generate ratings of perceived trustworthiness based on the facial features that represent the shared component of first impressions from faces. Indeed, individuals rely on both shared and idiosyncratic features when forming a first impression on a new face, and our algorithm was designed to produce scores only based on the former.

*DATASET LIMITATIONS*

*Please ensure that the geographical restriction is also clear in the introduction.*

Thank you for pointing that this information was lacking at the beginning of the paper, we added the geographical restriction in the introduction.

Lines 85-86

More precisely, we apply recent machine-learning methods to extract quantitative information about the evolution of social cues contained in Western European portraits.

*We suggest changes to reorder/streamline the ideas in the paragraph you had planned to include in the discussion:*

*(edited version) “Finally, several limitations are to be noted. First, one cannot assume that the evolution of trustworthiness displays depicted in this study extends to the larger population of the period. The phenomenon described in this article might, for instance, be limited to the relatively elite, wealthy population represented in the portraits. In line with this possibility is evidence that social attitudes can vary with socioeconomic status (Zwirner & Raihani, 2020; Pepper & Nettle, 2017; Brandt et al., 2015; Holland et al., 2011; Korndörfer et al., 2015; Levine et al., 2008).”*

We would like to thank you for this proposition, we reordered the last paragraph of the discussion following your recommendations.

Lines 281-290:

Finally, several limitations are to be noted. First, one cannot assume that the evolution of perceived trustworthiness depicted in this study extends to the larger population of the period. The phenomenon described in this article might, for instance, be limited to the relatively elite, wealthy population represented in the portraits. In line with this possibility, there is evidence that social attitudes can vary with socioeconomic status ^53–56^.

*GDP LIMITATIONS*

*Please include discussion of the limitations of GDP in the main text.*

We agree that these limitations are important to explicitly state. In the revised version of the manuscript we discuss the limitation of using historical GDP as a measure of wealth both in the result and in the discussion sections.

Lines 215-219:

However, due to the small effect sizes and the limitations of the historical economic indicators^47,48^, as well as to the fact that GDP per capita is only a partial measure of wealth (which does not account, for example, for inequalities in wealth distribution^49^), we replicated our analyses with an alternative variable known to be associated with countries’ wealth: the number of book titles per capita.

Lines 288-290:

Third, times series of GDP per capita and living standards are only estimates, and their precision may fluctuate throughout the studied time period and fail to fully capture the evolution of living standards and inequalities.

*We would also strongly suggest that the book data analysis currently provided in the SI (and referred to in the original response to the reviewers) is included in the main text.*

We would like to thank the editorial team for this suggestion, we included these analyses in the result section of the revised version of the manuscript.

Lines 221-227:

Supporting the analyses conducted with GDP per capita, we found a significant positive association between the number of book titles per capita and the level of perceived trustworthiness in the portraits of the National Portrait Gallery (affluence only model: b = .35 ± .06, z = 6.15, p <.001; model controlling for time: b = .21 ± .06, z = 3.45, p = .001) and of the Web Gallery of Art, although not robust to the inclusion of time in this latter case (affluence only model: b = .29 ± .10, z = 2.77, p = .006; model controlling for time: b = .14 ± .11, z = 1.26, p = .208).

**Reviewer 1**

*As I wrote in my original review, I thought that this paper was an interesting application of machine learning methods based on modeling of social perception of faces to study historical changes. The approach opens new venues for the study of cultural artifacts.*

*Unfortunately, the language of the paper appeared to imply that the machine-learning algorithm is capturing real trustworthiness (of the sitter) rather than perceptions of trustworthiness. I regret that I missed this ambiguity of the language. What is obvious in one’s own field is not necessarily obvious in other fields.*

We would like to thank Prof. Todorov for his help in the reviewing process and we would like to apologize for the many unintended misunderstandings and confusions caused by the initial version of the article.

*Reading the response of the authors to the editors’ concerns, I think that a) the proposed changes/ corrections in title and text are absolutely necessary; and b) should go even further from the very beginning of the paper. The essential point is that the algorithm measures perceptions of trustworthiness and that these perceptions can be dramatically shifted as a function of slight variations in pose, expressions, clothing and so on (for the influence of clothing, see Oh, D., Shafir, E., & Todorov, A. (2020). Economic status cues from clothes affect perceived competence from faces. Nature Human Behavior, 4, 287-293). These points should be stated explicitly in the manuscript.*

We agree with Prof. Todorov of the importance of making this notion clear to the readers from the introduction.

Lines 89-91:

Crucially, this algorithm does not provide information on a person’s face but rather on the way this face is likely to be perceived by others, based on a specific image.

*Moreover, it should be also explicitly stated that the algorithm was trained on models of perceptions. In fact, one view of all of the models we have built in my lab is that they simply capture systematic biases in social perception. These biases have all sorts of pernicious consequences in real life. Although there are some researchers who believe that biases in social perception can have a “kernel of truth,” under closer inspection the kernel is either tiny or non-existent. I review much of the relevant research in my book Face Value, which was specifically motivated to make a case against physiognomy. The facts are that people automatically form impressions from appearance and that there is shared agreement in these impressions. Unfortunately, often these facts are misinterpreted to signify that such impressions based on superficial cues are accurate. In light of this, all of us doing research in this field need to be extremely careful about how we discuss our findings.*

We agree that it is of paramount importance for understanding our findings and research project that the model on which our algorithm is built is a model of face perception. We included this statement in the introduction and in the long version of the disclaimer displayed on the OSF file of the project.

Lines 86-89:

The algorithm is built on models of human perception of faces to generate automatic human-like ratings of perceived trustworthiness based on the muscle contractions (facial action units) detected in facial displays in portraits using the open software OpenFace^23^.

Long disclaimer on OSF:

This algorithm was constructed based on a perception model, created from evaluations provided by Princeton University students on Caucasian faces, it thus incorporates the biases present in this population when providing first impressions ratings on these faces.

*Re editorial point 2, the use of the algorithm to measure “real” trustworthiness would be a clear misuse. The disclaimer added by the authors should help, but it should also add that any measures resulting from the algorithm at best approximate what would be the aggregate ratings of a group of raters (see below for biases in terms of the composition of the raters).*

We would like to thank Prof. Todorov for this suggestion, we clearly state this point in the revised version of our disclaimer.

Short disclaimer:

The algorithm was built to estimate how human raters would rate the perceived trustworthiness of faces. It can be used in scientific research for this purpose. The algorithm does not quantify the actual trustworthiness of an individual, and was not intended for this purpose. The algorithm should therefore not be used in any real-world settings, and in particular should not be used in an attempt to determine the trustworthiness of a person.

Long disclaimer on OSF:

This tool can be used to estimate how human raters would judge specific images of faces.

Long disclaimer on OSF:

Importantly, this algorithm has no significant benefit compared to collecting actual human ratings for the evaluations of contemporary pictures as well as of images from which it is possible to remove contextual or historical information.

*There are many other interesting and apt points in the list of editorial concerns, but I see these as secondary, mainly because they do not provide an alternative explanation of the findings.*

*Point 4. The size of the effects is indeed small. I attribute this to the fact that our models of perceptions built in FaceGen do not represent texture and pigmentation information well. Thus, much information used in judgments of real faces is lost in the process of training a machine-learning algorithm on a model of synthetic faces. But the correlations are significant in the predicted direction, suggesting that some reliable signal is preserved. In general, low reliability should hurt the authors in finding any meaningful relations between the measures of perceived trustworthiness and other variables. It is, of course, possible that very low reliability could lead to the “discovery” of spurious relationships, but this is less likely if the measure correlates with multiple different measures in the predicted direction.*

We would like to thank Prof. Todorov for this comment. We added this point of discussion in the revised version of the article in order to provide to the readers all the elements that are necessary to have an informed perspective on our result. On the other hand, in the revised version of the article, we clearly present the size of the evidenced effects and the importance of the converging evidence reported.

Lines 142-145:

Although the increase of perceived trustworthiness is small, these results are consistent with more qualitative works documenting a so-called ‘Smile Revolution’ ^28^ and a rise of prosocial displays in paintings and in novels^29^.

Lines 214-219:

These results provide evidence in favor of the association between economic wealth and social trust at the society level. However, due to the small effect sizes and the limitations of the historical economic indicators^47,48^, as well as to the fact that GDP per capita is only a partial measure of wealth (which does not account, for example, for inequalities in wealth distribution^49^), we replicated our analyses with an alternative variable known to be associated with countries’ wealth: the number of book titles per capita.

Lines 271-274:

At this point, we have to acknowledge the small correlation between the perceived trustworthiness ratings provided by human raters and those retrieved by our algorithm. However, this small effect size is not completely unexpected. First, the avatars on which the algorithm was trained did not represent the texture of the faces, even though this information may influence human raters’ evaluations.

*In their response, the authors point out that impressions have a universal and an idiosyncratic component. I would refrain from using the term “universal”. “Shared” is a better term. In fact, partitioning the meaningful variance in ratings of faces shows that for every single social judgment, the meaningful idiosyncratic variance exceeds the meaningful shared variance (for attractiveness, the “proper” appearance-based judgment, the estimates are 50:50). See Martinez, J. E., Funk, F., & Todorov, A. (2020). Quantifying idiosyncratic and shared contributions to judgment. Behavior Research Methods. Published online before print on January 2, doi: 10.3758/s13428-019-01323-0.*

We agree that the use of ‘universal’ was not appropriate. In the revised version of the manuscript, when discussing the reliability of our algorithm, we use the notion of ‘shared’.

Lines 276-280:

Finally, our algorithm was trained to generate ratings of perceived trustworthiness based on the facial features that represent the shared component of first impressions from faces. Indeed, individuals rely on both shared and idiosyncratic features when forming a first impression on a new face, and our algorithm was designed to produce scores only based on the former.

*Point 6. The algorithm is indeed biased in terms of the composition of the raters and the faces, because it was trained on our models, which are biased. Although we have applied the models to faces of different ethnicities, in the original work we used only Caucasian faces to avoid effects of racial biases on judgments. The faces were also bold and, as a result, more likely to be perceived as males than as females. Our raters were Princeton University students. So clearly, there are a number of biases that go into our models. And since the algorithm was trained on our models, the biases should be present there too (for more recent work that is not limited to specific types of faces and raters, see Oh, D., Dotsch, R., Porter, J., & Todorov, A. (2020). Gender biases in impressions from faces: Empirical studies and computational models. Journal of Experimental Psychology: General, 149, 323-342).*

*However, this valid concern, which should be acknowledged, cannot explain the authors’ findings. Partly, this is related to point 5 (the portraits are mainly of Caucasian, Western people) and partly this is related to the fact that differences between faces (e.g., male vs. female faces) trump differences between raters (e.g., male vs. female raters) in judgments.*

We agree with Prof. Todorov that this concern should be acknowledged. Following the editors’ recommendations, we do not present it in the revised version of the article but we included it in the long version of the disclaimer displayed on the OSF file of the project.

Long disclaimer on OSF:

This algorithm was constructed based on a perception model, created from evaluations provided by Princeton University students on Caucasian faces, it thus incorporates the biases present in this population when providing first impressions ratings on these faces.

*Point 8 is obviously valid. We cannot know for sure what cues people used in the past to make trustworthiness judgments. But assuming that they used different cues, it is not obvious to me how this would explain the findings. With respect to the authors’ response, I don’t find the reference to Charles Le Brun very compelling. He was extremely influential during his time and his work was used as a drawing manual in many art schools across Europe. But after his death, he was extensively criticized about the artificial nature of his drawings (in this particular work). In an ironic way, the debates in the modern emotional expressions literature reflect the questions debated in the 17th and 18th century French Art Academy. The work of Cowen and Keltner is much more compelling.*

We would like to thank Prof. Todorov for this insightful comment. In the revised version of the article, we removed the reference to Charles Le Brun and added the following citation on emotion expression:

Cowen, A.S., Keltner, D., Schroff, F. *et al.* Sixteen facial expressions occur in similar contexts worldwide. *Nature* **589,** 251–257 (2021). https://doi.org/10.1038/s41586-020-3037-7

Lines 285-286:

Second, our study is based on the assumption that facial cues that are used as cues to assess perceived trustworthiness are shared across time. Although recent evidence^57-59^ points towards such a stability, further work is needed to fully test this assumption.

**Reviewer 2**

*I feel that points 2, 5, 6, 7 and 8 have been sufficiently addressed*

*by the authors.*

We would like to thank Dr. Mikucka for her help in the review process and we would like to apologize for the unintended misconceptions and confusions generated by the initial version of our article.

*Regarding point 1:*

*I assume that a qualifier is added at each instance of "trustworthiness" / "dominance." Alternatively, a sentence at the beginning may clarify that in all further instances the perceived trustworthiness / dominance is intended.*

We would like to thank Dr. Mikucka for this suggestion. In addition to changing every occurrence of ‘trustworthiness’ and ‘dominance’, we also included an explicit statement on this point in the introduction.

Lines 126-128:

In this article, all occurrences of the words ‘trustworthiness’ and ‘dominance’ refer to subjective perceptions of trustworthiness and dominance from faces and not to individuals’ actual level of trustworthiness or dominance.

*I also noticed some editing problems around lines 45-55.*

We are sorry for this editing issues in our response to the editorial team.

*Regarding point 3:*

*First, I imagine that information on fit should be added not only to Supplementary Figure 3 but also to other figures with a trend line (e.g. Fig 2, Supp Fig 2, Supp Fig 4?).*

*The example figure caption contains an error: it should state "retrieved vs. perceived" trustworthiness / dominance and not, as currently, "perceived vs. perceived."*

We agree with Dr. Mikucka that the information on fit should be in every caption presenting a trend line, and we are sorry that the fact that we included this information in all these captions was not clear in our response document. We indeed added fit information in the captions of Figure 2, Supplementary Figure 2, Supplementary Figure 3, Supplementary Figure 4 and Supplementary Figure 5. We also would like to thank Dr. Mikucka for noticing the typo in Supplementary Figure 3 caption, we corrected it.

Figure 2 caption:

**Figure 2** – Effect of time and affluence on ratings of perceived trustworthiness across time

Time was associated with an increase of ratings of perceived trustworthiness in both the National Portrait Gallery (A – data are aggregated by decade; regression line corresponds to the analysis on individual portraits) and the Web Gallery of Art (B – data are aggregated by country and decade; regression line corresponds to the analysis on individual portraits). Increased GDP per capita predicted increased ratings of perceived trustworthiness better than time only-models both in the National Portrait Gallery (C – data are aggregated by GDP; regression line corresponds to the analysis on individual portraits) and the Web Gallery of Art (D– data are aggregated by country and GDP; regression line correspond to the analysis on individual portraits). Data are represented as mean values, error bars represent standard error to the means, the red line corresponds to the estimated effect in the regression adjusting for gender, age (for the National Portrait Gallery only) and perceived dominance, the shaded area represents the standard error to the mean of these effects. Source data are provided as raw data and scripts on the online depository.

Supplementary Figure 2 caption:

**Supplementary Figure 2** Correlation between the avatars’ actual level of perceived trustworthiness and perceived dominance in the test set and the computed perceived trustworthiness (A; Pearson correlation: *r* = .85, *t*(75) = 14.17, *p* < .001) and perceived dominance (B; Pearson correlation: *r* = .86, *t*(75) = 14.72, *p* < .001) based on the model optimized on the training set only. Source data are provided as raw data and scripts on the online depository.

Supplementary Figure 3 caption:

**Supplementary Figure 3** Correlation between participants’ ratings of trustworthiness and dominance displays in the three databases providing subjective ratings of perceived trustworthiness and perceived dominance (the Chicago Face Database, the Oslo Face Database and the Karolinska Face Database) and the retrieved perceived trustworthiness (**A**, Pearson correlation: *r* = .22, *t*(768) = 6.19, *p* < .001) and retrieved perceived dominance (**B**, Pearson correlation: *r* = .16, *t*(769) = 4.54, *p* < .001) levels estimated using the Facial Action Units detected by Open Face and our random-forest model. Source data are provided as raw data and scripts on the online depository.

Supplementary Figure 4 caption:

**Supplementary Figure 4** Recovery of classical effects of gender (**A-B**, Student t-test: perceived trustworthiness t(972) = 2.67, p = .008; perceived dominance: t(972) = -3.63, p <.001), emotion (**C-D**, two-level linear regression: perceived trustworthiness: *t*(167) = 10.64, *p* <.001; perceived dominance: t(167) = 9.42, p <.001), head orientation (**E-F**; Pearson correlations: perceived trustworthiness r = .29, t(1500) = 11.51, p <.001; perceived dominance: r = 0.34, t(1500) = 13.79, p <.001) and age (**G-H**, Pearson correlations: perceived trustworthiness: r = -.12, t(518) = -2.68, p = .008; perceived dominance: r = 0.16, t(518) = 3.70, p < .001) in the perceived trustworthiness and perceived dominance estimates computed using our random forest algorithm. In the boxplots (**A-D**), the centre line corresponds to the median, the lower and upper bounds of the box to the 25th and 75th percentiles and the whiskers to the largest and lowest values in a limit of 1.5 times the inter-quartile range from the box bounds. Source data are provided as raw data and scripts on the online depository.

Supplementary Figure 5 caption:

**Supplementary Figure 5 Results on natural images A-B** Recovery of the classical effects of gender in Google Image portraits of ‘Women’ (*N* = 304 images) and ‘Men’ (*N* = 330 images); **C-E** Recovery of the classical gender (**C-D**) and party (**E**) effects on the portraits of the House of the Representatives (women: *N* = 85 images; men: *N* = 334 images; democrats: *N* = 182 images; republicans: *N* = 237 images). The centre line corresponds to the median, the lower and upper bounds of the box to the 25th and 75th percentiles and the whiskers to the largest and lowest values in a limit of 1.5 times the inter-quartile range from the box bounds. Source data are provided as raw data and scripts on the online depository.

*Second, the description of methods (lines 160 and down) are understandable. I am used to the term "lagged" rather than "delayed"(variable), but the text is sufficiently clear.*

We would like to thank Dr. Mikucka for this comment, following the Editor’s recommendation, we did change ‘delayed’ into ‘lagged’ for this manuscript but we take this comment into account in our future work.

*Third, the information that the scripts are available at the Open Science Framework repository should probably be added to the main text.*

We would like to thank Dr. Mikucka for this suggestion. However, the Editorial Team explained to us that the existence of a ‘data availability statement’ was sufficient to present the OSF repository.

*Regarding point 4:*

*Here it seems that the authors are reluctant to comment on effect sizes in the main text. Nonetheless, I agree that it would be useful for readers. In general, while reporting the results it is desirable to account not only for statistical significance of coefficients but also for the substantive size of the effects. In this analysis it applies not just to correlations between algorithm outcome and evaluations, but also to relationships with GDP, social trust, etc.*

We agree that this information can help the reader to have a clearer picture of our results. In the revised version of the article, we explicitly comment the effect of time and GDP. Following the editors’ suggestions, we did not further comment on the association between our measure and societies’ level of general social trust.

Lines 142-145:

Although the increase of perceived trustworthiness is small, these results are consistent with more qualitative works documenting a so-called ‘Smile Revolution’ ^28^ and a rise of prosocial displays in paintings and in novels^29^.

Lines 214-219:

These results provide evidence in favor of the association between economic wealth and social trust at the society level. However, due to the small effect sizes and the limitations of the historical economic indicators, as well as to the fact that GDP per capita is only a partial measure of wealth (which does not account, for example, for inequalities in wealth distribution), we replicated our analyses with an alternative variable known to be associated with countries’ wealth: the number of book titles per capita.

**Art historian (paraphrased)**

We would like to thank the editorial team for having contacted art historians to help us improve our work and we would like to thank the art historian for these comments. In the revised version of the article, we made the changes in order to account for all these comments.

*The NPG dataset has limitations. It may not be representative in terms of the types of faces available in the dataset, as well as the approaches used to create the portraits—e.g. painting vs other media.*

We agree that the National portrait gallery is far more diverse than the subsample of painted portraits we analyzed in our article. In the revised version of the article we make this information clear to the reader.

Lines 430-433:

We limited our analysis to paintings, excluding other medium types at the National Portrait Gallery, such as drawings, sculptures and photographs. In addition, only portraits for which the image was available on the website of the National Portrait Gallery were analyzed (3152 over 3161 paintings).

*There may also be potential preselection issues e.g. related to paintings provided by relatives vs the sitters.*

We would like to thank the expert for this comment. Indeed, this element could create variations within our dataset, we acknowledge this element in the revised version of the article.

Lines 441-445:

Importantly, in order to ensure that the portraits accurately reflected the level of trust at the time the portrait was painted and to avoid re-interpretation of past historical figures, only portraits painted during the sitter’s lifetime were analyzed (number of analysed portraits: *N* = 1962), however we did not control for the provider of the portraits (e.g., purchased, transferred from another museum or given by a private donator).

*There are limitations to the use of GDP. Caution is needed in making strong claims about the use of this measure for understanding prosperity over time. In general, more interpretation is needed regarding the potential changes in measures over time.*

We agree that this is an important limitation of our work. In the revised version of the article, we explicitly acknowledge these limitations.

Lines 288-290:

Third, times series of GDP per capita and living standards are only estimates, and their precision may fluctuate throughout the studied time period and fail to fully capture the evolution of living standards and inequalities.

*Issues with claims that what is being measure is “trustworthiness.”*

We agree that the way the original article was phrased may lead readers to interpret our results as if we were making claims about individuals’ actual trustworthiness. We are sorry for this misunderstanding. In the revised version of the article, we corrected every instance of ‘trustworthiness’ to which the adjective ‘perceived’ was lacking and added a statement at the beginning of the manuscript on the fact that we are referring only to ‘perceived trustworthiness’. In addition, we included statements on the fact that our algorithm is based on a model of perception as well as a disclaimer on how this algorithm is constructed, what it can be used for and what it should not be used for.

Lines 135-137:

In this article, all occurrences of the words ‘trustworthiness’ and ‘dominance’ refer to subjective perceptions of trustworthiness and dominance from faces and not to individuals’ actual level of trustworthiness or dominance.

Lines 126-128:

The algorithm is built on models of human perception of faces to generate automatic human-like ratings of perceived trustworthiness based on the muscle contractions (facial action units) detected in facial displays in portraits using the open software OpenFace^23^. Crucially, this algorithm does not provide information on a person’s face but rather on the way this face is likely to be perceived by others, based on a specific image. Indeed, first impressions from faces are highly sensitive to factors such as variations in lighting and pose.

Disclaimer:

The algorithm was built to estimate how human raters would rate the perceived trustworthiness of faces. It can be used in scientific research for this purpose. The algorithm does not quantify the actual trustworthiness of an individual, and was not intended for this purpose. The algorithm should therefore not be used in any real-world settings, and in particular should not be used in an attempt to determine the trustworthiness of a person.

Long disclaimer on OSF:

The present algorithm was built to mimic how human raters would perceive trustworthiness of faces, based on a set of facial muscles activations. This algorithm was constructed based on a perception model, created from evaluations provided by Princeton University students on Caucasian faces, it thus incorporates the biases present in this population when providing first impressions ratings on these faces.

The scores computed by the algorithm approximate human raters’ subjective perceptions of trustworthiness of the processed images based on the pose taken by the individual. It does not provide information about individuals’ actual character, including their trustworthiness. Therefore, it cannot be used to infer the actual level of trustworthiness of a person. Since the algorithm is highly sensitive to factors such as lighting and pose, it cannot be used to infer the general perception of an individual outside the precise portrait processed by the algorithm.

This tool can be used to estimate how human raters would judge specific images of faces. It is interesting to assess the perception of portraits, which include cultural or historical elements (such as specific outfits, haircut, image quality or painting style) that contemporary raters may incorporate in their evaluations and which may not make evaluations comparable between time periods. It can be used to conduct historical and cross-national trend analyses based on large databases of portraits and pictures. However, as the estimation of muscle activation on which this tool is based on can be influenced by sitters’ fixed facial features, this tool cannot be used to infer the specific social intentions of a person, even on a specific picture.

Importantly, this algorithm has no significant benefit compared to collecting actual human ratings for the evaluations of contemporary pictures as well as of images from which it is possible to remove contextual or historical information.
